# Heterogeneous associations of multiplexed environmental factors and multidimensional aging metrics

**DOI:** 10.1038/s41467-024-49283-0

**Published:** 2024-06-10

**Authors:** Fan Pu, Weiran Chen, Chenxi Li, Jingqiao Fu, Weijing Gao, Chao Ma, Xingqi Cao, Lingzhi Zhang, Meng Hao, Jin Zhou, Rong Huang, Yanan Ma, Kejia Hu, Zuyun Liu

**Affiliations:** 1 Center for Clinical Big Data and Analytics of the Second Affiliated Hospital, and Department of Big Data in Health Science School of Public Health, The Key Laboratory of Intelligent Preventive Medicine of Zhejiang Province, Zhejiang University School of Medicine, Hangzhou, 310058 Zhejiang China; 2 Ocean College, Zhejiang University, Zhoushan, 316021 Zhejiang China; 3 School of Economics and Management, Southeast University, Nanjing, 211189 Jiangsu China; 4 State Key Laboratory of Genetic Engineering, Collaborative Innovation Center for Genetics and Development, School of Life Sciences and Human Phenome Institute, Fudan University, Shanghai, 200433 China; 5 Key Laboratory of Environmental Stress and Chronic Disease Control & Prevention, Ministry of Education, China Medical University; Department of Biostatistics and Epidemiology, School of Public Health, China Medical University, Shenyang, 110122 Liaoning China; 6 Department of Big Data in Health Science School of Public Health, Zhejiang University School of Medicine, Hangzhou, 310058 Zhejiang China

**Keywords:** Geriatrics, Environmental impact, Risk factors, Epidemiology

## Abstract

Complicated associations between multiplexed environmental factors and aging are poorly understood. We manipulated aging using multidimensional metrics such as phenotypic age, brain age, and brain volumes in the UK Biobank. Weighted quantile sum regression was used to examine the relative individual contributions of multiplexed environmental factors to aging, and self-organizing maps (SOMs) were used to examine joint effects. Air pollution presented a relatively large contribution in most cases. We also found fair heterogeneities in which the same environmental factor contributed inconsistently to different aging metrics. Particulate matter contributed the most to variance in aging, while noise and green space showed considerable contribution to brain volumes. SOM identified five subpopulations with distinct environmental exposure patterns and the air pollution subpopulation had the worst aging status. This study reveals the heterogeneous associations of multiplexed environmental factors with multidimensional aging metrics and serves as a proof of concept when analyzing multifactors and multiple outcomes.

## Abstract (web summary)

Complicated associations between multiplexed environmental factors and aging are poorly understood. Here, authors show heterogeneous associations of multiplexed environmental factors with multidimensional aging metrics.

## Introduction

Accelerated aging is a crucial risk factor for various chronic diseases and mortality^1^. Given that aging is a complicated multisystemic process, a single aging biomarker may not comprehensively and accurately portray the whole landscape of the personal aging process due to the individual heterogeneity of cells, tissues, and organs^2^. Moreover, considering that persons may hold varying rates of aging^3^, using chronological age to measure one’s aging process may be arbitrary and opinionated. In fact, aging could be characterized by various physiological phenotypes. Many domain-specific aging metrics have been developed and widely used, such as the brain (e.g., brain age^4^) and physical functioning (e.g., frailty phenotype score^5,6^). Additionally, we have recently developed a composite aging metric, phenotypic age (PhenoAge), derived from multisystemic chemistry biomarkers to reflect changes in multiple dimensions, including body composition, homeostatic mechanisms, and energetics over time. These aging metrics can capture morbidity and mortality risk beyond chronological age^5,7–11^, thus providing unique opportunities to investigate risk factors for aging processes and subsequently inform preventive programs against aging^8,12–14^. Considering the heterogeneous biological aging process across individuals and organs^15^, it is necessary to examine whether certain factors have varying effects on multidimensional aging, rather than evenly and synchronously.

Given that numerous exposures throughout the life course jointly depict the multifaceted picture of aging, understanding the factors contributing to aging is critical to postponing aging and decreasing multiple chronic disease risks. We have previously demonstrated the contributions of various factors to aging, including genetics^12^, unhealthy lifestyles^12,13^, life course adversities^13,14^, and certain chemicals^16^, with limited focus on environmental factors, which are considerably modifiable contributors^17–19^. Among the few studies on environmental exposures and aging, the majority only evaluated a single environmental factor^20–23^. However, individuals are exposed to multiplexed environmental factors rather than a single exposure in reality. Focusing on one of the multiplexed correlated factors may result in an erroneous estimation of the potential association of environmental factors with aging. Hence, considering environmental factors holistically and even subdividing potential specific environmental exposure patterns among populations is critical to deepening the understanding of the extent and how multiplexed environmental factors jointly contribute to aging, as well as revealing the potential variance in aging caused by environmental inequality.

**Fig. 1:**
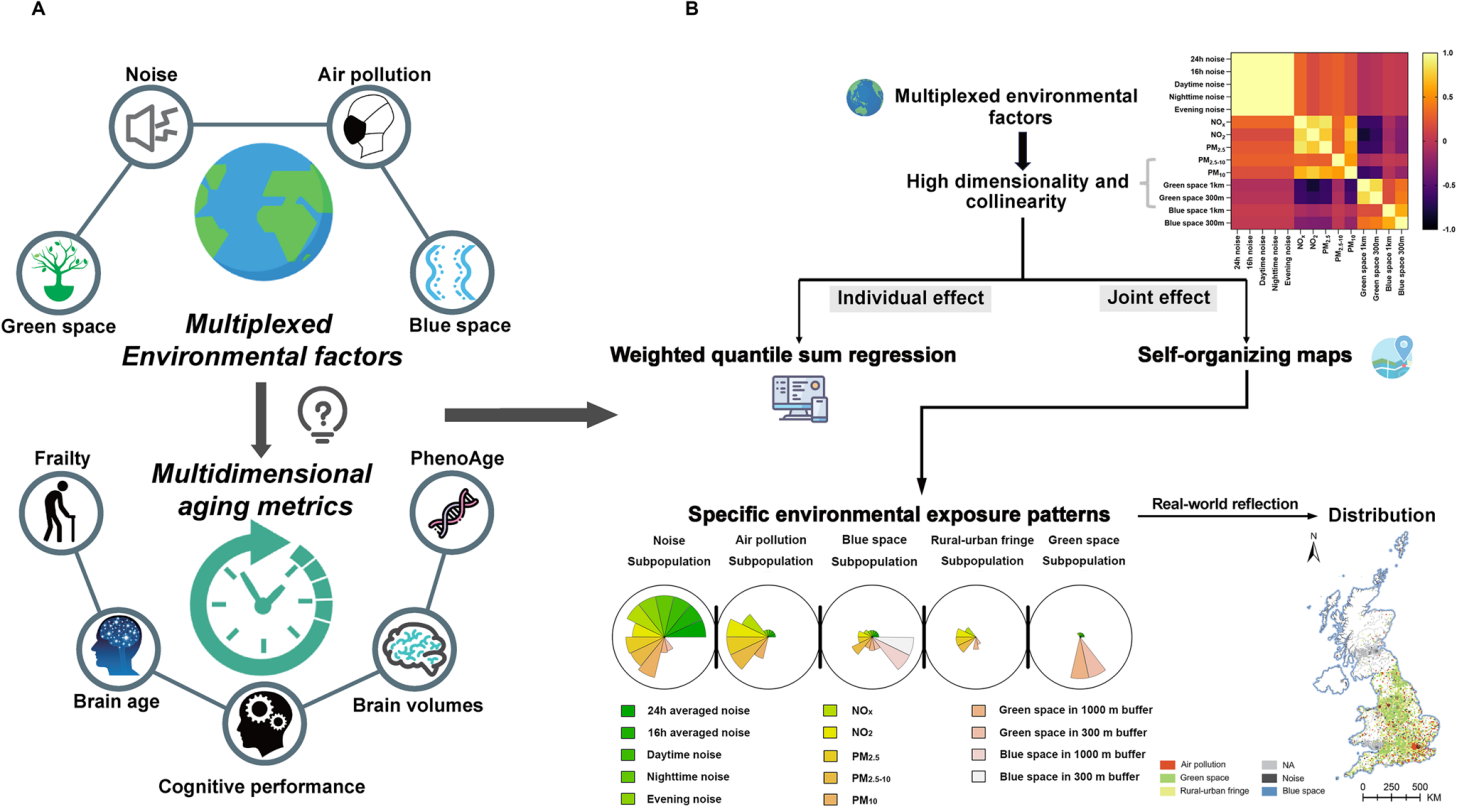
Overview of the study design. **A** The complex associations with multiplexed environmental factors and multidimensional aging metrics**. B** Weighted quantile sum regression (WQS) and self-organizing maps (SOM) were performed to deal with the high dimensionality and collinearity of multiplexed environmental factors and figured out the individual and joint effects of multiplexed environmental factors on aging, respectively. Five subpopulations with specific environmental exposure patterns were distinguished and were reflected in exact locations on the UK map. Cartoon figures can be freely downloaded at https://www.iconfont.cn/.

In this work, we conducted a proof-of-concept study (Fig. 1) using data from the UK Biobank (UKB), a large population-based cohort study with ~500,000 participants aged 40–69 years^24^. We show heterogeneous associations of multiplexed environmental factors available in the UKB (i.e., air pollution, green and blue spaces, and noise) with multidimensional aging metrics while air pollution presents a relatively large contribution in most cases. Then, five subpopulations with distinct environmental exposure patterns are identified, exhibiting the aging inequality, and the air pollution subpopulation has the worst aging status.

## Results

### Population characteristics

**Fig. 2:**
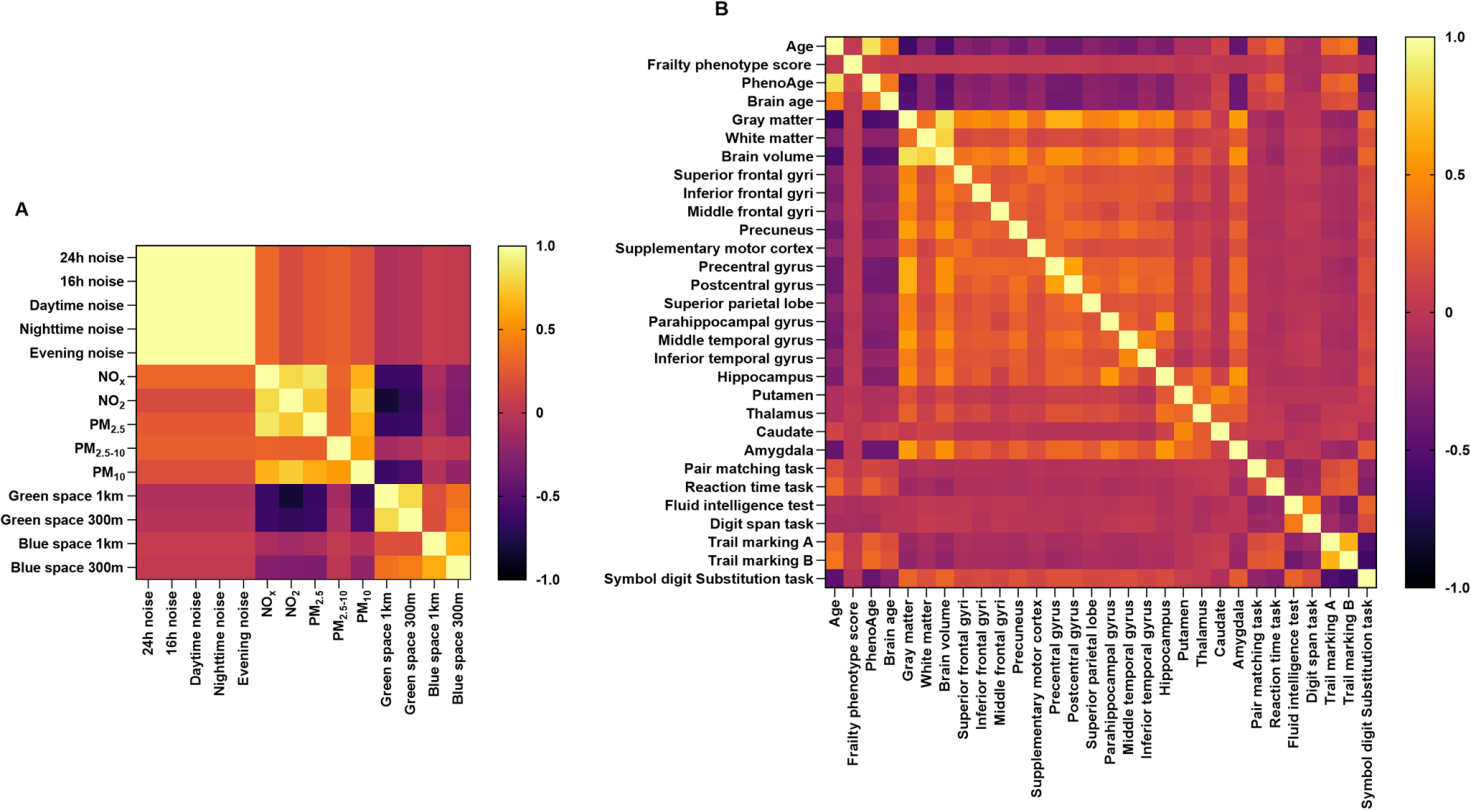
Heatmaps of Spearman’s correlations among multiplexed environmental factors. (**A**) and multidimensional aging metrics (**B**). PM_10_, particulate matter with aerodynamic diameter ≤10 µm; PM_2.5_, particulate matter with aerodynamic diameter ≤2.5 µm; PM_2.5–__10_, particulate matter with aerodynamic diameter between 2.5 and 10 µm; NO_2_, nitrogen dioxide; NO_*x*_, nitrogen oxides. We used Spearman’s correlations to assess the correlations among multiplexed environmental factors (**A**) and multidimensional aging metrics (**B**). Source data are provided as a Source Data file.

As shown in Supplementary Table 1, the number of participants was 344,088 and 416,998, respectively, in the analysis of PhenoAge and frailty phenotype score. A total of 34,588 participants, with a mean age of 55.46 (standard deviations = 7.37) years, were included in the analysis of brain age. Air pollution (except PM_2.5–__10_) was strongly positively correlated with each other (*r* > 0.60) (Fig. 2A). Green space and blue space in different buffers were strongly correlated with one another, while the correlation between green space (1000 m buffer) and blue space (1000 m buffer) was relatively weak (*r* = 0.18). However, not all aging metrics were highly correlated (Fig. 2B). Particularly, age showed a strong correlation with PhenoAge (*r* = 0.85), as well as the volume of gray matter (GM) (*r* = −0.61) and brain (*r* = −0.56) (Fig. 2B).

### Relative individual contributions of multiplexed environmental factors to aging

**Fig. 3:**
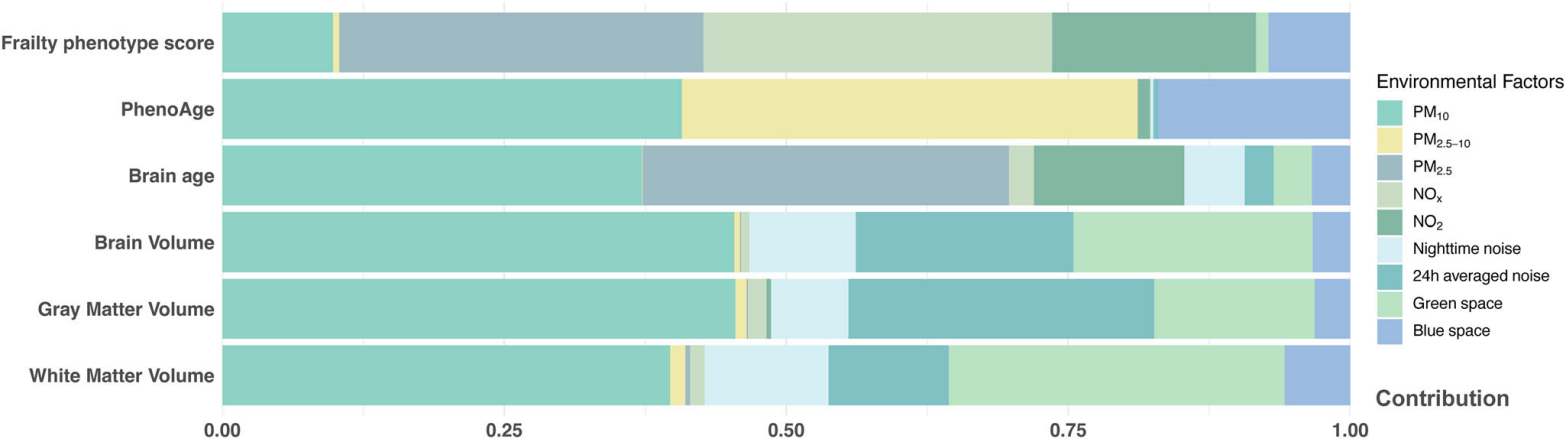
Relative individual contributions of multiplexed environmental factors to aging metrics. PM_10_, particulate matter with aerodynamic diameter ≤10 µm; PM_2.5_, particulate matter with aerodynamic diameter ≤2.5 µm; PM_2.5–__10_, particulate matter with aerodynamic diameter between 2.5 and 10 µm; NO_2_, nitrogen dioxide; NO_*x*_, nitrogen oxides. We used WQS to evaluate the relative individual contribution of multiplexed environmental factors to aging metrics. Source data are provided as a Source Data file.

**Fig. 4:**
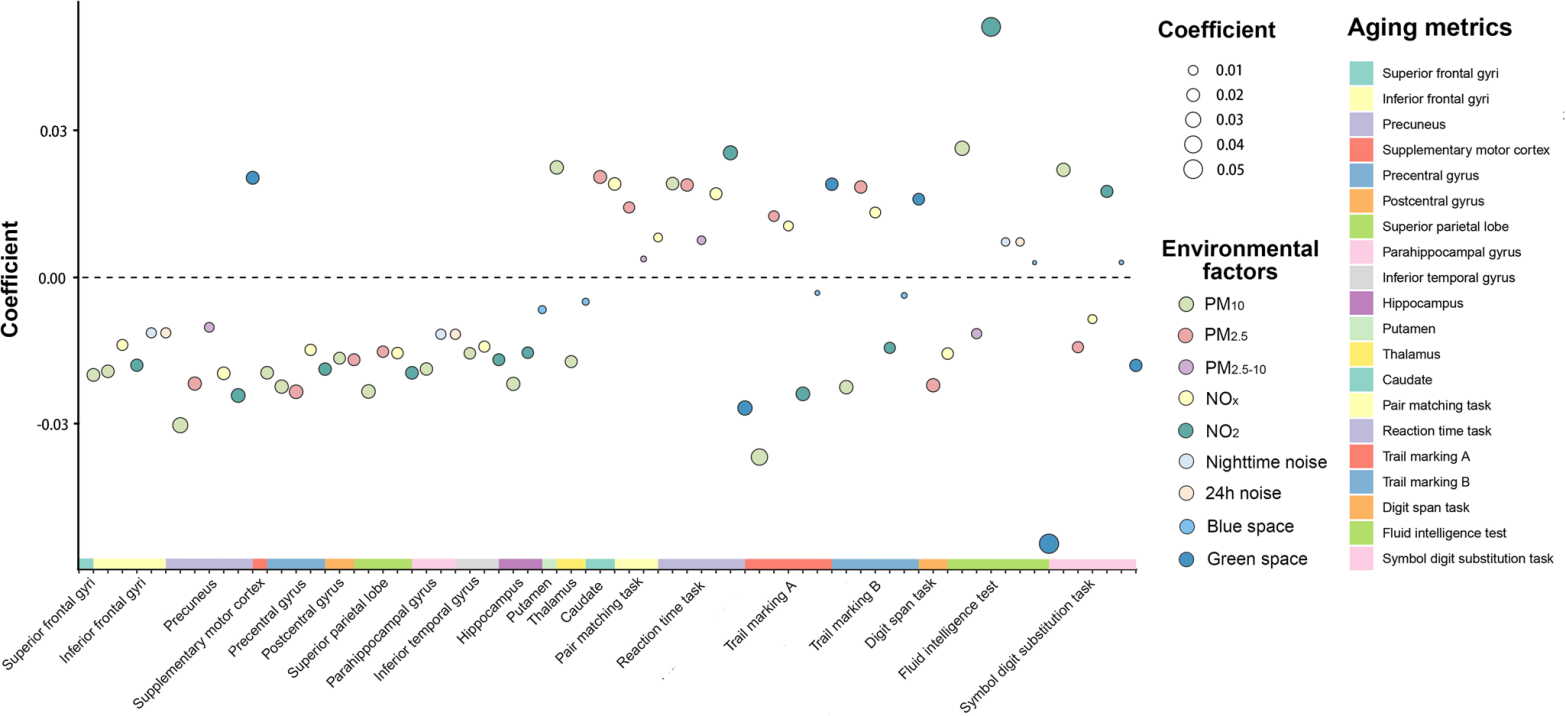
Associations of each environmental factor with volumes of aging-related regions and subcortical areas, and cognitive performances. We used linear regression to evaluate the association of each environmental factor with aging-related regions, subcortical areas, and cognitive performances. Benjamini–Hochberg procedure was used to control the family-wise error rate in the main analyses (*n* = 285). Only associations with an FDR < 0.05 were displayed. The height, color, and size of each data point indicate the coefficient (β) between each environmental factor and one aging metric. The horizontal dashed line denotes the positive and negative correlation boundary. Source data are provided as a Source Data file.

**Fig. 5:**
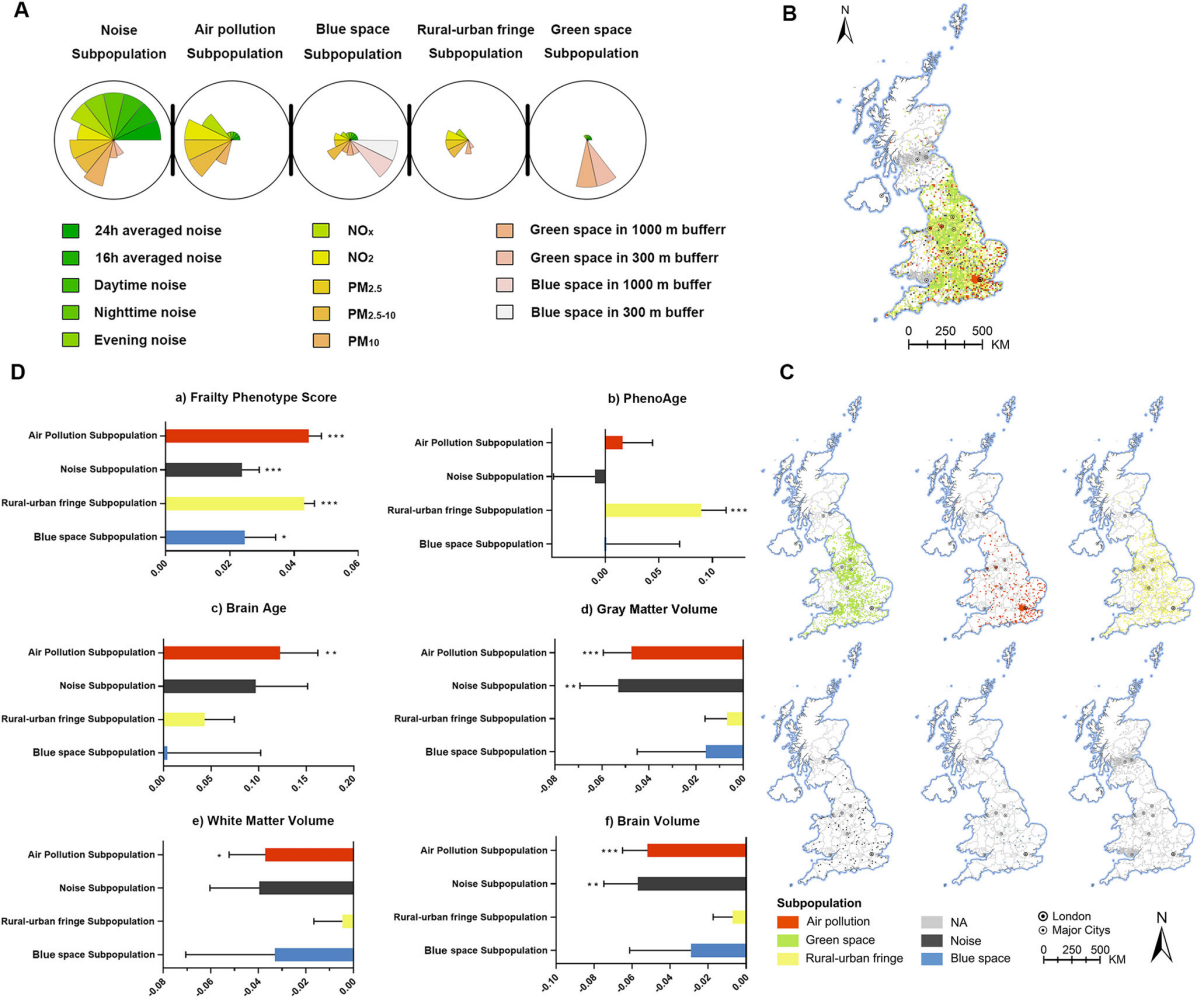
The characteristics (A) and distributions of identified subpopulations (B, C), and the associations of subpopulations with various aging metrics (D). SOM, self-organizing map. We used SOM analyses to recognize group structure. Populations were differentiated into air subpopulation (red, specific name was used only to refer to the main environmental exposure feature, not all features), green space subpopulation (green), rural–urban fringe subpopulation (yellow), noise subpopulation (black), blue space subpopulation (blue), and others (gray). **A** Characteristics of subpopulations. A larger sector size represents the larger amount of a specific environmental factor. **B** Distributions of subpopulations and **C** Distributions separately. We used multiple linear regression models to evaluate the associations of subpopulations with various aging metrics (**D**). Linear regression models were performed to examine the associations of subpopulations with various aging metrics. All models were adjusted for age, sex, ethnicity, neighborhood socioeconomic status (nSES), smoking status, BMI (category variable), alcohol intake frequency, regular exercise, healthy diet, history of cardiovascular disease (CVD), and cancer at baseline. Benjamini–Hochberg procedure was used to control the family-wise error rate in the main analyses (*n* = 285). Two-sided *P* value of <0.05 was considered statistically significant (values are represented as a coefficient ± standard error of the mean. *P < 0.05, **P < 0.01, ***P < 0.005; different subpopulations vs. green space subpopulation). The blank map of the UK can be freely downloaded from GADM version 4.1 (https://www.gadm.org/). Source data are provided as a Source Data file.

As shown in Figs. 3–5, multiplexed environmental factors were significantly associated with all aging metrics, and PM_10_ was almost always dominant. However, fair heterogeneities in the relative contributions of the same environmental factors to multidimensional aging metrics were observed.

Specifically, PhenoAge had a significant positive association with multiplexed environmental factors (*β* = 0.043; 95% CI: 0.014, 0.072, Fig. 3), and PM_10_ was predominant, with a relative contribution of 40.8%, as a major contributor among the multiplexed environmental factors to the variance in PhenoAge. Meanwhile, PM_2.5–__10_ (40.4%) indicated its second-largest contribution to the variance in PhenoAge. The relative contributions were similar to that for brain age (PM_10_ contributed 37.2% and PM_2.5_ contributed 32.5%).

Regarding frailty phenotype score, PM_2.5_ (32.3%) and NO_*x*_ (30.9%) surpassed PM_10_ (9.8%), taking the dominant place. Although 24-h average noise did not show much contribution to other aging metrics, it presented a considerable contribution to the variance in GM (27.1%) and brain volume (19.3%).

As shown in Fig. 4, air pollution (particularly PM_10_) still accounted for the major contributions of variance in these brain indicators and cognitive performance. Compared with other regions, the precuneus and postcentral gyrus were more influenced by multiplexed environmental factors. Green space showed considerable contributions to variance in cognitive performance (e.g., fluid intelligence test, digit span task, and trail making).

### Specific environmental exposure patterns among populations across the UK

Based on multiplexed environmental factors, self-organizing maps (SOMs) identified five subpopulations (Fig. 5A–C) illustrates the spatially explicit distribution of the five subpopulations in the UK. Participants were not evenly distributed across subpopulations, and we named subpopulations mainly based on each environmental exposure pattern and location characteristics. Most participants experienced moderate air pollution and held moderate green space and blue space, roughly circled the major cities as the transition from cities to suburbs and mountain areas, probably representing the population living in rural‒urban fringe areas (the rural‒urban fringe subpopulation). The air pollution subpopulation experienced the most serious air pollution and held the least green space, distributed more densely in the southeast UK and mostly located in major cities, such as London, Manchester, Liverpool, and Birmingham (dotted in Fig. 5B, C with circles), probably representing the population living in the center of major cities. The green space subpopulation experienced the lowest air pollution and held the greenest space, likely adjacent to mountain areas (e.g., the Peak District National Park, Mendip Hills, North Wessex Downs AONB, etc.), wrapping around the outside of the rural‒urban fringe subpopulation. The noise subpopulation and the blue space subpopulation were distributed in a relatively scattered manner with likely linear tendencies. The noise subpopulation experienced the most severe road traffic noise and seemed to be adjacent to the cross of major roads in relatively open suburbs (Supplementary Fig. 2)^25^. The blue space subpopulation had the most blue space and was probably distributed along rivers (Otter, Rother, Blyth) (Supplementary Fig. 3)^26^. In the multiple linear regression model, compared to the green space subpopulation, the blue space subpopulation showed a nonsignificant difference, except for frailty phenotype score (*β* = 0.025; 95% CI: 0.005, 0.045) (Fig. 5D). Specifically, the air pollution subpopulation mostly located in major cities consistently had a worse aging status; for example, they had the highest frailty phenotype score (*β* = 0.045; 95% CI: 0.037, 0.053), and the worst brain age (*β* = 0.133; 95% CI: 0.053, 0.213). Although the rural‒urban fringe subpopulation experienced lower air pollution and road traffic noise than the noise subpopulation, less green space among them may result in their worse frailty phenotype score as high as air pollution subpopulation (*β* = 0.045; 95% CI: 0.039, 0.051) and accelerated PhenoAge (*β* = 0.081; 95% CI: 0.036, 0.126). The noise subpopulation represented the second worst aging status in GM volume (*β* = −0.045; 95% CI: −0.069, −0.021) and the whole-brain volume (*β* = −0.052; 95% CI: −0.085, −0.019), which might imply possible interactive negative effect between air pollution and noise on brain aging. These results provided contribution estimates based on observed contrasts in the multiplexed environmental factors and suggested synergistic contributions of air pollution, road traffic noise, green space, and blue space.

### Additional analyses

The results of the linear regression models were largely consistent with those of WQS (Supplementary Table 2). For example, an interquartile range (IQR) increase in PM_10_ contributed the most to variance in PhenoAge (*β* = 0.032; 95% CI: 0.085, 0.056), while an IQR increase in PM_2.5_ contributed the most to variance in frailty phenotype score (*β* = 0.023; 95% CI: 0.019, 0.027). The findings mainly remained the same while adjusting for iSES, instead of nSES. Particular matters and nitrogen oxides continued to show the dominant contribution to multidimensional aging metrics in WQS models. The air pollution subpopulation had the worst aging status, e.g., they had the highest PhenoAge (*β* = 0.189; 95% CI: 0.134, 0.244). Detailed results were reported in Supplementary Results and Supplementary Tables 3‒6. After eliminating participants living in the current location for <5 years, the results still largely remained the same (Supplementary Tables 7‒8).

According to the stratified analyses (Supplementary Tables 9‒16), overall, air pollution remained the most important contributor to multidimensional aging metrics. Males seemed to be a vulnerable subpopulation in which each aging metric was significantly associated with multiplexed environmental factors. In males, PM_10_ made the dominant contribution to PhenoAge (45.5%), white matter (WM) volume (54.5%), and brain volume (52.7%). Meanwhile, heavy drinkers (drinking daily or almost daily) were more likely to be influenced by multiplexed environmental factors, especially for their brain volumes. For example, multiplexed environmental factors were significantly associated with the whole-brain volume among heavy drinkers (*β* = −3505.70; 95% CI: −5730.89, −1280.51) and moderate drinkers (drinking one to four times per week) (*β* = −2010.94; 95% CI: −3342.82, −678.98).

Compared to the green space subpopulation, the air pollution subpopulation remained to have the worst aging status in most aging metrics, especially for frailty phenotype score and the rural‒urban subpopulation also showed a worse aging status in stratified analyses. For example, among participants with age ≥60 years, both the air pollution subpopulation (*β* = 0.047; 95% CI: 0.035, 0.059) and the rural‒urban subpopulation (*β* = 0.042; 95% CI: 0.032, 0.052) had a higher frailty phenotype score.

## Discussion

Based on unique data from the UK, we disentangled the complex individual and joint contributions of the multiplexed environmental factors to aging, indicative of multidimensional aging metrics. The results suggested synergistic contributions of multiplexed environmental factors to aging, and the largest contributor was air pollution. Moreover, we also found heterogeneities in the relative contributions of environmental factors to multidimensional aging. Particulate matter (i.e., PM_2.5_, PM_2.5‒__10_, and PM_10_) showed the predominant contribution to variance in multidimensional aging metrics, while noise and green space showed considerable contribution to brain volumes. SOM reduced the dimensionality of the data and identified subpopulations exhibiting multiple environmental exposure patterns. Interestingly, the associations between identified subpopulations and multidimensional aging metrics were largely consistent. Compared to the green space subpopulation, the air pollution subpopulation had the worst aging status, indicative of almost all aging metrics (e.g., highest frailty phenotype score and brain age). The findings provide strong evidence of the joint contribution of multiplexed environmental factors to aging and heterogeneity in the contributions of the same environmental factors to multidimensional aging and serve as a proof-of-concept study for disentangling multifactor and multioutcome issues.

Despite many studies examining the associations of some individual environmental factors (e.g., PM_2.5_^21,27,28^, NO_2_^21^, and green space^29–32^) with aging, few have examined the relative contributions of multiple environmental factors to aging. Evaluating single individual factors’ contributions without considering other environmental factors may ignore potential confounding and interaction contributions, partially due to the strong correlations among the multiplexed environmental factors, as we observed in Fig. 2. For instance, higher levels of green space tend to be related to lower levels of air pollution and traffic noise^33^. The geographical relations between air pollution and road traffic noise may be universal because road traffic noise is connected to traffic intensity, which impacts traffic-associated air pollutants^33,34^. Consequently, conclusions solely evaluating individual factors may be limited and biased, which makes them difficult to be interpreted and validated. This study holistically considered multiplexed environmental factors and disentangled their complex associations with aging to some extent, which may serve as a window for predicting various health outcomes earlier and more accurately, as well as another angle when interpreting results from previous studies that only considered a single environmental factor.

Given the global disparity in environmental pollution^35,36^, individuals may have heterogeneous environmental exposure patterns^37^ and thus face differentiated health risks. In this study, SOM reduced the dimensionality of the data and identified five subpopulations across the UK. These subpopulations with distinct environmental exposure patterns showed significant aging disparities. Compared to the green space subpopulation, the air pollutant subpopulation mostly located in major cities in the UK had the worst aging status throughout various aging metrics, indicating the important contribution of air pollution to aging, which was consistent with the results from WQS. Interestingly, although the rural‒urban fringe subpopulation had lower air pollution and road traffic noise than the noise subpopulation, it still suffered from a poor aging status (e.g., the second highest frailty phenotype score and the highest PhenoAge), which may be due to less green space. Because the rural‒urban fringe subpopulation was mainly located between the air pollutant subpopulation and the green space subpopulation, it may imply that the poor environmental qualities in major cities could have a radiative effect on persons in surrounding areas, whose accelerated aging has probably been ignored previously. This study may serve as a conceptual framework for more accurately identifying subpopulations with the same environmental exposure pattern and locations, helping develop targeted policies to improve persons’ surrounding environmental qualities and further relieve the aging burden.

In addition to the oversimplified choice of multiplexed environmental factors, the metrics of aging were also not comprehensive in previous studies. As a multifaceted process with variation among individuals and organs^15^, aging is unlikely to be interpreted by a single-dimensional aging metric. Using various aging metrics, which focus on multisystemic and domain-specific dimensions, our study revealed heterogeneity in the relative contributions of the same environmental factors to multidimensional aging. Such heterogeneity further stressed the complexity of the aging process and the different biological mechanisms of how environmental factors may affect the aging process. First, recent studies have reported differentiated aging clocks and biomarkers involving various organs (e.g., liver and kidney) and systems (e.g., immune systems and metabolic system)^15,38,39^, which imply that there might be systemic aging drivers/clocks overlaid with organ/tissue-specific counterparts^38^, exhibiting complicated interactions. Second, taking road traffic noise as an example, we found that it showed more considerable contributions to the brain aging process (e.g., brain age acceleration and gray volumes decrease). When noise travels through the auditory pathway to the brain, it triggers the paraventricular nucleus of the hypothalamus to release corticotrophin-releasing hormone. This process leads to the activation of proinflammatory cytokines and oxidative stress, which in turn stimulate the synthesis, secretion, and neurotoxicity of neurotransmitters. Additionally, many studies have demonstrated that the brain’s susceptibility to noise can be attributed to the impact of stress on the higher cortical and limbic structures^40,41^. Besides, several genes (e.g., SOX2^42,43^, iNOS^44^, and NXN^45,46^) were significantly associated with exposure to specific air pollutants (e.g., PM_2.5_ and PM_10_), which may further influence various biological pathways (e.g., insulin resistance and TNF signaling pathway)^47^. Further, asynchronous inter- and intra-organ gene expression during aging process^39,48^ could be differentially affected by specific pathways. Future studies are poised to more systematically unravel the heterogeneous contributions of specific exposures to different aging pathways and molecules.

Overall, as a multisystemic process, aging is typically a multidimensional outcome that provides a window into disease prediction and tracing. Quantifying the contribution of environmental factors to multidimensional aging holds substantial promise for precision healthy aging and related environmental management. It should be noted that although this study used several classic aging metrics, as the development of new aging metrics of various organs and systems (recent advanced aging measurements), the present results may be biased to some extent.

To our knowledge, this was the first study to link multiplexed environmental factors to multidimensional aging, providing a proof-of-concept study for dealing with multifactor and multioutcome issues. The heterogeneity that we observed is actually a common dilemma when including multidimensional factors and outcomes simultaneously. The intriguing finding is that the results turn to consistency in the associations of subpopulations with multidimensional aging. The air pollution group remained at the worst aging status, indicative of multidimensional aging metrics, while the green space group remained the best. This implies that the heterogeneity diminished or, to some extent, was concealed when reducing the dimensionality of data on multiplexed environmental factors. In this way, we were able to focus on the latent inequality in the macro dimension, which made more sense in public health. We present efforts in dealing with multifactor and multioutcome issues, but more challenges remain. First, the concept of exposomes and phenomics is attractive. UKB provides a unique opportunity to analyze complex associations of multiple factors and outcomes, but we face the problem of having varying sample sizes for various outcomes. Moreover, few databases have such comprehensive data. Although several large cohorts, such as CHIMGEN^49^, ABCD^50^, cVEDA^51^, and Generation R^52^, provide relatively complete exposures or outcomes, it is still difficult to standardize and harmonize the data. This is one of the key reasons that the findings such as ours could not be verified externally. Some organizations, such as Gateway to Global Aging Data^53^, are making efforts, but it remains to be a long way. Second, although we used cutting-edge statistical methods and found some interesting results in this study, the increasing data dimensions and sample size will bring great statistical challenges. Whether other new methods, including network analysis^54^ and artificial intelligence, could address these challenges is unclear. Finally, many studies assume that exposure affects outcomes linearly, but this is not necessarily true. Whether multiple factors affect outcomes in a nonlinear or even systematic way requires further investigation. For example, Cohen et al. proposed understanding aging from the perspective of complex systems, and many aging processes are characterized by the interaction of multiple systems^15,54–56^.

This study has several strengths. First, the large sample size of the middle-aged and older adults from UKB and its spatial distribution across the UK allowed for higher precision and power than smaller studies, especially in multifactor and multioutcome analyses. The diversity of the UK also allowed the SOM to identify a complete collection of environmental exposure patterns. Second, cutting-edged statistical methods provide a more comprehensive interpretation of the individual and joint contributions of the multiplexed environmental factors to aging. Third, the actual distributions of subpopulations on the UK map further validated the reasonability and accuracy of the SOM clustering results. Fourth, using multidimensional aging metrics, we comprehensively depicted the whole landscape of aging and indicated the heterogeneity of the contribution of the same environmental factors to multidimensional aging, which highlights the necessity and challenges when dealing with multifactor and multioutcome issues.

However, there are still some limitations. First, as a cross-sectional study, the causal inference of this study was not as strong. To further verify our findings, effective methods to analyze the longitudinal contribution of baseline multiplexed environmental factors to subsequent health outcomes are urgently needed. Second, the data on more environmental exposures such as night light, indoor air pollution, and temperature, which may be also critical to aging, were not available in the UKB. Future studies are needed to depict more comprehensive individual environmental factors. Third, due to the assumed national traffic flow baseline value and UKB’s noise algorithm only considering major road noise and not considering other noise types (e.g., the impact of secondary roads), noise exposures for those at low exposure levels may be overestimated, and exposure for those living in areas with heavily trafficked minor roads may be underestimated. This may also partially explain the scattered distribution of the noise subpopulation in this study. Fourth, our study’s participants were mostly White, healthier, and had higher socioeconomic status (SES) than the general population in the UK. Finally, although the data possessed were consistent with recent research, the time duration between different data collection, especially between baseline data collection and imaging assessment, may cause potential confounding effects.

In this large sample of a UK population, we captured the relative contributions of multiplexed environmental factors to aging and revealed the heterogeneity in the same environmental factors to multidimensional aging. Moreover, we identified five subpopulations with different environmental exposure patterns across the UK and observed their differentiated distribution and associations with multidimensional aging, with the air pollution group having the worst aging status. This proof-of-concept study reveals how imperative it is to holistically consider multiplexed environmental factors in analyses of their associations with aging and further points out the necessity and challenges when dealing with multifactor and multioutcome issues.

## Methods

### Study population

UKB is a national cohort study conducted from 2006 to 2010 and recruited ~500,000 participants aged 40–69 years in the UK. The data on the baseline questionnaire and anthropometric measures were collected at 22 assessment centers across England, Wales, and Scotland. A detailed description of the sampling design and data quality of UKB was published elsewhere^24,57^. In the analysis of each aging metric, participants with missing data needed to calculate the aging metric were excluded. Details were provided in Supplementary Fig. 1. The North West Multi-Centre Research Ethics Committee as a Research Tissue Bank approved the UKB. Each participant provided written informed consent before the study, and researchers were allowed to use data from UKB without additional ethical clearance.

### Exposure assessment

#### Air pollution measurements

The land use regression model integrating multisource predictors such as road, land use, and topography, developed as part of the European Study of Cohorts for Air Pollution Effects project^58,59^, was adopted to estimate the annual average concentration of particulate matter with a diameter of 10 µm or less (PM_10_), particulate matter with a diameter of 2.5 and 10 µm (PM_2.5–10_), and particulate matter with a diameter of 2.5 µm or less (PM_2.5_), nitrogen dioxide (NO_2_), and nitrogen oxides (NO_*x*_) at a spatial resolution of 100 m. We used annual average air pollutant concentrations at participants’ residential locations collected at the baseline visit to assess individual exposures to air pollution^60,61^. Annual air pollution concentrations for NO_2_ and PM_10_ were available during 2005–2010 and 2007–2010, respectively, and the annual averages of NO_2_ and PM_10_ were used as exposures in the analysis. While PM_2.5_, PM_2.5–10_, and NO_*x*_ were only available for 2010; and thus, we used the 2010 data of these pollutants to assess individual exposures^62^.

#### Residential green and blue spaces

The percentages of green spaces and blue spaces in the participants’ home neighborhood (300 m and 1 km radius around residential location) were estimated based on a land use map from the 2005 Generalized Land Use Database (GLUD), provided by the UK Department for Communities and Local Government (https://www.gov.uk/government/statistics). The GLUD records the dominant land use types based on a ten-class typology (e.g., domestic buildings, nondomestic buildings, roads, green space, blue, etc.) at a spatial resolution of 1 km^63^. Each participant’s exposure to green spaces or blue spaces was computed by overlaying the mapped green and blue spaces with the circle buffers surrounding residential locations in the geographic information system software to calculate the percentage of each buffer that contained these land cover types. Considering that the residential green (and blue) spaces in the buffers with radii from 300 to 1000 m were highly correlated (Spearman’s correlation coefficient (ρs) = 0.813 for green spaces and 0.629 for blue spaces), we only reported the associations with the green (and blue) space exposures using a buffer with a radius of 1000 m.

#### Road traffic noise

The 2009 annual mean road traffic noise of all roads in the participant’s home neighborhood (500 m radius around residential location) was modeled using the Common Noise Assessment Methods, developed from the European Union noise modeling framework^64,65^. The model considered detailed information on absorption from buildings, noise propagation and the distance between receptor and source, land use and angle of view, building heights, meteorology, road network geography, and land cover when calculating hourly vehicle flows using a daily average traffic profile^19^ and was widely used in previous studies^19,66^. We used the 24 h averaged noise (weighted average 24 h noise sound level, with a penalty of 5 and 10 dB added to the evening hours and night hours, respectively) and night-time noise (average sound pressure level during night-time hours 23:00–07:00) to be comparable to previous studies^19^.

### Multidimensional aging metrics

#### A multisystemic aging metric—PhenoAge

Derived from multisystemic chemistry biomarkers, PhenoAge serves as a relatively comprehensive aging metric. The biomarkers obtained from blood samples at the time of participant enrollment were used to calculate PhenoAge^67^. Within 24 h of the blood draw, the samples were normally analyzed at the UKB central laboratory using Beckman Coulter LH750 instruments. The laboratory results were then recorded in the participant’s data files. The UKB website provides more details about biomarker data processing^68,69^. PhenoAge was developed by regressing mortality hazard on 42 clinical biomarkers and chronological age^7,8^, and has been widely used and demonstrated to capture morbidity and mortality risks across diverse subpopulations from various countries^48,70^.

Based on the Gompertz distribution, chronological age and nine clinical biomarkers were selected into a parametric proportional hazards model, and 10-year mortality risk was converted into units of years. The equation to calculate PhenoAge is presented as follows

$${{{{{\rm{Phenotypic}}}}}}\,{{{{{\rm{Age}}}}}}=141.50225+\frac{{{{{\mathrm{ln}}}}}\,(-0.00553\times \,{{{{\mathrm{ln}}}}}(1-{{{{{\rm{Mortality}}}}}}\,{{{{{\rm{Risk}}}}}}))}{0.090165}$$

where

$${{{{{\rm{Mortality}}}}}}\,{{{{{\rm{Risk}}}}}}=1-{{{{{{\rm{e}}}}}}}^{\frac{-{{{{{{\rm{e}}}}}}}^{{{{{{\rm{xb}}}}}}}(\exp (120\times \gamma )-1)}{\gamma }}$$

$$\gamma=0.0076927$$

$$xb=	 -19.907-0.0336\times {{{{{\rm{albumin}}}}}}+0.0095\times {{{{{\rm{creatinine}}}}}} \\ 	+0.1953\times {{{{{\rm{glucose}}}}}}+0.0954\times \,{{{{\mathrm{ln}}}}}({{{{{\rm{C}}}}}}-{{{{{\rm{reactive}}}}}}\,{{{{{\rm{protein}}}}}})\\ 	 -0.012\times {{{{{\rm{lymphocyte}}}}}}\,{{{{{\rm{percent}}}}}}+0.0268\times {{{{{\rm{mean}}}}}}\,{{{{{\rm{corpuscular}}}}}}\,{{{{{\rm{volume}}}}}} \\ 	+0.3306\times {{{{{\rm{red}}}}}}\,{{{{{\rm{cell}}}}}}\,{{{{{\rm{distribution}}}}}}\,{{{{{\rm{width}}}}}}+0.00188\times {{{{{\rm{alkaline}}}}}}\,{{{{{\rm{phosphatase}}}}}} \\ 	+0.0554\times {{{{{\rm{white}}}}}}\,{{{{{\rm{blood}}}}}}\,{{{{{\rm{cell}}}}}}\,{{{{{\rm{count}}}}}}+0.0804\times {{{{{\rm{chronological}}}}}}\,{{{{{\rm{age}}}}}}$$

#### Domain-specific aging metrics—physical functioning

Being widely used as a valid metric of the aging process in geriatrics and gerontology^71,72^, frailty is characterized by an increased vulnerability to stressor events caused by cumulative diminished reserve and dysregulation in multiple physiological systems^6^. We used frailty phenotype score, a widely used physical frailty measurement proposed by Fried et al.^6^ Frailty phenotype score was evaluated using five criteria (unintentional weight loss, exhaustion, weakness, slow gait speed, and low physical activity) and was used previously in the UKB^5^. Of the five criteria, weakness was assessed using objectively measured handgrip strength; the other four criteria were assessed using a self-report questionnaire (see details in the Supplementary Method). Frailty phenotype score ranged from 0 to 5, with a higher score indicating more severe frailty^5,6^.

#### Domain-specific aging metrics—brain

##### Brain volumes

Full details on the UKB neuroimaging data are provided here: https://biobank.ctsu.ox.ac.uk/crystal/crystal/docs/brain_mri.pdf. In brief, T1-weighted MRI used an MPRAGE sequence with 1-mm isotropic resolution. Brain volumes in mm^3^ were extracted from T1-structural brain MRI images, which were provided by ongoing research that started in 2014, to acquire high-quality imaging data from 100,000 predominantly healthy participants in the UKB Study^4^. In this study, we used imaging-derived phenotypes (IDPs) according to a previous study, in which the brain MRI processing pipelines were described in detail^73^. In total, 19 aging-related IDPs were involved, including GM volume, WM volume, brain volume (GM + WM), regional GM volumes (that is, volumes of GM in the superior frontal gyrus, inferior frontal gyrus, middle frontal gyrus, supplementary motor cortex, precentral gyrus, postcentral gyrus, precuneus, superior parietal lobe, parahippocampal gyrus, middle temporal gyrus, and inferior temporal gyrus), and volumes of several subcortical areas (including the hippocampus, putamen, thalamus, caudate, and amygdala)^74^. In particular, GM, WM, and total brain volumes were normalized by head size^75^. For the other IDPs, the sum of the volumes in the left and right hemispheres was calculated.

##### Brain age

Brain age is a widely used index for quantifying individuals’ brain health as deviation from a normative brain aging trajectory. Higher-than-expected brain age is thought to partially reflect the above-average rate of brain aging^10,76^. In general, based on a set of regional and global features extracted from T1w sequences (including 365 structural magnetic resonance imaging features partitioned into 68 features of cortical thickness, area, and gray‒white matter contrast, 66 features of cortical volume, 41 features of subcortical intensity, and 54 features of subcortical volume), the individuals’ brain age was estimated using machine learning method, gradient tree boosting as implemented in XGBoost (https://xgboost.readthedocs.io) and optimized using tenfold cross-validation and a randomized hyperparameter search^76^. The volumes were converted to *Z* scores.

### Cognitive performance

Cognition, as an important function of the human brain, was proven to be associated with the brain aging process^15^. In this study, we considered cognitive factors as an objective measurement of the whole-brain aging process. Thus, we obtained performance measures on seven cognitive tasks from the UKB and processed them as previously described^77,78^. Details of seven measures and calculations for analysis have been provided elsewhere^78^. The scores were first normalized, if not normally distributed, and then converted to *Z* scores.

The measurement time and number of participants for each exposure variable included in this study were provided in Supplementary Table 17.

### Covariates

Covariates were selected based on previous related studies^79,80^, including age, sex, ethnicity, neighborhood SES (nSES), smoking status, body mass index (BMI), alcohol intake frequency, regular exercise, healthy diet, history of cancer and cardiovascular disease (CVD) at baseline. The definition of covariates was reported in the Supporting Methods.

### Statistical analyses

Baseline characteristics of each group included in multidimensional aging metrics were described. Means (standard deviations) and numbers (percentages) were used to describe the continuous and categorical variables. Mann‒Whitney *U* and chi-squared tests were used to examine the differences in continuous and categorical variables. A two-sided *P* value of <0.05 was considered statistically significant in this study, unless otherwise stated.

### Individual contributions

We used WQS to estimate the relative individual contributions of the multiplexed environmental factors to aging^81^. All analyses were adjusted for age, sex, ethnicity, nSES, smoking status, BMI, alcohol intake frequency, regular exercise, healthy diet, and history of cancer and CVD at baseline. WQS is a widely used exposure-index method in epidemiological studies to address high dimensionality and collinearity^82^. Compared to other shrinkage models, WQS has better specificity and sensitivity^83^. Using a weighted average of factors in quantiles, WQS derives an index and then estimates the overall index effect and the weights of the deriving index by fitting a linear model between the outcome and index^84^. Because WQS assumes that all components are constrained to have the same direction of association (positive or inverse) with the outcome, positive and negative models were used for probable miscarriage outcomes^81^. All weights are required to add up to 1, and the empirical weight of each anion, which ranges from 0 to 1, indicates the individual contribution to the WQS index^81^. For the volume of specific brain regions and cognitive performance, we used linear regression to preliminarily examine their association with multiplexed environmental factors. The false discovery rate was controlled at 5% across all linear regression models in the main analyses (*n* = 285) using the Benjamini–Hochberg procedure (R package “Stats”, version 4.4.0).

Considering that the overall contribution of multiplexed environmental factors may not be instructive if factors act in different directions^85^ (i.e., one exposure has a protective contribution to the outcome, while another exposure has a harmful contribution), we reversed the green space and blue space exposure in the WQS regression model, with larger values representing less green space. During the model fitting process, the dataset was automatically divided into a 40% training set and a 60% validation set (R package “gWQS”, version 4.4.0). The training set was utilized for weight estimation, while the validation set was used to test the significance of the WQS index. The final WQS index of this study was averaged from the weights in the 500 bootstrap samples^86^.

### Joint contributions

SOMs are unsupervised methods that group observations with comparable exposure profiles to derive low-dimensional projections of class profiles (i.e., subpopulations). In this study, SOM was used to identify subpopulations with the same environmental exposure characteristics^87,88^, representing the homogenous and heterogeneous environmental exposure patterns among the population. SOM identified the best number of subpopulations by recognizing group structure using the within-subpopulation sum of squares and between-subpopulation sum of squares statistics^87^. In this study, aiming to capture general environmental exposure patterns in a large dimension, we performed SOM in participants with complete data on environmental factors.

Each subpopulation was then matched to the geographic map of the UK based on data from the participants’ addresses. Subpopulation’s multidimensional aging metrics were compared (vs. the green space subpopulation) using multiple linear regression models, adjusting for all covariates.

### Additional analysis

General linear regression models were performed as a robust test for the associations of multiplexed environmental factors with multidimensional aging metrics in WQS. We presented the linear effect estimates per an IQR increase in exposure to air pollution, green space, blue space, and road traffic noise by separately including one factor in the models. Given the participant’s mobility, which may bias the home location-based measurement of environmental exposure to some extent, we repeated the main analysis after eliminating participants living in the current location for <5 years. Moreover, considering the vital effect of SES on environmental inequality and the complexity of SES indicators (individual SES and nSES), we repeated the main analyses by replacing nSES with iSES to further examine the results. An overall SES variable was created by latent class analysis based on the abovementioned three individual socioeconomic factors (household income, education level, and employment status). According to the item-response probabilities, three latent classes were identified, representing high, medium, and low SES^79^ (details are reported in the “Methods”). Considering the potential heterogeneity among different subpopulations, we performed stratified analyses by sex, age, smoking status, and alcohol drink frequency.

### Reporting summary

Further information on research design is available in the Nature Portfolio Reporting Summary linked to this article.

### Supplementary information

Supplementary Information

Reporting Summary

Peer Review File

### Source data

Source data

## Data Availability

The data used in the present study are available from UKB with restrictions applied. Data were used under license and are thus not publicly available. Access to the UKB data can be requested through a standard protocol (https://www.ukbiobank.ac.uk/register-apply/). The use of UK Biobank data was performed under application 61856. Source data are provided with this paper.

## Code availability

All analyses were performed in R version 4.2.1 (http://www.R-project.org). The code used for WQS and SOM analyses is an adaptation of the R package “gWQS” and “kohonen” (version 3.0.12, https://github.com/blewy/Self_Organizing_maps_R), respectively, and has been made available through the GitHub repository: https://github.com/SiriusPu/Heterogeneous-associations-of-multiplexed-environmental-factors-and-multidimensional-aging-metrics. The code to calculate the brain age is available at https://github.com/LCBC-UiO/VidalPineiro_BrainAge.
