# Supplementary material for: Heterogeneous associations of multiplexed environmental factors and multidimensional aging metrics
Source: Nat Commun. 2024 Jun 10;15:4921. doi: 10.1038/s41467-024-49283-0 (PMC11164970; doi:10.1038/s41467-024-49283-0)
Supplement: Supplementary file 1 — Supplementary Information [file 41467_2024_49283_MOESM1_ESM.pdf]

----Supplementary Information for

Heterogeneous associations of multiplexed environmental factors and multidimensional aging metrics

Supplementary Information Text

Supplementary Method

Supplementary Results

Supplementary Table 1. Basic characteristics of participants included in analyses of various aging metrics.

Supplementary Table 2. The associations of multiplexed environmental factors with multidimensional aging metrics using linear regression models.

Supplementary Table 3. Contributions of multiplexed environmental factors to multidimensional aging metrics while adjusting for iSES.

Supplementary Table 4. Contributions of multiplexed environmental factors to volumes of regional GM volumes and subcortical areas while adjusting for iSES.

Supplementary Table 5. Multiplexed environmental factors' individual effect on cognitive performances using linear regression models while adjusting for iSES.

Supplementary Table 6. Associations of subpopulations with multidimensional aging metrics using linear regression models while adjusting for iSES.

Supplementary Table 7. Contributions of environmental factors to multidimensional aging metrics in subpopulations that have lived at current address for no less than five years.

Supplementary Table 8. Contributions of multiplexed environmental factors to multidimensional aging metrics in subpopulations that have lived at current address for no less than five years.

Supplementary Table 9. Subgroup analyses of contributions of environmental factors to multidimensional aging metrics stratified by sex.

Supplementary Table 10. Subgroup analyses of contributions of environmental

factors to multidimensional aging metrics stratified by age.

Supplementary Table 11. Subgroup analyses of contributions of environmental factors to multidimensional aging metrics stratified by smoking status.

Supplementary Table 12. Subgroup analyses of contributions of environmental factors to multidimensional aging metrics stratified by alcohol intake frequency.

Supplementary Table 13. Subgroup analyses of associations of subpopulations with multidimensional aging metrics using linear regression models stratified by sex.

Supplementary Table 14. Subgroup analyses of associations of subpopulations with multidimensional aging metrics using linear regression models stratified by age.

Supplementary Table 15. Subgroup analyses of associations of subpopulations with multidimensional aging metrics using linear regression models stratified by smoking status.

Supplementary Table 16. Subgroup analyses of associations of subpopulations with multidimensional aging metrics using linear regression models stratified by alcohol intake frequency.

Supplementary Table 17. Dictionary ID of variates.

Supplementary Figure 1. Flow chart of analytic sample from the UK Biobank

Supplementary Figure 2. Major roads in England 2017

Supplementary Figure 3. OS MasterMap rivers across England, Scotland and Wales

Supplementary Method

Definitions of Covariates

Townsend deprivation index was used as a neighborhood socioeconomic status (nSES) variable and was derived from national census data according to postcodes of residence, which considered car ownership, household overcrowding, owner occupation, and unemployment. Quartiles were calculated from the index, and the lowest quartile represents the most advantaged and the highest the least advantaged[1,2]. Trained nurses measured height and weight during the baseline assessment center visit, and body mass index (BMI) was calculated by dividing the weight in kilograms by the square of the height in metres. Alcohol intake frequency was based on self-reported frequency of alcohol intake (never or special occasions only; one to three times per month; one to four times per week; or daily or almost daily)[3]. Regular exercise was defined as meeting the current global health recommendations for physical activity (150 minutes of moderate activity or 75 minutes of vigorous activity or an equivalent combination), which equated to ≥500 Metabolic Equivalent of Task (MET)-minutes/week, or no regular exercise (<500 MET-minutes/week)[4]. A healthy diet was defined as a person who has not achieved the intake goals for more than half of the following components: fruits, vegetables, fish and shellfish, dairy products, whole grains, vegetable oils, refined grains, sugar-sweetened beverages, and unprocessed meats[5]. The details of the intake goals of each dietary component have been published elsewhere[5,6]. The history of cancer and cardiovascular disease (CVD) at baseline was based on self-reports or medical records.

Assessment of individual socioeconomic status using latent class analysis

We used three variables (education level, occupation, and family income level) to generate an overall individual socioeconomic status (iSES) variable in the UKB. There were seven levels for education level: 1) None of the above ((equivalent to less than a high school diploma); 2) Other professional qualifications e.g.: nursing, teaching; 3) National Vocational Qualification or Higher National Diploma or A

Higher National Certificate or equivalent; 4) CSEs or equivalent; 5) O levels/GCSEs or equivalent; 6) A levels/AS levels or equivalent and 7) College or University degree. There were two levels for occupation: 1) employed (including those in paid employment or self-employed, retired, doing unpaid or voluntary work, or being full or part-time students) and 2) unemployed. There were five levels for family income level: <£18,000 ($25,000; €21,000), £18,000-£30,999, £31,000-£51,999, £52,000-£100,000, >£100,000 (participants who reported "do not know" or "prefer not to answer" were excluded from the main analyses as missing values). We did not consider health insurance as a part of iSES since the National Health Service, a publicly financed healthcare system intended to offer comprehensive, universal, and free treatments was established in the UK[7]. We conducted latent class analyses of different numbers of latent classes to select a reasonable model. We set the maximum absolute deviation to <0.000001 between the parameter estimates of two successive iterations, which meant iteration would stop if the difference between the parameter estimates was less than <0.000001 in two successive iterations. Bayesian information criterion (BIC), Akaike information criterion (AIC), likelihood ratio statistic G^2, and likelihood ratio statistic χ^2 were used for the model selection. We also used the mean posterior probability for the model selection, which indicated the uncertainty of posterior classification (the higher the mean posterior probability, the lower the posterior classification) and a value of 0.7 or more reflected an acceptable uncertainty.

Because models with six latent classes or more failed to converge (lack of degree of freedom), we only presented information on models with five or fewer latent classes. The following Figures present that G2 statistics, χ2 statistics, BIC, and AIC continued to go down as the number of latent classes increased.

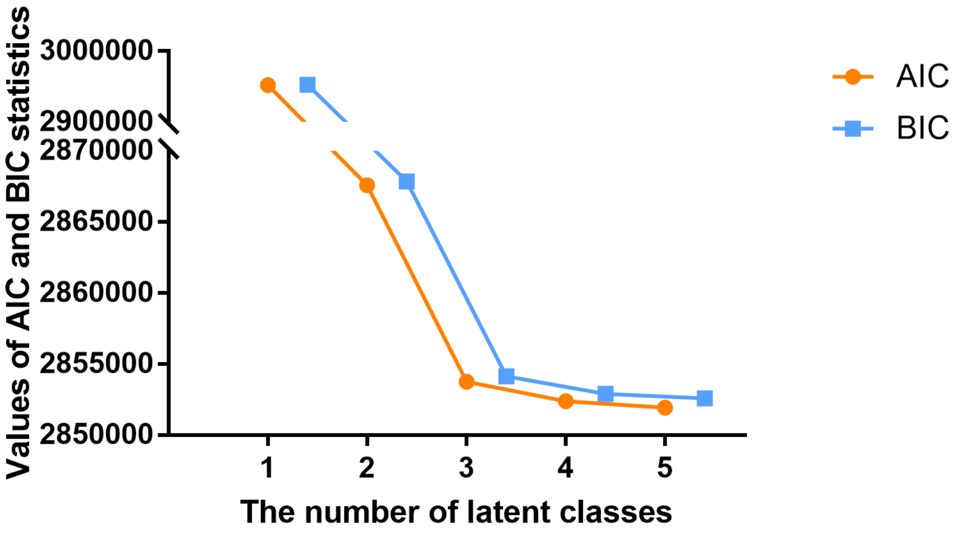

Figure for Supplementary Method. Values of AIC and BIC statistics according to the number of latent classes.

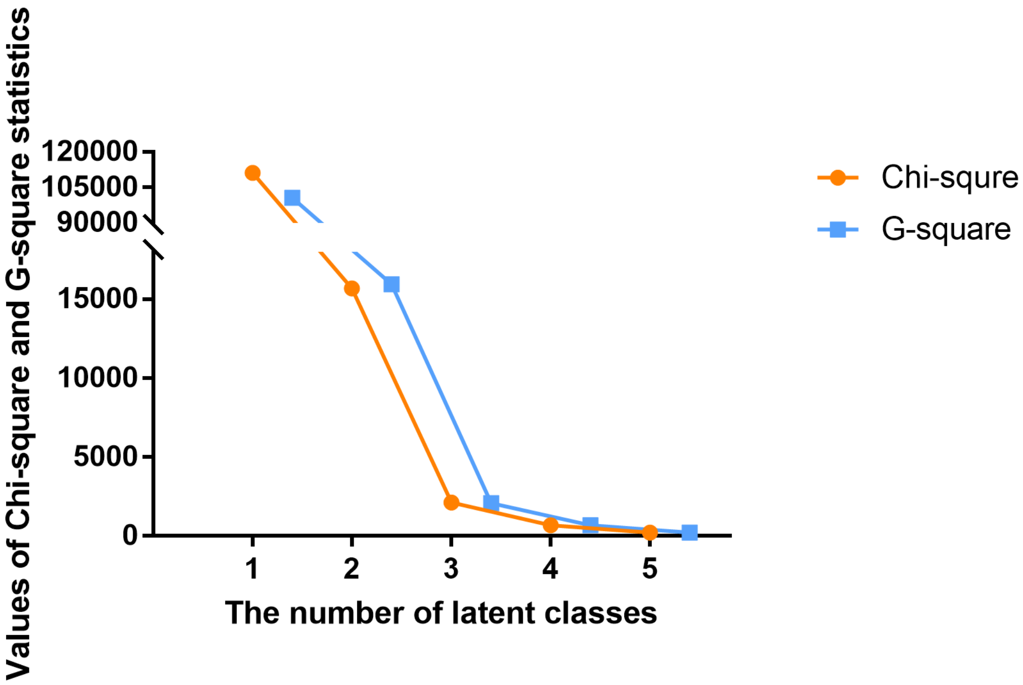

Figure for Supplementary Method. Values of Chi-square and G-square statistics according to the number of latent classes.

We examined the mean posterior probabilities for the model selection. The following table presents the mean posterior probabilities, item-response probabilities in models, and the prevalence of latent classes of three to five latent classes. All mean posterior

probabilities from the three-latent-class solution were ≥ 0.77; the mean posterior probability of latent class 2 from the four-latent-class solution was 0.69, which was less than 0.70; and the mean posterior probabilities of latent classes 1 and 5 from the five-latent-class solution were respectively 0.64 and 0.62, which were less than 0.70. Thus, the three-latent-class solution was the best in terms of the uncertainty of the posterior classification.

Table for Supplementary Method. Mean posterior probabilities, item-response probabilities in models, and the prevalence of latent classes of three to five latent classes in the UK Biobank.

Item	Latent class 1	Latent class 2	Latent class 3	Latent class 4	Latent class 5
		Three-latent-class solution			
Mean PP	0.92	0.77	0.80	NA	NA
Prevalence	0.20	0.52	0.28	NA	NA
Education 1	0.00	0.05	0.45	NA	NA
Education 2	0.01	0.07	0.04	NA	NA
Education 3	0.01	0.09	0.07	NA	NA
Education 4	0.00	0.07	0.05	NA	NA
Education 5	0.02	0.30	0.18	NA	NA
Education 6	0.17	0.12	0.07	NA	NA
Education 7	0.79	0.31	0.13	NA	NA
Income 1	0.00	0.00	0.81	NA	NA
Income 2	0.00	0.39	0.18	NA	NA
Income 3	0.01	0.49	0.00	NA	NA
Income 4	0.71	0.12	0.00	NA	NA
Income 5	0.27	0.00	0.00	NA	NA
Occupation 1	0.95	0.99	0.81	NA	NA
Occupation 2	0.05	0.01	0.19	NA	NA

Four-latent-class solution					
Mean PP	0.73	0.69	0.72	0.91	NA
Prevalence	0.37	0.12	0.21	0.30	NA
Education 1	0.00	0.51	0.40	0.00	NA
Education 2	0.03	0.21	0.01	0.05	NA
Education 3	0.00	0.29	0.02	0.09	NA
Education 4	0.00	0.00	0.07	0.13	NA
Education 5	0.01	0.00	0.24	0.52	NA
Education 6	0.09	0.00	0.09	0.21	NA
Education 7	0.87	0.00	0.16	0.00	NA
Income 1	0.00	0.21	0.94	0.00	NA
Income 2	0.18	0.60	0.05	0.35	NA
Income 3	0.27	0.15	0.01	0.48	NA
Income 4	0.40	0.05	0.00	0.17	NA
Income 5	0.14	0.00	0.01	0.00	NA
Occupation 1	0.97	1.00	0.77	0.98	NA
Occupation 2	0.03	0.00	0.23	0.02	NA
Five-latent-class solution					
Mean PP	0.64	0.72	0.93	0.70	0.62
Prevalence	0.07	0.09	0.33	0.35	0.16
Education 1	0.01	0.19	0.00	0.00	0.83
Education 2	0.01	0.02	0.09	0.03	0.07
Education 3	0.00	0.04	0.14	0.00	0.10
Education 4	0.01	0.16	0.12	0.00	0.00
Education 5	0.08	0.55	0.47	0.00	0.00
Education 6	0.27	0.03	0.19	0.09	0.00
Education 7	0.62	0.00	0.00	0.88	0.00
Income 1	0.69	0.89	0.00	0.00	0.60
Income 2	0.05	0.09	0.42	0.18	0.25

Income 3	0.06	0.01	0.43	0.28	0.12
Income 4	0.08	0.01	0.15	0.41	0.04
Income 5	0.12	0.00	0.00	0.13	0.00
Occupation 1	0.69	0.55	0.99	1.00	1.00
Occupation 2	0.31	0.45	0.01	0.00	0.00

Note: Mean PP, mean posterior probability; NA=not available.
Prevalence indicated the prevalence of each latent class.

Table for Supporting Method. Assessment of the five criteria for the frailty phenotype in the UKB.

Name	Criteria description	Categories
Weight loss	Self-reported: "Compared with one year ago, has your weight changed?"	1: Yes, lost weight; 0: Others
Exhaustion	Self-reported: "Over the past two weeks, how often have you felt tired or had little energy?"	1: More than half the days or nearly every day; 0: Others
Walking speed	Self-reported: "How would you describe your usual walking pace?"	1: Slow; 0: Others
Grip strength	Hand grip strength (left), kg;<br>Hand grip strength (right), kg	1: Maximal value of handgrip strength is less than or equal to sex and body mass index (BMI) adjusted cutoffs taken from Fried and colleagues[3];<br>Men:≤29 kg for BMI ≤24;≤30 kg for BMI 24.1–26;≤30 kg for BMI 26.1–28; or≤32 kg for BMI >28; Women:≤17 kg for BMI ≤23; ≤17.3 kg for BMI 23.1–26;≤18 kg for BMI 26.1–29;or≤21 kg for BMI>29<br>0: Others
Physical activity	Types of physical activity in last 4 weeks;<br>Frequency of heavy DIY/light DIY/other exercises/walking/strenuous sports in last 4 weeks	1: None or light activity with a frequency of once per week or less;<br>0: medium or heavy activity, or light activity more than once per week

Note: Criteria were adapted from Fried and colleagues[3]. Approximation based on available variables in UKB.

Supplementary Results

As shown in Supplementary Table 10-14, the results mostly remained the same while adjusting for iSES. The multiplexed environmental factors were significantly associated with all aging metrics. Air pollution still contributed most to variance in multidimensional aging metrics in most cases. Specifically, PhenoAge had a significant positive association with multiplexed environmental factors ($\beta = 0.043$; 95% CI: 0.039, 0.047, Supplementary Table 10), and PM_{10} was predominant, with a relative contribution of 67.7%, as a major contributor among the multiplexed environmental factors to the variance in PhenoAge. Meanwhile, Greenspace (13.3%) took the second-largest contribution to the variance in PhenoAge, whereas NO_2 ranked in third place with a relative contribution of 10.8%. Regarding the frailty phenotype score, NO_2 (50.8%) and NO_x (19.2%) surpassed PM_{10} (14.6%), taking the dominant place.

Compared to the green space subpopulation, the blue space subpopulation mostly showed a nonsignificant difference, except for the frailty phenotype score ($\beta = 0.050$; 95% CI: 0.030, 0.070), while all other subpopulations exhibited worse aging status (Supplementary Table 19). For example, the air pollution subpopulation still had the worst aging status; e.g., they had the highest PhenoAge ($\beta = 0.181$; 95% CI: 0.124, 0.238), and the rural–urban fringe subpopulation had the second worst aging status ($\beta = 0.104$; 95% CI: 0.057, 0.151).

Supplementary Table 1. Basic characteristics of participants included in analyses of various aging metrics.

Characteristics	Frailty Phenotype Score	PhenoAge	IDPs	Brain Age	Pairs Matching	Reaction Time	Trail Making A	Trail Making B	Digit Span	Fluid Intelligence	Symbol Digit Substitution
	416,998	344,088	38,423	34,588	413,438	413,212	88,559	88,557	46,933	151,137	100,387
**Age, mean (SD), year**	56.71 (7.90)	56.72 (7.89)	55.20 (7.41)	55.46 (7.37)	56.72 (7.90)	56.70 (7.90)	56.15 (7.51)	56.15 (7.51)	56.89 (7.90)	56.94 (7.87)	56.37 (7.53)
**Males (N, %)**	189,973 (45.6)	159,057 (46.2)	18,170 (47.3)	16,364 (47.3)	188,349 (45.6)	188,184 (45.5)	39,782 (44.9)	39,780 (44.9)	21,393 (45.6)	68,611 (45.4)	44,233 (44.1)
**Body mass index (kg/m^2)**											
**<18.5**	4,154 (1.0)	2,917 (0.8)	216 (0.6)	189 (0.5)	4,078 (1.0)	4,019 (1.0)	702 (0.8)	702 (0.8)	438 (0.9)	1,666 (1.1)	809 (0.8)
**≥18.5 and <25**	136,173 (32.7)	112,396 (32.7)	14,920 (38.8)	13,379 (38.7)	134,985 (32.6)	135,200 (32.7)	34,079 (38.5)	34,078 (38.5)	15,361 (32.7)	49,661 (32.9)	38,667 (38.5)
**≥25 and <30**	176,728 (42.4)	146,517 (42.6)	16,433 (42.8)	14,765 (42.7)	175,289 (42.4)	175,157 (42.4)	36,475 (41.2)	36,474 (41.2)	19,677 (41.9)	63,431 (42.0)	41,307 (41.1)
**≥30**	99,943 (24.0)	82,258 (23.9)	6,854 (17.8)	6,255 (18.1)	99,086 (24.0)	98,836 (23.9)	17,303 (19.5)	17,303 (19.5)	11,457 (24.4)	36,379 (24.1)	19,604 (19.5)
**Smoking status (N, %)**											
**Never smoker**	228,392 (54.8)	188,120 (54.7)	23,296 (60.6)	20,883 (60.5)	226,532 (54.8)	226,121 (54.7)	50,406 (56.9)	50,404 (56.9)	25,791 (55.0)	83,386 (55.2)	56,969 (56.7)
**Ever smoker**	146,081 (35.0)	120,951 (35.2)	12,769 (33.2)	11,570 (33.3)	144,880 (35.0)	145,068 (35.1)	31,948 (36.1)	31,948 (36.1)	16,482 (35.1)	53,041 (35.1)	36,408 (36.3)
**Current smoker**	42,525 (10.2)	35,017 (10.2)	2,358 (6.1)	2,135 (6.2)	42,026 (10.2)	42,023 (10.2)	6,205 (7.0)	6,205 (7.0)	4,660 (9.9)	14,710 (9.7)	7,010 (7.0)
**Alcohol intake frequency (%)**											

**Never or special occasions only**	80,366 (19.3)	65,576 (19.1)	4,862 (12.7)	4,405 (12.7)	79,647 (19.3)	78,648 (19.0)	12,932 (14.6)	12,932 (14.6)	9,111 (19.4)	29,876 (19.8)	14,910 (14.9)
**One to three times per month**	46,186 (11.1)	37,967 (11.0)	4,150 (10.8)	3,722 (10.8)	45,788 (11.1)	45,855 (11.1)	9,441 (10.7)	9,441 (10.7)	5,338 (11.4)	17,196 (11.4)	10,691 (10.6)
**One to four times per week**	203,932 (48.9)	168,699 (49.0)	20,688 (53.8)	18,539 (53.6)	202,204 (48.9)	202,618 (49.0)	44,844 (50.6)	44,843 (50.6)	22,934 (48.9)	72,527 (48.0)	50,672 (50.5)
**Daily or almost daily**	86,514 (20.7)	71,846 (20.9)	8,723 (22.7)	7,922 (22.9)	85,799 (20.8)	86,091 (20.8)	21,342 (24.1)	21,341 (24.1)	9,550 (20.3)	31,538 (20.9)	24,114 (24.0)
**Healthy diet, yes (N, %)**	334,977 (80.3)	276,348 (80.3)	31,423 (81.8)	28,311 (81.9)	332,212 (80.4)	331,955 (80.3)	73,135 (82.6)	73,135 (82.6)	38,294 (81.6)	121,648 (80.5)	83,179 (82.9)
**Ethnic group (N, %)**											
**White**	394,019 (94.5)	325,661 (94.6)	37,280 (97.0)	33,558 (97.0)	390,611 (94.5)	391,460 (94.7)	86,083 (97.2)	86,081 (97.2)	44,574 (95.0)	140,149 (92.7)	97,450 (97.1)
**Mixed**	2,491 (0.6)	2,042 (0.6)	173 (0.5)	158 (0.5)	2,465 (0.6)	2,454 (0.6)	498 (0.6)	498 (0.6)	206 (0.4)	1,100 (0.7)	563 (0.6)
**South Asian**	8,352 (2.0)	6,663 (1.9)	400 (1.0)	363 (1.0)	8,295 (2.0)	7,795 (1.9)	746 (0.8)	746 (0.8)	1,338 (2.9)	4,161 (2.8)	894 (0.9)
**Black**	7,015 (1.7)	5,577 (1.6)	264 (0.7)	233 (0.7)	6,994 (1.7)	6,628 (1.6)	536 (0.6)	536 (0.6)	376 (0.8)	3,548 (2.3)	655 (0.7)
**Chinese**	1,306 (0.3)	1,068 (0.3)	108 (0.3)	95 (0.3)	1,294 (0.3)	1,249 (0.3)	199 (0.2)	199 (0.2)	104 (0.2)	533 (0.4)	238 (0.2)
**Others**	3,815 (0.9)	3,077 (0.9)	198 (0.5)	181 (0.5)	3,779 (0.9)	3,626 (0.9)	497 (0.6)	497 (0.6)	335 (0.7)	1,646 (1.1)	587 (0.6)
**Neighborhood socioeconomic status (N, %)**											
**Quartile 1**	103,142 (24.7)	84,200 (24.5)	7,102 (18.5)	6,411 (18.5)	102,092 (24.7)	101,286 (24.5)	18,275 (20.6)	18,275 (20.6)	10,551 (22.5)	39,464 (26.1)	20,747 (20.7)

**Quartile 2**	104,029 (24.9)	86,028 (25.0)	9,239 (24.0)	8,344 (24.1)	103,165 (25.0)	103,163 (25.0)	21,943 (24.8)	21,942 (24.8)	12,472 (26.6)	41,281 (27.3)	24,899 (24.8)
**Quartile 3**	105,757 (25.4)	87,511 (25.4)	10,714 (27.9)	9,653 (27.9)	104,852 (25.4)	105,136 (25.4)	23,642 (26.7)	23,641 (26.7)	12,932 (27.6)	37,694 (24.9)	26,837 (26.7)
**Quartile 4**	104,070 (25.0)	86,349 (25.1)	11,368 (29.6)	10,180 (29.4)	103,329 (25.0)	103,627 (25.1)	24,699 (27.9)	24,699 (27.9)	10,978 (23.4)	32,698 (21.6)	27,904 (27.8)
**Regular exercise, yes (N, %)**	227,192 (54.5)	189,477 (55.1)	21,882 (57.0)	19,676 (56.9)	27,437 (6.6)	27,221 (6.6)	4,049 (4.6)	4,049 (4.6)	3,225 (6.9)	9,868 (6.5)	4,662 (4.6)
**Diagnosis of CVD at baseline, yes (N, %)**	27,716 (6.6)	22,700 (6.6)	1,370 (3.6)	1,269 (3.7)	27,437 (6.6)	27,221 (6.6)	4,049 (4.6)	4,049 (4.6)	3,225 (6.9)	9,868 (6.5)	4,662 (4.6)
**Diagnosis of cancer at baseline, yes (N, %)**	35,760 (8.6)	28,955 (8.4)	2,432 (6.3)	2,252 (6.5)	35,483 (8.6)	35,446 (8.6)	7,087 (8.0)	7,087 (8.0)	4,262 (9.1)	14,012 (9.3)	8,170 (8.1)
**24-hour noise, mean (SD), dB**	56.04 (4.28)	56.04 (4.28)	56.02 (4.23)	56.02 (4.23)	56.04 (4.28)	56.04 (4.28)	55.98 (4.24)	55.98 (4.24)	55.95 (4.19)	55.97 (4.20)	55.98 (4.23)
**Night-time noise, mean (SD), dB**	46.58 (4.28)	46.58 (4.28)	46.56 (4.23)	46.56 (4.23)	46.58 (4.28)	46.58 (4.28)	46.52 (4.24)	46.52 (4.24)	46.48 (4.19)	46.50 (4.20)	46.52 (4.23)
**16-hour noise, mean (SD), dB**	54.46 (4.28)	54.46 (4.28)	54.44 (4.23)	54.44 (4.23)	54.46 (4.28)	54.46 (4.28)	54.40 (4.24)	54.40 (4.24)	54.37 (4.19)	54.39 (4.20)	54.40 (4.23)
**Evening noise, mean (SD), dB**	51.66 (4.28)	51.65 (4.28)	51.63 (4.23)	51.64 (4.23)	51.66 (4.28)	51.65 (4.28)	51.60 (4.24)	51.60 (4.24)	51.56 (4.19)	51.58 (4.20)	51.60 (4.23)
**Day-time noise, mean (SD), dB)**	55.40 (4.28)	55.40 (4.28)	55.38 (4.23)	55.38 (4.23)	55.40 (4.28)	55.39 (4.28)	55.34 (4.24)	55.34 (4.24)	55.30 (4.19)	55.32 (4.20)	55.34 (4.23)
**Average PM_{10}, mean (SD), micro-g/m^3**	19.30 (1.95)	19.29 (1.95)	18.87 (1.88)	18.89 (1.88)	19.30 (1.96)	19.29 (1.95)	19.29 (2.03)	19.29 (2.03)	18.82 (1.94)	19.50 (2.05)	19.30 (2.03)
**$PM_{2.5-10}$; 2010, mean (SD), micro-g/m^3**	6.42 (0.89)	6.42 (0.89)	6.36 (0.86)	6.37 (0.87)	6.42 (0.89)	6.42 (0.89)	6.40 (0.88)	6.40 (0.88)	6.41 (0.91)	6.43 (0.85)	6.41 (0.88)

**$PM_{2.5}$; 2010, mean (SD), micro-g/m^3**	9.98 (1.05)	9.98 (1.05)	9.93 (1.05)	9.93 (1.05)	9.98 (1.05)	9.98 (1.05)	9.90 (1.04)	9.90 (1.04)	9.81 (0.97)	9.88 (0.91)	9.91 (1.04)
**Average nitrogen oxides, mean (SD), micro-g/m^3**	43.96 (15.57)	43.87 (15.55)	42.80 (14.74)	42.81 (14.81)	43.90 (15.58)	43.90 (15.53)	42.91 (15.57)	42.91 (15.57)	40.71 (14.08)	43.61 (14.47)	42.99 (15.54)
**Average nitrogen dioxide, mean (SD), micro-g/m^3**	29.41 (9.28)	29.34 (9.26)	28.37 (8.78)	28.44 (8.81)	29.35 (9.28)	29.37 (9.26)	29.17 (9.80)	29.17 (9.80)	25.87 (8.05)	30.51 (9.30)	29.21 (9.76)
**Green space percentage, buffer1,000m, mean (SD), %**	45.21 (21.58)	45.36 (21.63)	47.59 (21.87)	47.44 (21.88)	45.32 (21.62)	45.28 (21.59)	45.58 (22.45)	45.58 (22.45)	51.19 (23.09)	42.49 (21.53)	45.51 (22.39)
**Blue space percentage, buffer1,000m mean (SD), %**	1.26 (2.46)	1.26 (2.48)	1.29 (2.53)	1.29 (2.54)	1.26 (2.47)	1.26 (2.47)	1.30 (2.55)	1.30 (2.55)	1.20 (2.28)	1.27 (2.71)	1.30 (2.53)
**Green space percentage, buffer300m, mean (SD), %**	35.42 (23.21)	35.54 (23.26)	37.63 (23.94)	37.51 (23.94)	35.51 (23.25)	35.47 (23.23)	35.88 (24.18)	35.88 (24.19)	40.66 (25.79)	33.11 (23.08)	35.80 (24.11)
**Blue space percentage, buffer300m, mean (SD), %**	0.89 (2.92)	0.89 (2.93)	0.94 (3.18)	0.94 (3.13)	0.89 (2.93)	0.89 (2.93)	0.95 (3.13)	0.95 (3.13)	0.85 (2.68)	0.87 (3.03)	0.94 (3.09)

Note: IDPs, imaging-derived phenotypes. BMI, body mass index. SD, standard deviation. Data are presented as means ± standard deviations

Supplementary Table 2. The associations of multiplexed environmental factors with multidimensional aging metrics using linear regression models.

Aging metrics		PM_{10}	$PM_{2.5}$	$PM_{2.5-10}$	NO_x	NO_2	Nighttime noise	24h averaged noise	Blue space	Green space
**Frailty Phenotype Score**	**Coefficient**	0.015	0.023	0.001	0.017	0.011	-0.004	-0.004	-0.001	-0.025
	**SEM**	0.002	0.002	0.001	0.001	0.002	0.001	0.001	0.001	0.002
	***P* value**	1.06×10^{-19}	3.07×10^{-43}	4.34×10^{-1}	3.69×10^{-30}	2.42×10^{-11}	6.69×10^{-5}	6.72×10^{-5}	4.70×10^{-2}	1.93×10^{-33}
	***FDR P* value**	2.16×10^{-18}	2.92×10^{-41}	5.24×10^{-1}	1.50×10^{-28}	4.06×10^{-10}	4.35×10^{-4}	4.35×10^{-4}	9.50×10^{-2}	9.17×10^{-32}
**PhenoAge**	**Coefficient**	0.032	-0.018	0.020	-0.028	-0.037	-0.019	-0.019	-0.001	<0.001
	**SEM**	0.012	0.012	0.008	0.010	0.012	0.007	0.007	0.004	0.015
	***P* value**	6.30×10^{-3}	1.34×10^{-1}	1.29×10^{-2}	7.35×10^{-3}	2.22×10^{-3}	9.04×10^{-3}	9.04×10^{-3}	7.52×10^{-1}	9.93×10^{-1}
	***FDR P* value**	1.89×10^{-2}	2.09×10^{-1}	3.37×10^{-2}	2.16×10^{-2}	8.65×10^{-3}	2.50×10^{-2}	2.50×10^{-2}	8.15×10^{-1}	9.93×10^{-1}
**Brain Age**	**Coefficient**	0.065	0.096	0.020	0.076	0.068	0.020	0.020	-0.005	-0.032
	**SEM**	0.017	0.017	0.012	0.015	0.017	0.011	0.011	0.005	0.020
	***P* value**	1.22×10^{-4}	1.30×10^{-8}	9.30×10^{-2}	6.98×10^{-7}	9.56×10^{-5}	5.34×10^{-2}	5.34×10^{-2}	3.29×10^{-1}	1.17×10^{-1}
	***FDR P* value**	7.09×10^{-4}	1.43×10^{-7}	1.56×10^{-1}	6.86×10^{-6}	5.80×10^{-4}	1.04×10^{-1}	1.04×10^{-1}	4.28×10^{-1}	1.88×10^{-1}
**Gray Matter Volume**	**Coefficient**	-0.034	-0.022	-0.007	-0.020	-0.031	-0.006	-0.006	-0.002	0.024
	**SEM**	0.005	0.005	0.004	0.005	0.005	0.003	0.003	0.002	0.006
	***P* value**	2.63×10^{-11}	1.78×10^{-5}	3.65×10^{-2}	1.15×10^{-5}	2.09×10^{-9}	4.63×10^{-2}	4.61×10^{-2}	3.03×10^{-1}	7.38×10^{-5}
	***FDR P* value**	4.16×10^{-10}	1.34×10^{-4}	8.19×10^{-2}	9.36×10^{-5}	2.71×10^{-8}	9.43×10^{-2}	9.43×10^{-2}	4.03×10^{-1}	4.67×10^{-4}
**White Matter Volume**	**Coefficient**	-0.033	-0.017	0.001	-0.018	-0.028	-0.008	-0.008	-0.002	0.023
	**SEM**	0.007	0.006	0.005	0.006	0.007	0.004	0.004	0.002	0.008
	***P* value**	2.96×10^{-7}	8.40×10^{-3}	8.31×10^{-1}	2.65×10^{-3}	2.05×10^{-5}	4.32×10^{-2}	4.29×10^{-2}	3.08×10^{-1}	2.55×10^{-3}
	***FDR P* value**	3.01×10^{-6}	2.39×10^{-2}	8.77×10^{-1}	9.94×10^{-3}	1.46×10^{-4}	9.12×10^{-2}	9.12×10^{-2}	4.04×10^{-1}	9.69×10^{-3}
**Brain Volume**	**Coefficient**	-0.041	-0.024	-0.004	-0.023	-0.037	-0.009	-0.009	-0.002	0.029

**SEM**	0.006	0.006	0.004	0.005	0.006	0.003	0.003	0.002	0.007
***P* value**	2.84×10^{-13}	1.96×10^{-5}	2.68×10^{-1}	4.95×10^{-6}	2.34×10^{-10}	1.24×10^{-2}	1.23×10^{-2}	2.02×10^{-1}	1.55×10^{-5}
***FDR P* value**	5.06×10^{-12}	1.43×10^{-4}	3.65×10^{-1}	4.41×10^{-5}	3.18×10^{-9}	3.28×10^{-2}	3.28×10^{-2}	2.89×10^{-1}	1.19×10^{-4}

Note: FDR, false discovery rate. SEM, standard error of mean; NO_x, nitrogen oxides; NO_2, nitrogen dioxide; $PM_{2.5}$, particulate matter with aerodynamic diameter ≤2.5µm; $PM_{2.5-10}$, particulate matter with aerodynamic diameter between 2.5µm and 10µm; PM_{10}, particulate matter with aerodynamic diameter ≤10µm. All environmental factors were computed as the interquartile range (IQR). All models were adjusted for age, sex, ethnicity, nSES, smoking status, BMI (category variable), alcohol intake frequency, regular exercise, healthy diet, history of CVD, and cancer at baseline. The volumes were normalized by the Z scores method. Two-sided P value of <0.05 was considered as statistically significant. Benjamini–Hochberg procedure was used to control the family-wise error rate in the main analyses (n = 285).

Supplementary Table 3. Contributions of multiplexed environmental factors to multidimensional aging metrics while adjusting for iSES.

Factors		Frailty Phenotype Score	PhenoAge	Brain Age	Brain Volume	White Matter Volume	Gray Matter Volume
**Joint effects**	**Coefficient**	0.098	0.043	0.072	-2170.500	-821.320	-1311.620
	**SEM**	0.013	0.002	0.019	525.100	344.120	306.150
	***P* value**	<0.001	<0.001	<0.001	<0.001	0.017	<0.001
**Proportion of Individual Contribution**	**PM_{10}**	0.146	0.677	0.570	0.319	0.143	0.482
	**$PM_{2.5-10}$**	0.022	0.075	0.011	0.004	0.003	0.012
	**$PM_{2.5}$**	0.010	0.001	0.264	0.028	0.088	0.009
	**NO_2**	0.508	0.108	0.003	<0.001	<0.001	0.001
	**NO_x**	0.192	<0.001	0.075	0.034	0.026	0.057
	**Blue space**	0.019	0.002	0.026	0.063	0.121	0.038
	**Green space**	0.101	0.133	0.002	0.394	0.490	0.217
	**Nighttime noise**	0.001	0.001	0.027	0.127	0.106	0.125
	**24h averaged noise**	<0.001	0.003	0.022	0.031	0.022	0.058

Note: SEM, standard error of mean; WQS, weighted quantile sum; NO_x, nitrogen oxides; NO_2, nitrogen dioxide; $PM_{2.5}$, particulate matter with aerodynamic diameter ≤2.5μm; $PM_{2.5-10}$, particulate matter with aerodynamic diameter between 2.5μm and 10μm; PM_{10}, particulate matter with aerodynamic diameter ≤10μm. All models were adjusted for age, sex, ethnicity, iSES, smoking status, BMI (category variable), alcohol intake frequency, regular exercise, healthy diet, history of CVD, and cancer at baseline. Two-sided P value of <0.05 was considered as statistically significant.

Supplementary Table 4. Contributions of multiplexed environmental factors to volumes of regional GM volumes and subcortical areas while adjusting for iSES.

IDPs		PM_{10}	$PM_{2.5}$	$PM_{2.5\text{-}10}$	NO_x	NO_2	Nighttime noise	24h averaged noise	Blue space	Green space
**Superior frontal gyri**	**Coefficient**	-0.014	-0.009	-0.007	-0.008	-0.002	-0.006	-0.006	-0.002	-0.001
	**SEM**	0.006	0.006	0.005	0.005	0.006	0.004	0.004	0.002	0.008
	***P* value**	0.030	0.167	0.124	0.154	0.702	0.120	0.120	0.348	0.894
	***FDR P* value**	0.076	0.267	0.211	0.249	0.776	0.210	0.210	0.459	0.913
**Inferior frontal gyri**	**Coefficient**	-0.017	-0.007	-0.002	-0.011	-0.009	-0.009	-0.009	-0.005	0.006
	**SEM**	0.006	0.006	0.005	0.005	0.006	0.004	0.004	0.002	0.007
	***P* value**	0.005	0.215	0.584	0.047	0.131	0.017	0.017	0.020	0.410
	***FDR P* value**	0.022	0.316	0.684	0.110	0.218	0.055	0.055	0.059	0.526
**Middle frontal gyri**	**Coefficient**	-0.006	-0.009	-0.002	-0.007	-0.003	-0.005	-0.005	-0.001	0.008
	**SEM**	0.006	0.006	0.005	0.006	0.006	0.004	0.004	0.002	0.008
	***P* value**	0.310	0.142	0.608	0.177	0.614	0.248	0.248	0.795	0.286
	***FDR P* value**	0.419	0.233	0.702	0.274	0.704	0.358	0.358	0.834	0.394
**Precuneus**	**Coefficient**	-0.032	-0.026	-0.011	-0.024	-0.023	-0.004	-0.004	-0.001	0.023
	**SEM**	0.006	0.006	0.005	0.005	0.006	0.004	0.004	0.002	0.007
	***P* value**	<0.001	<0.001	0.015	<0.001	<0.001	0.278	0.278	0.606	0.002
	***FDR P* value**	<0.001	<0.001	0.050	<0.001	0.001	0.385	0.385	0.702	0.007
**Supplementary motor cortex**	**Coefficient**	-0.016	-0.008	-0.008	-0.008	-0.004	-0.008	-0.008	-0.004	0.006
	**SEM**	0.006	0.006	0.005	0.006	0.006	0.004	0.004	0.002	0.008
	***P* value**	0.016	0.210	0.102	0.168	0.501	0.068	0.069	0.079	0.464
	***FDR P* value**	0.052	0.311	0.187	0.267	0.606	0.148	0.148	0.162	0.573
**Precentral gyrus**	**Coefficient**	-0.023	-0.028	-0.003	-0.020	-0.014	-0.006	-0.006	-0.001	0.011
	**SEM**	0.006	0.006	0.004	0.005	0.006	0.004	0.004	0.002	0.007

	***P* value**	<0.001	<0.001	0.542	<0.001	0.016	0.100	0.100	0.616	0.114
	***FDR P* value**	0.001	<0.001	0.645	<0.001	0.052	0.187	0.187	0.704	0.204
**Postcentral gyrus**	**Coefficient**	-0.011	-0.014	-0.009	-0.007	<0.001	0.003	0.003	-0.001	0.004
	**SEM**	0.006	0.006	0.004	0.005	0.006	0.004	0.004	0.002	0.007
	***P* value**	0.045	0.013	0.027	0.144	0.994	0.430	0.431	0.720	0.557
	***FDR P* value**	0.108	0.046	0.074	0.234	0.994	0.547	0.547	0.783	0.657
**Superior Parietal lobe**	**Coefficient**	-0.020	-0.014	-0.002	-0.016	-0.015	-0.005	-0.005	0.001	0.012
	**SEM**	0.006	0.006	0.005	0.005	0.006	0.004	0.004	0.002	0.008
	***P* value**	0.001	0.022	0.658	0.003	0.012	0.201	0.201	0.521	0.121
	***FDR P* value**	0.006	0.062	0.745	0.013	0.044	0.302	0.302	0.627	0.210
**Parahippocampal gyrus**	**Coefficient**	-0.015	-0.002	-0.007	-0.002	-0.005	-0.010	-0.010	-0.001	-0.004
	**SEM**	0.006	0.006	0.005	0.005	0.006	0.004	0.004	0.002	0.007
	***P* value**	0.019	0.710	0.142	0.697	0.382	0.014	0.014	0.722	0.554
	***FDR P* value**	0.059	0.781	0.233	0.775	0.495	0.046	0.046	0.783	0.656
**Middle temporal gyrus**	**Coefficient**	-0.014	-0.006	-0.004	-0.010	-0.007	-0.009	-0.009	-0.002	0.013
	**SEM**	0.006	0.006	0.005	0.005	0.006	0.004	0.004	0.002	0.007
	***P* value**	0.021	0.291	0.325	0.071	0.267	0.029	0.029	0.240	0.087
	***FDR P* value**	0.059	0.398	0.437	0.149	0.377	0.074	0.074	0.351	0.174
**Inferior temporal gyrus**	**Coefficient**	-0.014	-0.012	-0.002	-0.015	-0.010	-0.011	-0.011	<0.001	0.003
	**SEM**	0.006	0.006	0.005	0.006	0.006	0.004	0.004	0.002	0.008
	***P* value**	0.034	0.067	0.698	0.006	0.101	0.010	0.010	0.830	0.691
	***FDR P* value**	0.085	0.147	0.775	0.023	0.187	0.037	0.037	0.868	0.774
**Hippocampus**	**Coefficient**	-0.020	<0.001	-0.003	-0.003	-0.012	-0.007	-0.007	-0.006	0.002
	**SEM**	0.006	0.006	0.004	0.005	0.006	0.004	0.004	0.002	0.007
	***P* value**	0.001	0.949	0.453	0.526	0.043	0.094	0.093	0.002	0.770
	***FDR P* value**	0.004	0.957	0.566	0.629	0.106	0.182	0.182	0.011	0.816

**Putamen**	**Coefficient**	0.024	0.009	0.006	0.010	0.013	-0.001	-0.001	0.001	-0.006
	**SEM**	0.007	0.006	0.005	0.006	0.006	0.004	0.004	0.002	0.008
	***P* value**	<0.001	0.169	0.253	0.070	0.049	0.877	0.878	0.775	0.464
	***FDR P* value**	0.001	0.267	0.361	0.148	0.114	0.901	0.901	0.818	0.573
**Thalamus**	**Coefficient**	-0.011	0.011	-0.002	0.001	-0.007	-0.010	-0.010	-0.005	0.008
	**SEM**	0.006	0.006	0.005	0.006	0.006	0.004	0.004	0.002	0.008
	***P* value**	0.093	0.082	0.638	0.848	0.271	0.020	0.020	0.035	0.294
	***FDR P* value**	0.182	0.167	0.726	0.881	0.380	0.059	0.059	0.088	0.400
**Caudate**	**Coefficient**	0.015	0.017	0.006	0.019	0.009	0.004	0.004	<0.001	<0.001
	**SEM**	0.006	0.006	0.005	0.006	0.006	0.004	0.004	0.002	0.008
	***P* value**	0.018	0.006	0.172	0.001	0.173	0.349	0.350	0.850	0.965
	***FDR P* value**	0.058	0.022	0.270	0.003	0.271	0.459	0.459	0.881	0.970
**Amygdala**	**Coefficient**	-0.011	-0.010	-0.008	-0.009	-0.012	-0.008	-0.008	-0.002	0.011
	**SEM**	0.006	0.006	0.004	0.005	0.006	0.004	0.004	0.002	0.007
	***P* value**	0.064	0.084	0.076	0.075	0.028	0.024	0.024	0.392	0.125
	***FDR P* value**	0.143	0.168	0.157	0.155	0.074	0.066	0.066	0.506	0.211

Note: FDR, false discovery rate. NO_x, nitrogen oxides; NO_2, nitrogen dioxide; $PM_{2.5}$, particulate matter with aerodynamic diameter ≤2.5μm; $PM_{2.5-10}$, particulate matter with aerodynamic diameter between 2.5μm and 10 μm; PM_{10}, particulate matter with aerodynamic diameter ≤10μm. All environmental factors were computed as the interquartile range (IQR). All models were adjusted for age, sex, ethnicity, iSES, smoking status, BMI (category variable), alcohol intake frequency, regular exercise, healthy diet, history of CVD, and cancer at baseline. The volumes were normalized by the Z scores method. Two-sided P value of <0.05 was considered as statistically significant. Benjamini–Hochberg procedure was used to control the family-wise error rate in the analyses with adjustment of iSES (n = 231).

Supplementary Table 5. Multiplexed environmental factors' individual effect on cognitive performances using linear regression models while adjusting for iSES.

Cognitive Performance		PM_{10}	$PM_{2.5}$	$PM_{2.5\text{-}10}$	NO_x	NO_2	Nighttime noise	24h averaged noise	Blue space	Green space
**Pair matching task**	**Coefficient**	0.012	0.015	0.005	0.011	0.011	0.002	0.002	<0.001	-0.006
	**SEM**	0.002	0.002	0.001	0.002	0.002	0.001	0.001	0.001	0.002
	***P* value**	<0.001	<0.001	0.001	<0.001	<0.001	0.109	0.108	0.761	0.020
	***FDR P* value**	<0.001	<0.001	0.003	<0.001	<0.001	0.196	0.196	0.816	0.059
**Reaction time task**	**Coefficient**	0.034	0.025	0.008	0.023	0.039	0.001	0.001	0.001	-0.043
	**SEM**	0.002	0.002	0.001	0.002	0.002	0.001	0.001	0.001	0.002
	***P* value**	<0.001	<0.001	<0.001	<0.001	<0.001	0.444	0.443	0.354	<0.001
	***FDR P* value**	<0.001	<0.001	<0.001	<0.001	<0.001	0.558	0.558	0.462	<0.001
**Fluid intelligence test**	**Coefficient**	-0.017	-0.008	-0.016	-0.009	-0.006	0.004	0.004	0.001	-0.010
	**SEM**	0.003	0.004	0.002	0.003	0.003	0.002	0.002	0.001	0.004
	***P* value**	<0.001	0.028	<0.001	0.001	0.046	0.052	0.052	0.201	0.006
	***FDR P* value**	<0.001	0.074	<0.001	0.005	0.110	0.119	0.119	0.302	0.023
**Digit span task**	**Coefficient**	-0.010	-0.009	-0.005	-0.007	-0.007	0.006	0.006	0.001	0.003
	**SEM**	0.006	0.006	0.004	0.005	0.006	0.004	0.004	0.002	0.006
	***P* value**	0.069	0.122	0.205	0.185	0.256	0.101	0.101	0.769	0.596
	***FDR P* value**	0.148	0.210	0.305	0.285	0.363	0.187	0.187	0.816	0.695
**Trail marking A**	**Coefficient**	-0.014	0.014	0.001	0.014	-0.001	0.007	0.007	-0.002	-0.001
	**SEM**	0.004	0.004	0.003	0.003	0.004	0.003	0.003	0.001	0.005
	***P* value**	<0.001	<0.001	0.717	<0.001	0.764	0.004	0.004	0.119	0.902
	***FDR P* value**	0.001	0.003	0.783	<0.001	0.816	0.017	0.017	0.210	0.914
**Trail marking B**	**Coefficient**	0.007	0.024	0.006	0.021	0.014	0.007	0.007	-0.002	-0.010
	**SEM**	0.004	0.004	0.003	0.003	0.003	0.002	0.002	0.001	0.005

	***P* value**	0.061	<0.001	0.044	<0.001	<0.001	0.003	0.003	0.134	0.023
	***FDR P* value**	0.136	<0.001	0.108	<0.001	<0.001	0.014	0.014	0.223	0.064
**Symbol digit substitution task**	**Coefficient**	-0.010	-0.027	-0.006	-0.023	-0.016	-0.009	-0.009	0.002	0.014
	**SEM**	0.003	0.003	0.003	0.003	0.003	0.002	0.002	0.001	0.004
	***P* value**	0.002	<0.001	0.012	<0.001	<0.001	<0.001	<0.001	0.101	0.001
	***FDR P* value**	0.008	<0.001	0.044	<0.001	<0.001	<0.001	<0.001	0.187	0.003

Note: FDR, false discovery rate. NO_x, nitrogen oxides; NO_2, nitrogen dioxide; $PM_{2.5}$, particulate matter with aerodynamic diameter ≤2.5μm; $PM_{2.5-10}$, particulate matter with aerodynamic diameter between 2.5μm and 10μm; PM_{10}, particulate matter with aerodynamic diameter ≤10μm. All environmental factors were computed as the interquartile range (IQR). All models were adjusted for age, sex, ethnicity, iSES, smoking status, BMI (category variable), alcohol intake frequency, regular exercise, healthy diet, history of CVD, and cancer at baseline. The scores were first normalized, if not normally distributed, and then converted to Z scores. Two-sided P value of <0.05 was considered as statistically significant. Benjamini–Hochberg procedure was used to control the family-wise error rate in the analyses with adjustment of iSES (n = 231).

Supplementary Table 6. Associations of subpopulations with multidimensional aging metrics using linear regression models while adjusting for iSES.

		Frailty Phenotype Score	PhenoAge	Brain Age	Gray Matter Volume	White Matter Volume	Brain Volume
**Blue space Subpopulation**	**Coefficient**	0.049	0.093	0.013	-0.010	-0.044	-0.031
	**SEM**	0.010	0.073	0.101	0.030	0.039	0.033
	***P* value**	<0.001	0.198	0.899	0.740	0.253	0.347
	***FDR P* value**	<0.001	0.302	0.914	0.799	0.361	0.459
**Rural-urban fringe Subpopulation**	**Coefficient**	0.050	0.105	0.023	-0.004	-0.008	-0.007
	**SEM**	0.003	0.025	0.033	0.010	0.013	0.011
	***P* value**	<0.001	<0.001	0.475	0.680	0.498	0.493
	***FDR P* value**	<0.001	<0.001	0.584	0.766	0.605	0.602
**Noise Subpopulation**	**Coefficient**	0.053	0.080	0.088	-0.059	-0.048	-0.065
	**SEM**	0.006	0.041	0.057	0.017	0.022	0.019
	***P* value**	<0.001	0.052	0.123	0.001	0.028	<0.001
	***FDR P* value**	<0.001	0.119	0.210	0.003	0.074	0.003
**Air pollution Subpopulation**	**Coefficient**	0.092	0.174	0.124	-0.055	-0.051	-0.065
	**SEM**	0.004	0.028	0.039	0.012	0.015	0.013
	***P* value**	<0.001	<0.001	0.002	<0.001	0.001	<0.001
	***FDR P* value**	<0.001	<0.001	0.007	<0.001	0.004	<0.001

Note: FDR, false discovery rate. SEM, standard error of mean. All models were adjusted for age, sex, ethnicity, iSES, smoking status, BMI (category variable), alcohol intake frequency, regular exercise, healthy diet, history of CVD, and cancer at baseline. The volumes were normalized by the Z scores method. Two-sided P value of <0.05 was considered as statistically significant. Benjamini–Hochberg procedure was used to control the family-wise error rate in the analyses with adjustment of iSES (n = 231). We set the green space subpopulation as reference.

Supplementary Table 7. Contributions of environmental factors to multidimensional aging metrics in subpopulations that have lived at current address for no less than five years.

Factors		Frailty Phenotype Score	PhenoAge	Brain Age	Brain Volume	White Matter Volume	Gray Matter Volume
**Joint effects**	**Coefficient**	0.025	0.039	0.099	-2519.620	-1395.393	-1259.940
	**SEM**	0.002	0.016	0.023	561.080	409.602	333.380
	***P* value**	<0.001	0.019	<0.001	<0.001	0.001	<0.001
**Proportion of Individual Contribution**	**PM_{10}**	0.227	0.576	0.407	0.325	0.295	0.221
	**$PM_{2.5-10}$**	0.013	0.076	0.091	0.063	0.023	0.174
	**$PM_{2.5}$**	0.126	<0.001	0.310	0.008	0.007	0.028
	**NO_2**	0.147	0.043	0.029	0.003	0.002	0.011
	**NO_x**	0.353	<0.001	0.015	0.127	0.171	0.079
	**Blue space**	0.125	0.116	<0.001	0.034	0.104	0.014
	**Green space**	0.008	0.069	<0.001	0.366	0.210	0.448
	**Nighttime noise**	<0.001	0.095	0.144	0.020	0.035	0.009
	**24h averaged noise**	<0.001	0.025	0.004	0.055	0.154	0.015

Note: SEM, standard error of mean; WQS, weighted quantile sum; NO_x, nitrogen oxides; NO_2, nitrogen dioxide; $PM_{2.5}$, particulate matter with aerodynamic diameter ≤2.5μm; $PM_{2.5-10}$, particulate matter with aerodynamic diameter between 2.5μm and 10 μm; PM_{10}, particulate matter with aerodynamic diameter ≤10μm. All models were adjusted for age, sex, ethnicity, nSES, smoking status, BMI (category variable), alcohol intake frequency, regular exercise, healthy diet, history of CVD, and cancer at baseline. Two-sided P value of <0.05 was considered as statistically significant.

Supplementary Table 8. Contributions of multiplexed environmental factors to multidimensional aging metrics in subpopulations that have lived at current address for no less than five years.

		Frailty Phenotype Score	PhenoAge	Brain Age	Gray Matter Volume	White Matter Volume	Brain Volume
**Blue space Subpopulation**	**Coefficient**	0.022	0.033	-0.203	-0.006	-0.027	-0.019
	**SEM**	0.011	0.075	0.109	0.032	0.041	0.036
	***P* value**	0.035	0.663	0.062	0.858	0.515	0.596
	***FDR P* value**	0.127	0.814	0.191	0.932	0.708	0.753
**Rural-urban fringe Subpopulation**	**Coefficient**	0.042	0.092	0.034	-0.007	-0.005	-0.007
	**SEM**	0.004	0.025	0.034	0.010	0.013	0.011
	***P* value**	<0.001	<0.001	0.316	0.519	0.674	0.510
	***FDR P* value**	<0.001	0.002	0.529	0.708	0.814	0.706
**Noise Subpopulation**	**Coefficient**	0.027	-0.033	0.111	-0.055	-0.037	-0.057
	**SEM**	0.006	0.042	0.060	0.018	0.023	0.020
	***P* value**	<0.001	0.440	0.063	0.002	0.101	0.004
	***FDR P* value**	<0.001	0.670	0.191	0.012	0.258	0.019
**Air pollution Subpopulation**	**Coefficient**	0.043	0.024	0.138	-0.048	-0.036	-0.052
	**SEM**	0.004	0.030	0.043	0.013	0.017	0.014
	***P* value**	<0.001	0.431	0.001	<0.001	0.031	<0.001
	***FDR P* value**	<0.001	0.661	0.009	0.002	0.114	0.002

Note: FDR, false discovery rate. SEM, standard error of mean. All models were adjusted for age, sex, ethnicity, nSES, BMI (category variable), smoking status regular exercise, healthy diet, history of CVD, and cancer at baseline. The volumes were normalized by the Z scores method. Two-sided P value of <0.05 was considered as statistically significant. Benjamini–Hochberg procedure was used to control the family-wise error rate in the subgroup analyses (n = 288). We set the green space subpopulation as reference.

Supplementary Table 9. Subgroup analyses of contributions of environmental factors to multidimensional aging metrics stratified by sex.

Sex	Factors		Frailty Phenotype Score	PhenoAge	Brain Age	Brain Volume	White Matter Volume	Gray Matter Volume
Male	Joint effects	Coefficient	0.028	0.048	0.076	-2152.940	-1046.850	-1039.600
		SEM	0.003	0.022	0.020	541.610	357.000	323.760
		P value	<0.001	0.026	<0.001	<0.001	0.003	0.001
	Proportion of Individual Contribution	PM_{10}	0.054	0.455	0.253	0.527	0.545	0.253
		$PM_{2.5\text{-}10}$	0.028	0.244	0.122	0.005	0.001	0.122
		$PM_{2.5}$	0.075	0.169	0.050	0.009	0.003	0.050
		NO_2	0.240	0.053	0.165	0.024	0.004	0.165
		NO_x	0.492	0.005	0.125	0.025	0.006	0.125
		Blue space	0.061	0.055	0.007	0.005	0.010	0.007
		Green space	0.026	0.003	0.129	0.207	0.221	0.129
		Nighttime noise	0.007	0.012	0.110	0.136	0.107	0.110
		24h averaged noise	0.017	0.004	0.110	0.136	0.107	0.110
Female	Joint effects	Coefficient	0.018	0.036	0.072	-1207.960	-450.780	-897.200
		SEM	0.003	0.020	0.029	725.360	521.330	406.320
		P value	<0.001	0.073	0.013	0.096	0.387	0.027
	Proportion of Individual Contribution	PM_{10}	0.072	0.317	0.535	0.474	0.257	0.535
		$PM_{2.5\text{-}10}$	0.022	0.455	0.043	0.045	0.073	0.043
		$PM_{2.5}$	0.551	<0.001	0.037	0.001	<0.001	0.037
		NO_2	0.074	0.048	0.064	0.026	0.011	0.064
		NO_x	0.178	<0.001	0.097	0.048	0.028	0.097
		Blue space	0.101	0.098	0.005	0.011	0.064	0.005
		Green space	0.001	0.017	0.069	0.128	0.197	0.069

		**Nighttime noise**	<0.001	0.049	0.128	0.218	0.259	0.128
		**24h averaged noise**	<0.001	0.016	0.128	0.218	0.259	0.128

Note: SEM, standard error of mean; WQS, weighted quantile sum; NO_x, nitrogen oxides; NO_2, nitrogen dioxide; $PM_{2.5}$, particulate matter with aerodynamic diameter ≤2.5μm; $PM_{2.5-10}$, particulate matter with aerodynamic diameter between 2.5μm and 10μm; PM_{10}, particulate matter with aerodynamic diameter ≤10μm. All models were adjusted for age, ethnicity, nSES, smoking status, BMI (category variable), alcohol intake frequency, regular exercise, healthy diet, history of CVD, and cancer at baseline. Two-sided P value of <0.05 was considered as statistically significant.

Supplementary Table 10. Subgroup analyses of contributions of environmental factors to multidimensional aging metrics stratified by age.

Age	Factors		Frailty Phenotype Score	PhenoAge	Brain Age	Brain Volume	White Matter Volume	Gray Matter Volume
< 60 years old	Joint effects	Coefficient	0.019	-0.215	0.049	-847.100	-425.200	-334.900
		SEM	0.003	0.028	0.030	821.700	468.500	515.900
		P value	<0.001	<0.001	0.103	0.303	0.364	0.516
	Proportion of Individual Contribution	PM_{10}	0.042	0.331	0.385	0.216	0.222	0.176
		$PM_{2.5-10}$	0.191	0.383	0.073	0.173	0.151	0.187
		$PM_{2.5}$	0.078	0.002	0.073	0.120	0.143	0.083
		NO_2	0.032	0.049	0.093	<0.001	0.003	<0.001
		NO_x	0.539	<0.001	0.097	0.074	0.102	0.056
		Blue space	0.106	0.085	0.005	0.152	0.075	0.266
		Green space	0.012	0.148	0.002	0.173	0.210	0.130
		Nighttime noise	<0.001	<0.001	0.040	0.042	0.059	0.028
		24h averaged noise	<0.001	0.002	0.264	0.048	0.034	0.073
≥ 60 years old	Joint effects	Coefficient	0.024	0.025	0.033	-1087.400	-286.370	-890.800
		SEM	0.003	0.027	0.020	895.900	543.690	654.900
		P value	<0.001	0.351	0.108	0.225	0.598	0.174
	Proportion of Individual Contribution	PM_{10}	0.013	0.506	0.692	0.427	0.496	0.175
		$PM_{2.5-10}$	0.053	0.046	0.003	0.007	0.131	<0.001
		$PM_{2.5}$	0.385	0.001	0.083	<0.001	0.001	0.004
		NO_2	0.030	0.076	0.068	0.036	0.013	0.130
		NO_x	0.451	<0.001	0.001	0.025	0.023	0.047
		Blue space	0.064	0.362	0.055	0.026	0.015	0.079

		**Green space**	0.003	0.004	0.037	0.323	0.223	0.350
		**Nighttime noise**	0.001	0.004	0.041	0.136	0.077	0.171
		**24h averaged noise**	<0.001	0.003	0.021	0.020	0.020	0.044

Note: SEM, standard error of mean; WQS, weighted quantile sum; NO_x, nitrogen oxides; NO_2, nitrogen dioxide; $PM_{2.5}$, particulate matter with aerodynamic diameter ≤2.5μm; $PM_{2.5-10}$, particulate matter with aerodynamic diameter between 2.5μm and 10μm; PM_{10}, particulate matter with aerodynamic diameter ≤10μm. All models were adjusted for sex, ethnicity, nSES, smoking status, BMI (category variable), alcohol intake frequency, regular exercise, healthy diet, history of CVD, and cancer at baseline. Two-sided P value of <0.05 was considered as statistically significant.

Supplementary Table 11. Subgroup analyses of contributions of environmental factors to multidimensional aging metrics stratified by smoking status.

Smoking status	Factors		Frailty Phenotype Score	PhenoAge	Brain Age	Brain Volume	White Matter Volume	Gray Matter Volume
Never smoker	Joint effects	Coefficient	0.028	0.068	0.088	-2574.100	-1588.320	-1067.880
		SEM	0.003	0.020	0.033	723.030	415.630	476.840
		P value	<0.001	0.001	0.007	<0.001	<0.001	0.025
	Proportion of Individual Contribution	PM_{10}	0.057	0.331	0.104	0.255	0.307	0.106
		$PM_{2.5-10}$	0.017	0.383	0.058	0.067	0.207	0.016
		$PM_{2.5}$	0.414	0.002	0.030	0.065	0.019	0.154
		NO_2	0.071	0.049	0.258	0.004	0.002	0.015
		NO_x	0.297	<0.001	0.103	0.194	0.226	0.093
		Blue space	0.110	0.085	0.179	0.007	0.003	0.047
		Green space	0.034	0.148	<0.001	0.180	0.076	0.309
		Nighttime noise	<0.001	<0.001	0.037	0.031	0.031	0.045
		24h averaged noise	<0.001	0.002	0.229	0.196	0.130	0.216
Ever smoker	Joint effects	Coefficient	0.017	0.017	0.066	-1207.960	-561.010	-987.000
		SEM	0.004	0.024	0.032	725.360	588.770	706.040
		P value	<0.001	0.465	0.041	0.096	0.341	0.162
	Proportion of Individual Contribution	PM_{10}	0.151	0.709	0.132	0.206	0.219	0.122
		$PM_{2.5-10}$	0.003	0.063	0.029	0.013	0.003	0.051
		$PM_{2.5}$	0.288	<0.001	0.531	0.078	0.029	0.119
		NO_2	0.030	0.017	0.158	0.009	0.019	0.006
		NO_x	0.200	<0.001	0.092	0.016	0.009	0.053
		Blue space	0.142	0.186	0.017	0.476	0.386	0.407
		Green space	0.162	0.020	0.010	0.050	0.273	0.007
		Nighttime noise	0.020	0.003	0.021	0.092	0.019	0.184

		**24h averaged noise**	0.003	0.001	0.010	0.059	0.044	0.051
**Current smoker**	**Joint effects**	**Coefficient**	0.007	-0.033	-0.031	-2624.200	-3180.360	367.500
		**SEM**	0.007	0.047	0.099	2468.200	1431.530	1561.300
		***P* value**	0.274	0.480	0.754	0.288	0.026	0.814
	**Proportion of Individual Contribution**	**PM_{10}**	0.008	<0.001	0.431	0.092	0.126	0.048
		**$PM_{2.5\text{-}10}$**	0.012	<0.001	0.002	0.071	0.145	0.029
		**$PM_{2.5}$**	0.275	0.264	0.017	0.019	0.019	0.030
		**NO_2**	0.050	<0.001	0.064	0.236	0.210	0.126
		**NO_x**	0.534	0.527	0.048	0.218	0.099	0.212
		**Blue space**	0.065	0.151	0.321	0.133	0.127	0.132
		**Green space**	0.034	0.020	0.007	0.096	0.263	0.019
		**Nighttime noise**	0.016	<0.001	0.037	0.061	0.007	0.135
		**24h averaged noise**	0.005	0.038	0.072	0.074	0.004	0.269

Note: SEM, standard error of mean; WQS, weighted quantile sum; NO_x, nitrogen oxides; NO_2, nitrogen dioxide; $PM_{2.5}$, particulate matter with aerodynamic diameter ≤2.5μm; $PM_{2.5\text{-}10}$, particulate matter with aerodynamic diameter between 2.5μm and 10μm; PM_{10}, particulate matter with aerodynamic diameter ≤10μm. All models were adjusted for age, sex, ethnicity, nSES, smoking status, BMI (category variable), alcohol intake frequency, regular exercise, healthy diet, history of CVD, and cancer at baseline. Two-sided P value of <0.05 was considered as statistically significant.

Supplementary Table 12. Subgroup analyses of contributions of environmental factors to multidimensional aging metrics stratified by alcohol intake frequency.

Alcohol intake frequency	Factors		Frailty Phenotype Score	PhenoAge	Brain Age	Brain Volume	White Matter Volume	Gray Matter Volume
Never or special occasions only	Joint effects	Coefficient	0.030	0.059	0.032	-2517.420	-1593.300	-795.470
		SEM	0.006	0.036	0.057	1720.690	1136.500	1022.600
		P value	<0.001	0.100	0.576	0.144	0.161	0.437
	Proportion of Individual Contribution	PM_{10}	0.331	0.445	0.203	0.355	0.352	0.375
		$PM_{2.5-10}$	0.046	0.447	0.071	0.072	0.012	0.037
		$PM_{2.5}$	0.017	<0.001	0.021	0.039	0.024	0.016
		NO_2	0.059	0.006	0.340	0.007	0.022	<0.001
		NO_x	0.235	<0.001	0.017	0.006	0.001	0.033
		Blue space	0.121	0.063	<0.001	0.098	0.200	0.252
		Green space	0.191	0.025	0.302	0.125	0.199	0.074
		Nighttime noise	<0.001	0.006	0.038	0.189	0.079	0.010
		24h averaged noise	<0.001	0.009	0.010	0.110	0.110	0.202
One to three times per month	Joint effects	Coefficient	0.012	0.126	-0.003	-366.710	-923.870	554.120
		SEM	0.006	0.050	0.078	1294.280	851.370	873.790
		P value	0.053	0.011	0.965	0.777	0.278	0.526
	Proportion of Individual Contribution	PM_{10}	0.472	0.493	0.039	0.001	0.001	0.001
		$PM_{2.5-10}$	0.200	0.019	0.373	0.051	0.047	0.091
		$PM_{2.5}$	0.044	0.009	0.095	0.036	0.022	0.081
		NO_2	0.011	0.009	0.038	0.003	<0.001	0.044
		NO_x	0.207	0.026	0.044	0.002	0.004	0.021

		**Blue space**	0.018	0.126	0.228	0.099	0.131	0.080
		**Green space**	0.009	<0.001	0.001	0.011	0.016	0.018
		**Nighttime noise**	0.018	0.001	0.032	0.487	0.680	0.080
		**24h averaged noise**	0.021	0.315	0.150	0.311	0.099	0.583
**One to four times per week**	**Joint effects**	**Coefficient**	0.028	0.025	0.066	-2010.940	-514.240	-1254.690
		**SEM**	0.003	0.020	0.029	679.550	458.630	432.200
		***P* value**	<0.001	0.211	0.024	0.003	0.262	0.004
	**Proportion of Individual Contribution**	**PM_{10}**	0.036	0.573	0.086	0.238	0.082	0.445
		**$PM_{2.5-10}$**	0.132	0.161	0.011	0.004	0.025	0.044
		**$PM_{2.5}$**	0.376	0.030	0.395	0.316	0.160	0.015
		**NO_2**	0.138	0.002	0.027	0.001	<0.001	0.034
		**NO_x**	0.095	0.003	0.164	0.217	0.445	0.046
		**Blue space**	0.153	0.164	0.023	0.027	0.056	0.026
		**Green space**	0.069	0.018	0.051	0.097	0.032	0.048
		**Nighttime noise**	<0.001	0.047	0.060	0.005	0.015	0.249
		**24h averaged noise**	<0.001	0.001	0.184	0.095	0.185	0.095
**Daily or almost daily**	**Joint effects**	**Coefficient**	0.025	0.074	0.006	-3505.700	-1562.260	-1261.250
		**SEM**	0.004	0.029	0.046	1135.300	864.030	585.220
		***P* value**	<0.001	0.011	0.894	0.002	0.071	0.031
	**Proportion of Individual Contribution**	**PM_{10}**	0.040	0.308	0.061	0.411	0.375	0.229
		**$PM_{2.5-10}$**	0.003	0.018	0.048	0.158	0.037	0.065
		**$PM_{2.5}$**	0.127	0.160	0.047	0.024	0.016	0.181
		**NO_2**	0.106	0.362	0.131	0.001	<0.001	0.002

		**NO_x**	0.502	0.033	0.068	0.127	0.033	0.127
		**Blue space**	0.205	0.082	0.574	0.056	0.252	0.021
		**Green space**	0.005	0.037	0.013	0.143	0.074	0.368
		**Nighttime noise**	0.007	<0.001	0.053	0.006	0.010	0.006
		**24h averaged noise**	0.004	<0.001	0.004	0.073	0.202	0.001

Note: SEM, standard error of mean; WQS, weighted quantile sum; NO_x, nitrogen oxides; NO_2, nitrogen dioxide; $PM_{2.5}$, particulate matter with aerodynamic diameter ≤2.5μm; $PM_{2.5-10}$, particulate matter with aerodynamic diameter between 2.5μm and 10 μm; PM_{10}, particulate matter with aerodynamic diameter ≤10μm. All models were adjusted for age, sex, ethnicity, nSES, smoking status, BMI (category variable), alcohol intake frequency, regular exercise, healthy diet, history of CVD, and cancer at baseline. Two-sided P value of <0.05 was considered as statistically significant.

Supplementary Table 13. Subgroup analyses of associations of subpopulations with multidimensional aging metrics using linear regression models stratified by sex.

Sex			Frailty Phenotype Score	PhenoAge	Brain Age	Gray Matter Volume	White Matter Volume	Brain Volume
Male	Blue space Subpopulation	**Coefficient**	0.029	0.001	-0.071	-0.062	-0.073	-0.082
		**SEM**	0.013	0.096	0.137	0.041	0.053	0.045
		***P* value**	0.029	0.989	0.605	0.130	0.170	0.071
		***FDR P* value**	0.111	0.996	0.761	0.300	0.363	0.208
	Rural-urban Fringe Subpopulation	**Coefficient**	0.041	0.078	0.083	-0.025	-0.017	-0.026
		**SEM**	0.005	0.034	0.045	0.013	0.017	0.015
		***P* value**	<0.001	0.020	0.063	0.059	0.315	0.074
		***FDR P* value**	<0.001	0.081	0.191	0.189	0.529	0.209
	Noise Subpopulation	**Coefficient**	0.035	-0.003	0.116	-0.064	-0.067	-0.079
		**SEM**	0.007	0.054	0.073	0.022	0.029	0.024
		***P* value**	<0.001	0.950	0.115	0.004	0.019	0.001
		***FDR P* value**	<0.001	0.974	0.274	0.020	0.079	0.006
	Air Pollution Subpopulation	**Coefficient**	0.051	0.014	0.096	-0.064	-0.053	-0.072
		**SEM**	0.006	0.042	0.059	0.018	0.023	0.020
		***P* value**	<0.001	0.737	0.105	<0.001	0.022	<0.001
		***FDR P* value**	<0.001	0.856	0.261	<0.001	0.087	<0.001
Female	Blue space Subpopulation	**Coefficient**	0.021	-0.009	0.095	-0.013	-0.015	-0.017
		**SEM**	0.013	0.087	0.127	0.037	0.048	0.041
		***P* value**	0.089	0.916	0.453	0.722	0.757	0.681
		***FDR P* value**	0.237	0.956	0.677	0.845	0.869	0.814
		**Coefficient**	0.048	0.085	0.026	-0.002	-0.004	-0.003
		**SEM**	0.004	0.031	0.043	0.013	0.016	0.014

**Rural-urban Fringe Subpopulation**	***P* value**	<0.001	0.006	0.548	0.905	0.819	0.827
	***FDR P* value**	<0.001	0.030	0.725	0.951	0.914	0.916
**Noise Subpopulation**	**Coefficient**	0.012	0.002	0.125	-0.025	-0.018	-0.026
	**SEM**	0.007	0.050	0.072	0.021	0.027	0.023
	***P* value**	0.107	0.968	0.080	0.235	0.509	0.257
	***FDR P* value**	0.263	0.985	0.217	0.448	0.706	0.471
**Air Pollution Subpopulation**	**Coefficient**	0.039	0.003	0.171	-0.043	-0.027	-0.043
	**SEM**	0.006	0.038	0.056	0.017	0.021	0.018
	***P* value**	<0.001	0.943	0.002	0.010	0.199	0.018
	***FDR P* value**	<0.001	0.973	0.012	0.047	0.401	0.076

Note: FDR, false discovery rate. SEM, standard error of mean. All models were adjusted for age, ethnicity, nSES, smoking status, BMI (category variable), alcohol intake frequency, regular exercise, healthy diet, history of CVD, and cancer at baseline. The volumes were normalized by the Z scores method. Two-sided P value of <0.05 was considered as statistically significant. Benjamini–Hochberg procedure was used to control the family-wise error rate in the subgroup analyses (n = 288). We set the green space subpopulation as the reference.

Supplementary Table 14. Subgroup analyses of associations of subpopulations with multidimensional aging metrics using linear regression models stratified by age.

Age			Frailty Phenotype Score	PhenoAge	Brain Age	Gray Matter Volume	White Matter Volume	Brain Volume
< 60 years old	Blue space Subpopulation	**Coefficient**	0.028	-0.027	0.075	-0.016	-0.061	-0.045
		**SEM**	0.012	0.125	0.125	0.038	0.043	0.041
		***P* value**	0.021	0.826	0.548	0.672	0.159	0.276
		***FDR P* value**	0.084	0.916	0.725	0.814	0.347	0.497
	Rural-urban Fringe Subpopulation	**Coefficient**	0.047	-0.026	0.023	0.002	-0.003	<0.001
		**SEM**	0.004	0.044	0.042	0.013	0.015	0.014
		***P* value**	<0.001	0.552	0.581	0.873	0.861	0.994
		***FDR P* value**	<0.001	0.726	0.747	0.941	0.932	0.996
	Noise Subpopulation	**Coefficient**	0.027	-0.272	0.081	-0.065	-0.052	-0.072
		**SEM**	0.007	0.069	0.069	0.021	0.024	0.022
		***P* value**	<0.001	<0.001	0.239	0.002	0.030	0.001
		***FDR P* value**	<0.001	<0.001	0.453	0.012	0.114	0.006
	Air Pollution Subpopulation	**Coefficient**	0.042	-0.293	0.038	-0.021	-0.017	-0.023
		**SEM**	0.005	0.053	0.054	0.016	0.019	0.018
		***P* value**	<0.001	<0.001	0.489	0.203	0.368	0.192
		***FDR P* value**	<0.001	<0.001	0.697	0.406	0.592	0.394
≥ 60 years old	Blue space Subpopulation	**Coefficient**	0.022	0.070	-0.048	-0.078	-0.010	-0.057
		**SEM**	0.014	0.111	0.152	0.050	0.064	0.053
		***P* value**	0.103	0.526	0.750	0.121	0.880	0.287
		***FDR P* value**	0.258	0.715	0.867	0.283	0.941	0.502
		**Coefficient**	0.042	0.092	0.061	-0.018	-0.011	-0.017
		**SEM**	0.005	0.039	0.049	0.017	0.021	0.017

**Rural-urban Fringe Subpopulation**	***P* value**	<0.001	0.020	0.212	0.288	0.611	0.318
	***FDR P* value**	<0.001	0.081	0.418	0.502	0.765	0.529
**Noise Subpopulation**	**Coefficient**	0.016	0.049	0.177	0.007	-0.015	-0.004
	**SEM**	0.008	0.065	0.084	0.028	0.036	0.030
	***P* value**	0.048	0.449	0.036	0.810	0.677	0.897
	***FDR P* value**	0.161	0.677	0.128	0.914	0.814	0.948
**Air Pollution Subpopulation**	**Coefficient**	0.047	-0.059	0.162	-0.040	-0.046	-0.052
	**SEM**	0.006	0.051	0.068	0.023	0.029	0.024
	***P* value**	<0.001	0.249	0.017	0.078	0.114	0.031
	***FDR P* value**	<0.001	0.463	0.074	0.216	0.274	0.114

Note: FDR, false discovery rate. SEM, standard error of mean. All models were adjusted for sex, ethnicity, nSES, smoking status, BMI (category variable), alcohol intake frequency, regular exercise, healthy diet, history of CVD, and cancer at baseline. The volumes were normalized by the Z scores method. Two-sided P value of <0.05 was considered as statistically significant. Benjamini–Hochberg procedure was used to control the family-wise error rate in the subgroup analyses (n = 288). We set the green space subpopulation as reference.

Supplementary Table 15. Subgroup analyses of associations of subpopulations with multidimensional aging metrics using linear regression models stratified by smoking status.

Smoking status			Frailty Phenotype Score	PhenoAge	Brain Age	Gray Matter Volume	White Matter Volume	Brain Volume
Never smoker	Blue space Subpopulation	**Coefficient**	0.025	0.049	0.103	-0.072	-0.091	-0.098
		**SEM**	0.012	0.086	0.118	0.035	0.045	0.039
		***P* value**	0.036	0.573	0.384	0.041	0.044	0.012
		***FDR P* value**	0.128	0.740	0.601	0.144	0.149	0.054
	Rural-urban Fringe Subpopulation	**Coefficient**	0.045	0.070	0.071	-0.016	-0.005	-0.014
		**SEM**	0.004	0.030	0.039	0.012	0.015	0.013
		***P* value**	<0.001	0.018	0.072	0.162	0.719	0.289
		***FDR P* value**	<0.001	0.076	0.208	0.351	0.845	0.502
	Noise Subpopulation	**Coefficient**	0.032	0.048	0.107	-0.077	-0.027	-0.066
		**SEM**	0.007	0.049	0.066	0.020	0.025	0.022
		***P* value**	<0.001	0.330	0.108	<0.001	0.291	0.003
		***FDR P* value**	<0.001	0.537	0.264	<0.001	0.502	0.016
	Air Pollution Subpopulation	**Coefficient**	0.057	0.095	0.140	-0.058	-0.036	-0.059
		**SEM**	0.005	0.038	0.053	0.016	0.020	0.017
		***P* value**	<0.001	0.013	0.008	<0.001	0.072	0.001
		***FDR P* value**	<0.001	0.058	0.038	<0.001	0.208	0.006
Ever smoker	Blue space Subpopulation	**Coefficient**	0.031	-0.079	-0.245	0.061	0.026	0.055
		**SEM**	0.015	0.109	0.164	0.048	0.063	0.053
		***P* value**	0.042	0.466	0.135	0.209	0.673	0.301
		***FDR P* value**	0.146	0.688	0.309	0.415	0.814	0.516
		**Coefficient**	0.043	0.140	0.030	-0.001	-0.030	-0.017

	**Rural-urban Fringe Subpopulation**	**SEM**	0.005	0.039	0.054	0.016	0.021	0.018
		***P* value**	<0.001	<0.001	0.584	0.950	0.153	0.327
		***FDR P* value**	<0.001	<0.001	0.748	0.974	0.339	0.535
	**Noise Subpopulation**	**Coefficient**	0.012	-0.064	0.116	0.010	-0.067	-0.031
		**SEM**	0.009	0.063	0.088	0.026	0.034	0.029
		***P* value**	0.151	0.307	0.185	0.712	0.049	0.282
		***FDR P* value**	0.337	0.523	0.386	0.845	0.162	0.501
	**Air Pollution Subpopulation**	**Coefficient**	0.037	-0.018	0.090	-0.028	-0.046	-0.044
		**SEM**	0.007	0.049	0.070	0.021	0.027	0.023
		***P* value**	<0.001	0.718	0.199	0.181	0.092	0.055
		***FDR P* value**	<0.001	0.845	0.401	0.383	0.241	0.178
**Current smoker**	**Blue space Subpopulation**	**Coefficient**	0.007	-0.002	0.697	-0.156	0.131	-0.029
		**SEM**	0.034	0.227	0.388	0.118	0.150	0.129
		***P* value**	0.846	0.992	0.073	0.187	0.382	0.819
		***FDR P* value**	0.926	0.996	0.208	0.387	0.601	0.914
	**Rural-urban Fringe Subpopulation**	**Coefficient**	0.059	-0.101	-0.013	-0.038	0.083	0.022
		**SEM**	0.013	0.085	0.141	0.042	0.053	0.046
		***P* value**	<0.001	0.235	0.927	0.371	0.118	0.636
		***FDR P* value**	<0.001	0.448	0.961	0.592	0.279	0.786
	**Noise Subpopulation**	**Coefficient**	0.021	-0.100	0.158	-0.040	-0.025	-0.040
		**SEM**	0.018	0.121	0.213	0.063	0.080	0.069
		***P* value**	0.251	0.408	0.457	0.531	0.754	0.562
		***FDR P* value**	0.463	0.632	0.678	0.718	0.869	0.736
		**Coefficient**	0.024	-0.286	0.230	-0.127	-0.009	-0.088

**Air Pollution Subpopulation**	**SEM**	0.014	0.096	0.165	0.049	0.063	0.054
	***P* value**	0.092	0.003	0.165	0.011	0.882	0.102
	***FDR P* value**	0.241	0.016	0.355	0.051	0.941	0.258

Note: FDR, false discovery rate. SEM, standard error of mean. All models were adjusted for age, sex, ethnicity, nSES, BMI (category variable), alcohol intake frequency, regular exercise, healthy diet, history of CVD, and cancer at baseline. The volumes were normalized by the Z scores method. Two-sided P value of <0.05 was considered as statistically significant. Benjamini–Hochberg procedure was used to control the family-wise error rate in the subgroup analyses (n = 288). We set the green space subpopulation as reference.

Supplementary Table 16. Subgroup analyses of associations of subpopulations with multidimensional aging metrics using linear regression models stratified by alcohol intake frequency.

Alcohol intake frequency			Frailty Phenotype Score	PhenoAge	Brain Age	Gray Matter Volume	White Matter Volume	Brain Volume
Never or special occasions	Blue space Subpopulation	**Coefficient**	0.027	0.130	0.180	-0.020	-0.119	-0.080
		**SEM**	0.027	0.186	0.272	0.087	0.110	0.095
		***P* value**	0.317	0.487	0.507	0.814	0.279	0.402
		***FDR P* value**	0.529	0.697	0.706	0.914	0.499	0.626
	Rural-urban Fringe Subpopulation	**Coefficient**	0.060	0.072	-0.133	0.006	-0.020	-0.008
		**SEM**	0.009	0.065	0.091	0.028	0.035	0.031
		***P* value**	<0.001	0.268	0.142	0.842	0.570	0.805
		***FDR P* value**	<0.001	0.485	0.322	0.926	0.740	0.913
	Noise Subpopulation	**Coefficient**	0.043	-0.012	0.094	-0.037	-0.036	-0.044
		**SEM**	0.014	0.097	0.137	0.042	0.054	0.046
		***P* value**	0.002	0.899	0.491	0.384	0.499	0.339
		***FDR P* value**	0.012	0.948	0.697	0.601	0.701	0.548
	Air Pollution Subpopulation	**Coefficient**	0.068	0.059	-0.047	-0.045	-0.027	-0.045
		**SEM**	0.011	0.077	0.112	0.035	0.044	0.038
		***P* value**	<0.001	0.443	0.673	0.193	0.542	0.241
		***FDR P* value**	<0.001	0.671	0.814	0.394	0.725	0.454
One to three times per	Blue space Subpopulation	**Coefficient**	0.031	0.056	-0.214	-0.004	0.075	0.039
		**SEM**	0.029	0.211	0.305	0.086	0.111	0.095
		***P* value**	0.290	0.792	0.483	0.965	0.499	0.681
		***FDR P* value**	0.502	0.905	0.697	0.985	0.701	0.814
		**Coefficient**	0.047	0.136	0.049	-0.021	-0.007	-0.018
		**SEM**	0.010	0.073	0.098	0.028	0.036	0.031

	**Rural-urban Fringe Subpopulation**	***P* value**	<0.001	0.062	0.616	0.454	0.851	0.571
		***FDR P* value**	<0.001	0.191	0.768	0.677	0.928	0.740
	**Noise Subpopulation**	**Coefficient**	0.024	0.012	0.014	-0.086	-0.012	-0.063
		**SEM**	0.016	0.117	0.156	0.046	0.059	0.051
		***P* value**	0.147	0.915	0.928	0.061	0.845	0.216
		***FDR P* value**	0.331	0.956	0.961	0.191	0.926	0.423
	**Air Pollution Subpopulation**	**Coefficient**	0.049	0.158	0.067	-0.009	0.057	0.026
		**SEM**	0.013	0.090	0.125	0.036	0.046	0.040
		***P* value**	<0.001	0.080	0.592	0.797	0.218	0.519
		***FDR P* value**	<0.001	0.217	0.753	0.907	0.424	0.708
**One to four times per**	**Blue space Subpopulation**	**Coefficient**	0.025	-0.032	0.097	-0.040	-0.077	-0.069
		**SEM**	0.012	0.088	0.123	0.036	0.046	0.040
		***P* value**	0.043	0.713	0.431	0.265	0.095	0.081
		***FDR P* value**	0.147	0.845	0.661	0.483	0.246	0.218
	**Rural-urban Fringe Subpopulation**	**Coefficient**	0.040	0.035	0.075	-0.011	-0.011	-0.013
		**SEM**	0.004	0.030	0.042	0.012	0.016	0.014
		***P* value**	<0.001	0.249	0.073	0.372	0.484	0.326
		***FDR P* value**	<0.001	0.463	0.208	0.592	0.697	0.535
	**Noise Subpopulation**	**Coefficient**	0.024	-0.035	0.163	-0.053	-0.073	-0.075
		**SEM**	0.007	0.050	0.072	0.021	0.027	0.023
		***P* value**	0.001	0.488	0.024	0.012	0.007	0.001
		***FDR P* value**	0.006	0.697	0.093	0.054	0.034	0.006
	**Air Pollution Subpopulation**	**Coefficient**	0.042	-0.076	0.188	-0.065	-0.038	-0.064
		**SEM**	0.005	0.039	0.056	0.017	0.021	0.018

Daily or almost daily		***P* value**	<0.001	0.050	0.001	<0.001	0.077	0.001
		***FDR P* value**	<0.001	0.164	0.006	<0.001	0.215	0.006
	**Blue space Subpopulation**	**Coefficient**	0.027	-0.074	-0.144	-0.038	0.042	-0.002
		**SEM**	0.017	0.124	0.201	0.062	0.078	0.067
		***P* value**	0.103	0.549	0.473	0.535	0.595	0.977
		***FDR P* value**	0.258	0.725	0.693	0.720	0.753	0.991
	**Rural-urban Fringe Subpopulation**	**Coefficient**	0.051	0.178	0.099	-0.023	<0.001	-0.015
		**SEM**	0.006	0.046	0.064	0.020	0.025	0.021
		***P* value**	<0.001	<0.001	0.125	0.235	0.996	0.474
		***FDR P* value**	<0.001	<0.001	0.290	0.448	0.996	0.693
	**Noise Subpopulation**	**Coefficient**	0.013	0.073	0.050	-0.005	0.014	0.005
		**SEM**	0.010	0.074	0.104	0.032	0.041	0.035
		***P* value**	0.184	0.325	0.633	0.879	0.727	0.892
		***FDR P* value**	0.386	0.535	0.786	0.941	0.848	0.948
	**Air Pollution Subpopulation**	**Coefficient**	0.039	0.083	0.134	-0.047	-0.094	-0.083
		**SEM**	0.008	0.058	0.085	0.026	0.033	0.028
		***P* value**	<0.001	0.156	0.114	0.071	0.004	0.003
		***FDR P* value**	<0.001	0.343	0.274	0.208	0.020	0.016

Note: FDR, false discovery rate. SEM, standard error of mean. All models were adjusted for age, sex, ethnicity, nSES, BMI (category variable), smoking status regular exercise, healthy diet, history of CVD, and cancer at baseline. The volumes were normalized by the Z scores method. Two-sided P value of <0.05 was considered as statistically significant. Benjamini–Hochberg procedure was used to control the family-wise error rate in the subgroup analyses (n = 288). We set the green space subpopulation as reference.

Supplementary Table 17. Dictionary ID of variates.

Variates	FieldID	Measure time	Participants number
**Environmental exposure**			
Nitrogen dioxide air pollution (Average of 2005, 2006, 2007, 2010)	24016	2005	494,979
	24017	2006	494,979
	24018	2007	494,979
	24003	2010	494,979
Particulate matter air pollution (pm10; Average of 2007, 2010)	24019	2007	493,881
	24005	2010	461,060
Nitrogen oxides air pollution; 2010	24004	2010	494,979
Particulate matter air pollution (pm2.5); 2010	24006	2010	461,060
Particulate matter air pollution 2.5-10um; 2010	24008	2010	461,060
Average daytime sound level of noise pollution	24020	2015	494,979
Average evening sound level of noise pollution	24021	2015	494979

Average night-time sound level of noise pollution	24022	2015	494,979
Average 16-hour sound level of noise pollution	24023	2015	494979
Average 24-hour sound level of noise pollution	24024	2015	494,979
Greenspace percentage, buffer 1000m	24500	2006-2010	440,734
		2012-2013	20,321
Blue percentage, buffer 1000m	24502	2006-2010	440,734
		2012-2013	20,321
Greenspace percentage, buffer 300m	24503	2006-2010	440,734
		2012-2013	20,321
Blue percentage, buffer 300m	24505	2006-2010	440,734
		2012-2013	20,321
**PhenoAge**			
Albumin	30600	2006-2010	429,951
		2012-2013	15,692
Creatinine	30700	2006-2010	469,221
		2012-2013	17,840
Glucose	30740	2006-2010	429,443
		2012-2013	15,683
Lymphocyte percentage	30180	2006-2010	477,149
		2012-2013	19,398
		2014+	5,855
Mean corpuscular volume	30040	2006-2010	477,149

		2012-2013	19,398
		2014+	5,855
Red blood cell (erythrocyte) distribution width	30070	2006-2010	478,030
		2012-2013	19,409
		2014+	5,862
Alkaline phosphatase	30610	2006-2010	469,466
		2012-13	17,867
White blood cell (leukocyte) count	30000	2006-2010	478,027
		2012-2013	19,408
		2014+	5,862
C-reactive protein	30710	2006-2010	468,432
		2012-2013	17,834
**Frailty Phenotype Score**			
Weight Loss	2306	2006-2010	501,435
		2012-2013	20,333
		2014+	75,333
		2019+	6,946
Exhaustion	2080	2006-2010	501,435
		2012-2013	20,333
		2014+	75,333
		2019+	6,946
Walking Speed	924	2006-2010	501,435
		2012-2013	20,333
		2014+	75,333
		2019+	6,946
Grip strength	46	2006-2010	498,944

		2012-2013	20,196
		2014+	73,660
		2019+	6,724
	47	2006-2010	499,011
		2012-2013	20,211
		2014+	73,660
		2019+	6,724
	6164	2006-2010	496,054
		2012-2013	20,286
		2014+	75,277
		2019+	6,946
	2624	2006-2010	205,381
		2012-2013	8,157
		2014+	35,645
		2019+	3,490
Physical activity	1011	2006-2010	496,054
		2012-2013	20,286
		2014+	75,277
		2019+	6,946
	3637	2006-2010	236,024
		2012-2013	10,469
		2014+	40,264
		2019+	3,816
	971	2006-2010	350,838
		2012-2013	15,107
		2014+	61,660

		2019+	5,934
	991	2006-2010	50,053
		2012-2013	2,222
		2014+	8,771
		2019+	848
**Brain IDPs**			
Total brain volume	25009	2014+	46,398
		2019+	4,784
Total GM volume	25005	2014+	46,398
		2019+	4,784
Total WM volume	25007	2014+	46,398
		2019+	4,784
Superior frontal gyri	25786	2014+	46,393
		2019+	4,784
	25787	2014+	46,393
		2019+	4,784
Inferior frontal gyri	25790	2014+	46,393
		2019+	4,784
	25791	2014+	46,393
		2019+	4,784
	25792	2014+	46,393
		2019+	4,784
	25793	2014+	46,393
		2019+	4,784
Middle frontal gyri	25788	2014+	46,393

		2019+	4,784
	25789	2014+	46,393
		2019+	4,784
Supplementary motor cortex	25832	2014+	46,393
		2019+	4,784
	25833	2014+	46,393
		2019+	4,784
Precentral gyrus	25794	2014+	46,393
		2019+	4,784
	25795	2014+	46,393
		2019+	4,784
Postcentral gyrus	25814	2014+	46,393
		2019+	4,784
	25815	2014+	46,393
		2019+	4,784
Precuneus	25842	2014+	46,393
		2019+	4,784
	25843	2014+	46,393
		2019+	4,784
Superior parietal lobe	25816	2014+	46,393
		2019+	4,784
	25817	2014+	46,393
		2019+	4,784
Parahippocampal gyrus	25848	2014+	46,393
		2019+	4,784

	25849	2014+	46,393
		2019+	4,784
	25850	2014+	46,393
		2019+	4,784
	25851	2014+	46,393
		2019+	4,784
	25802	2014+	46,393
		2019+	4,784
	25803	2014+	46,393
		2019+	4,784
	25804	2014+	46,393
		2019+	4,784
Middle temporal gyrus	25805	2014+	46,393
		2019+	4,784
	25806	2014+	46,393
		2019+	4,784
	25807	2014+	46,393
		2019+	4,784
	25808	2014+	46,393
		2019+	4,784
	25809	2014+	46,393
		2019+	4,784
Inferior temporal gyrus	25810	2014+	46,393
		2019+	4,784
	25811	2014+	46,393
		2019+	4,784

	25812	2014+	46,393
		2019+	4,784
	25813	2014+	46,393
		2019+	4,784
Hippocampus	25886	2014+	46,393
		2019+	4,784
	25887	2014+	46,393
		2019+	4,784
Putamen	25882	2014+	46,393
		2019+	4,784
	25883	2014+	46,393
		2019+	4,784
Thalamus	25011	2014+	46,381
		2019+	4,784
	25012	2014+	46,381
		2019+	4,784
Caudate	25013	2014+	46,381
		2019+	4,784
	25014	2014+	46,381
		2019+	4,784
Amygdala	25021	2014+	46,381
		2019+	4,784
	25022	2014+	46,381
		2019+	4,784
**Cognition**			

Pairs matching task	399	2006-2010	497,732
		2012-2013	20,333
		2014+	70,923
		2019+	6,700
Reaction time task	20023	2006-2010	496,533
		2012-2013	20,253
		2014+	70,551
		2019+	6,599
Fluid intelligence test	20016	2006-2010	165,408
		2012-2013	20,109
		2014+	69,804
		2019+	6,566
Maximum digits remembered correctly	4282	2006-2010	51,793
		2014+	43,197
		2019+	5,097
Symbol digit substitution task	20195	2014+	118,433
		2021	177,258
Trail making A tasks	20156	2014+	104,000
		2021	175,406
Trail making B tasks	20157	2014+	103,998
		2021	152,788

Note: The method of the ageing metric has been explained in detail in the manuscript. The details of 365 structural magnetic resonance imaging features used to construct the brain age were provided in previous research[8].

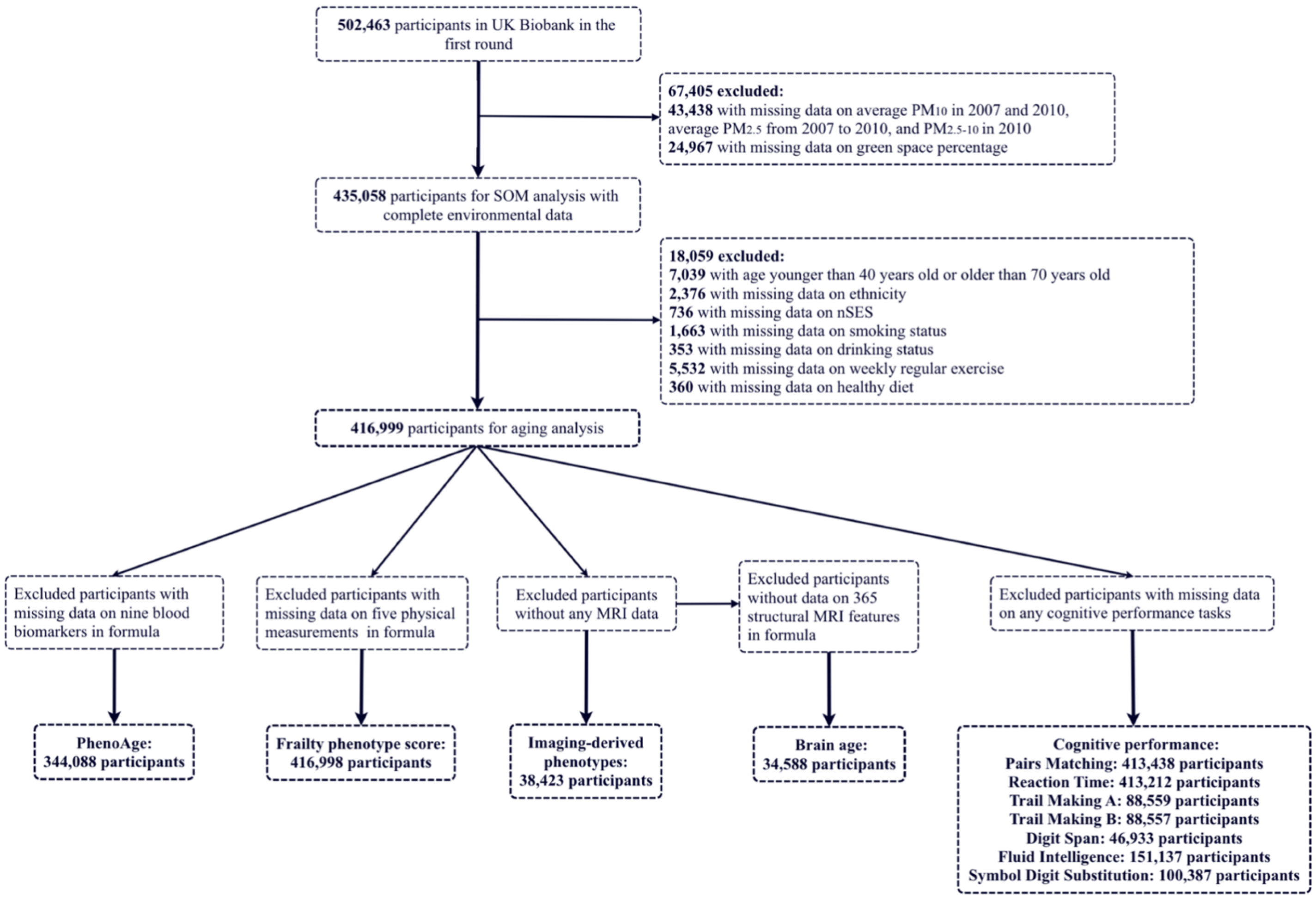

Supplemeary Figure 1. Flow chart of selecting analytic samples from the UK Biobank

Note: SOM, self-organizing map; PhenoAge, phenotypic age; MRI, magnetic resonance imaging; The number of participants with data on imaging-derived phenotypes of specific brain regions varied. 38,423 participants in the figure had data on imaging-derived phenotypes of all the included brain regions.

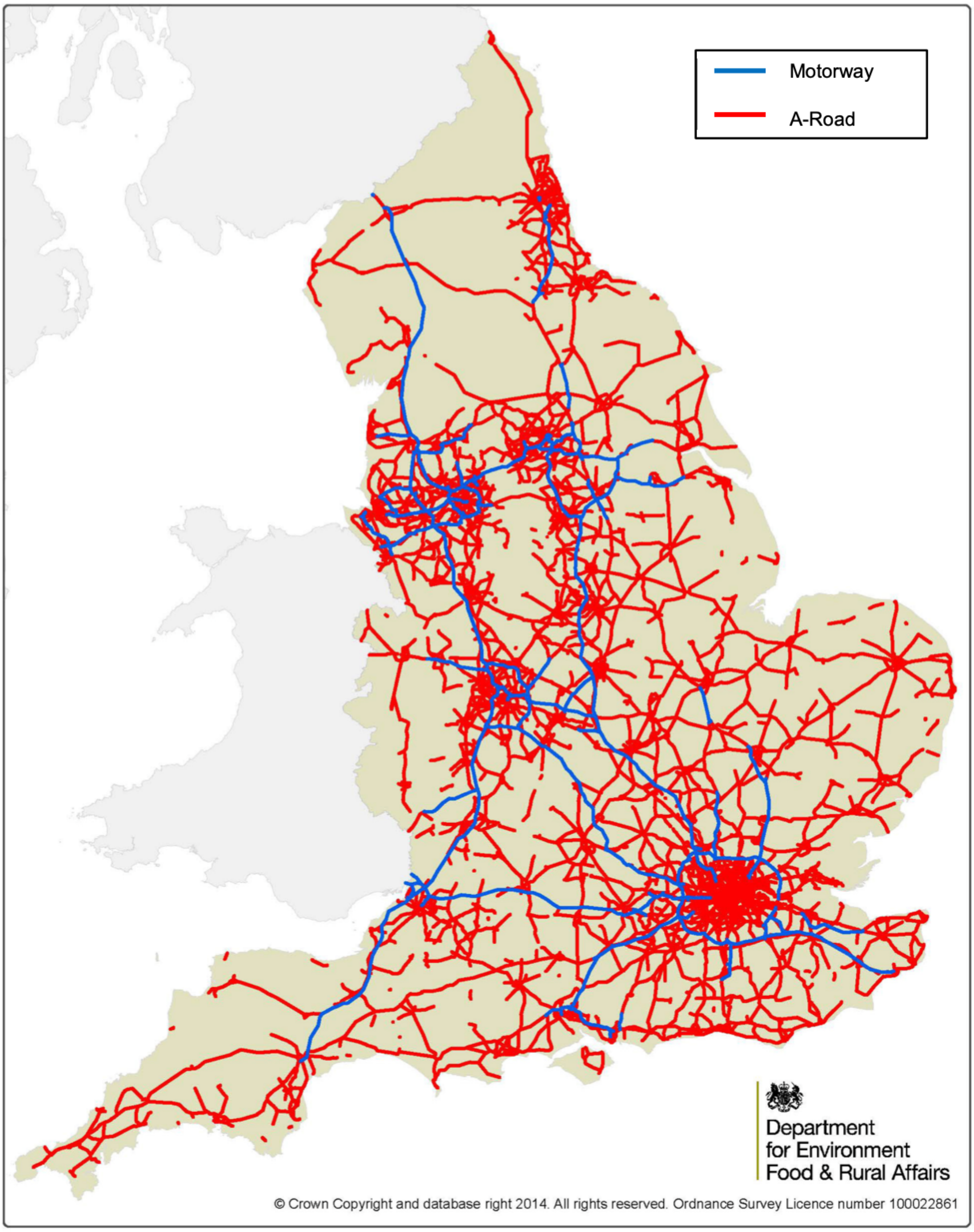

Supplementary Figure 2. Major roads in England 2017.

Note: The figure can be downloaded from https://www.gov.uk/government/publications/strategic-noise-mapping-2019.

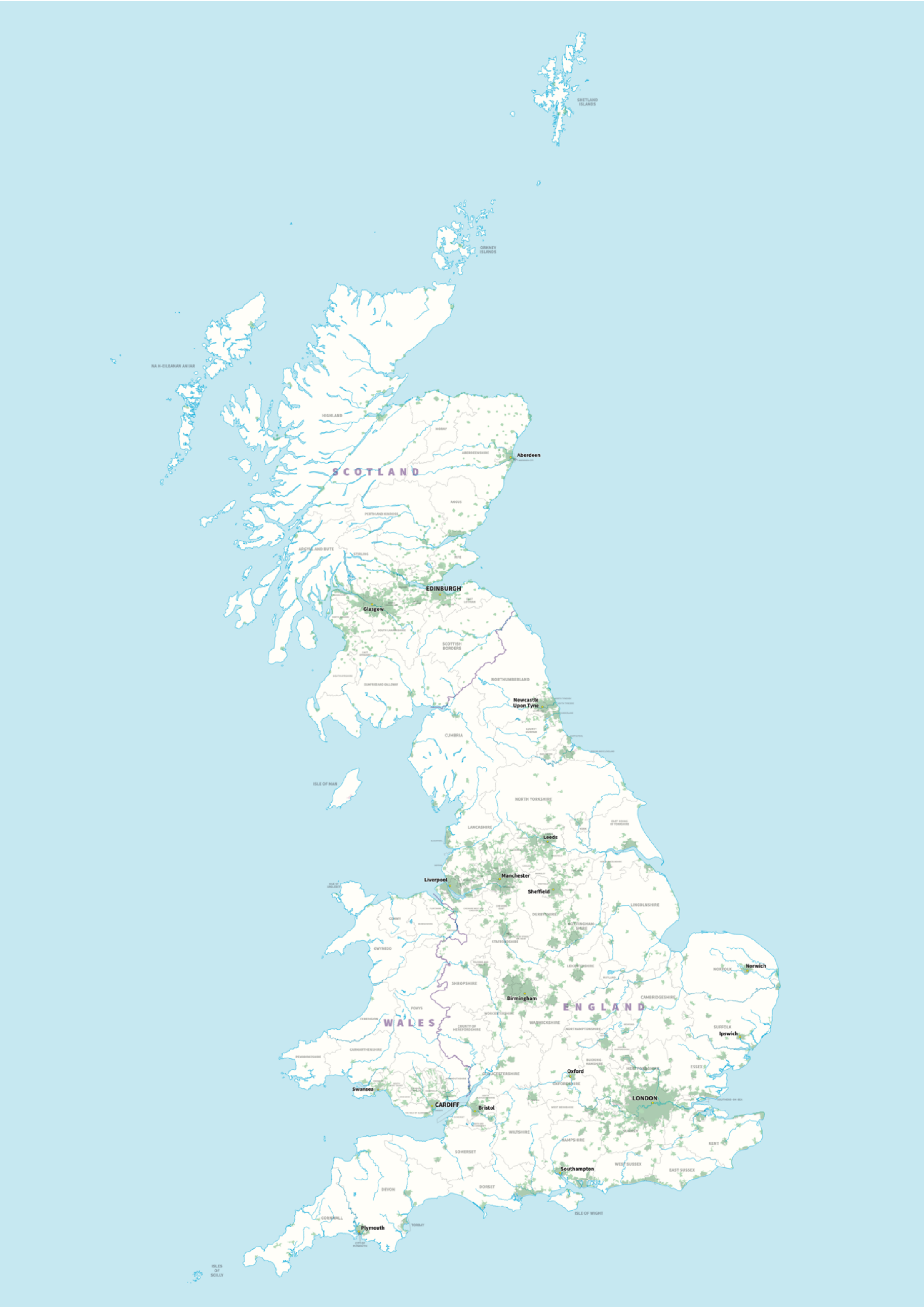

Supplementary Figure 3. OS MasterMap rivers across England, Scotland and Wales

Note: The figure can be downloaded from https://www.ordnancesurvey.co.uk/docs/technical-specifications/os-mastermap-greenspace-layer-technical-specification.pdf.

Reference

1 Townsend, P., Phillimore, P., & Beattie, A. *Health and Deprivation: Inequality and the North*. (Routledge, 1988).

2 Niedzwiedz, C. L. *et al.* Ethnic and socioeconomic differences in SARS-CoV-2 infection: prospective cohort study using UK Biobank. *BMC Med* **18**, 160 (2020). https://doi.org:10.1186/s12916-020-01640-8

3 Hanlon, P. *et al.* Frailty and pre-frailty in middle-aged and older adults and its association with multimorbidity and mortality: a prospective analysis of 493 737 UK Biobank participants. *The Lancet. Public health* **3**, e323-e332 (2018). https://doi.org:10.1016/s2468-2667(18)30091-4

4 Chudasama, Y. V. *et al.* Healthy lifestyle and life expectancy in people with multimorbidity in the UK Biobank: A longitudinal cohort study. *PLoS Med* **17**, e1003332 (2020). https://doi.org:10.1371/journal.pmed.1003332

5 Mozaffarian, D. Dietary and Policy Priorities for Cardiovascular Disease, Diabetes, and Obesity: A Comprehensive Review. *Circulation* **133**, 187-225 (2016). https://doi.org:10.1161/circulationaha.115.018585

6 Said, M. A., Verweij, N. & van der Harst, P. Associations of Combined Genetic and Lifestyle Risks With Incident Cardiovascular Disease and Diabetes in the UK Biobank Study. *JAMA Cardiol* **3**, 693-702 (2018). https://doi.org:10.1001/jamacardio.2018.1717

7 Care, D. o. H. S. *Guidance: the NHS Constitution for Englan*, <http://www.gov.uk/government/publications/the-nhs-constitution-for-england/the-nhs-constitution-for-england> (2015).

8 Vidal-Pineiro, D. *et al.* Individual variations in 'brain age' relate to early-life factors more than to longitudinal brain change. *eLife* **10**, e69995 (2021). https://doi.org:10.7554/eLife.69995
