# Supplementary material for: Heterogeneous associations of multiplexed environmental factors and multidimensional aging metrics
Source: Nat Commun. 2024 Jun 10;15:4921. doi: 10.1038/s41467-024-49283-0 (PMC11164970; doi:10.1038/s41467-024-49283-0)
Supplement: Supplementary file 2 — Reporting Summary [file 41467_2024_49283_MOESM2_ESM.pdf]

nature portfolio

Corresponding author(s): Zuyun Liu

Last updated by author(s): 5/13/2024

Reporting Summary

Nature Portfolio wishes to improve the reproducibility of the work that we publish. This form provides structure for consistency and transparency in reporting. For further information on Nature Portfolio policies, see our Editorial Policies and the Editorial Policy Checklist.

Statistics

For all statistical analyses, confirm that the following items are present in the figure legend, table legend, main text, or Methods section.

n/a	Confirmed	
☐	☒	The exact sample size (*n*) for each experimental group/condition, given as a discrete number and unit of measurement
☐	☒	A statement on whether measurements were taken from distinct samples or whether the same sample was measured repeatedly
☐	☒	The statistical test(s) used AND whether they are one- or two-sided<br>*Only common tests should be described solely by name; describe more complex techniques in the Methods section.*
☐	☒	A description of all covariates tested
☐	☒	A description of any assumptions or corrections, such as tests of normality and adjustment for multiple comparisons
☐	☒	A full description of the statistical parameters including central tendency (e.g. means) or other basic estimates (e.g. regression coefficient) AND variation (e.g. standard deviation) or associated estimates of uncertainty (e.g. confidence intervals)
☐	☒	For null hypothesis testing, the test statistic (e.g. *F*, *t*, *r*) with confidence intervals, effect sizes, degrees of freedom and *P* value noted<br>*Give P values as exact values whenever suitable.*
☒	☐	For Bayesian analysis, information on the choice of priors and Markov chain Monte Carlo settings
☐	☒	For hierarchical and complex designs, identification of the appropriate level for tests and full reporting of outcomes
☐	☒	Estimates of effect sizes (e.g. Cohen's *d*, Pearson's *r*), indicating how they were calculated

Our web collection on statistics for biologists contains articles on many of the points above.

Software and code

Policy information about availability of computer code

Data collection	No software was involved in data collection (data used is all directly available from UK Biobank, as described in detail in the paper)
Data analysis	R (version 4.2.1) packages:<br>glmnet package (version 4.1-8) was used to fit linear regression models;<br>gWQS package (version 3.0.5) was used to perform the weighted quantile sum regression;<br>kohonen package (version 3.0.12) was used to perform the self-organizing maps analysis;<br>stats package (version 4.4.0) was used to perform the Benjamini–Hochberg procedure.

For manuscripts utilizing custom algorithms or software that are central to the research but not yet described in published literature, software must be made available to editors and reviewers. We strongly encourage code deposition in a community repository (e.g. GitHub). See the Nature Portfolio guidelines for submitting code & software for further information.

Data

Policy information about availability of data

All manuscripts must include a data availability statement. This statement should provide the following information, where applicable:

- Accession codes, unique identifiers, or web links for publicly available datasets
- A description of any restrictions on data availability
- For clinical datasets or third party data, please ensure that the statement adheres to our policy

The data used in the present study are available from UKB with restrictions applied. Data were used under license and are thus not publicly available. Access to the UKB data can be requested through a standard protocol (https://www.ukbiobank.ac.uk/register-apply/). It should be noted that, the data of the multiplexed environmental factors are directly obtained from UKB. We didn’t performed models (e.g., the land use regression model) to construct any environmental factors and we explained how UKB construct environmental factors clearly in the Methods section.

Research involving human participants, their data, or biological material

Policy information about studies with human participants or human data. See also policy information about sex, gender (identity/presentation), and sexual orientation and race, ethnicity and racism.

Reporting on sex and gender	We took sex into considerations in our study and our findings could apply to both male and female. Sex (Field ID 31) in the UK Biobank was determined based on self-reporting data via questionnaire, and included participants for each aging metric gave written informed consent for sharing of individual-level data.
Reporting on race, ethnicity, or other socially relevant groupings	Ethnicity was used in our study as one of covariates considered for the aging acceleration might differentiate among different ethnics. Moreover, covariates were selected based on previous related studies, including sex (Field ID 31), ethnicity, social socioeconomic status and individual socioeconomic status (iSES) at baseline. An overall iSES variable was created by latent class analysis based on three individual socioeconomic factors, household income (Field ID 738), education level (Field ID 6138), and employment status (Field ID 6142). According to the item-response probabilities, three latent classes were identified, representing a high, medium, and low SES (details are reported in the supplementary file).
Population characteristics	The number of participants in the phenotypic age and frailty analyses was relatively large, with 344,088 and 416,998 people, respectively. 34,588 participants who were relatively younger, with a mean age of 55.46 ± 7.37 years, were included in the analysis of brain age. We calculated descriptive statistics as mean (SD) for continuous variables and number (percentage) for categorical variables.
Recruitment	The UKB enrolled the participants aged 40-69 years between 2006 and 2010 for baseline assessments in 22 centers across the UK. The assessment visits comprised interviews and questionnaires covering lifestyles and health conditions, physical measures, biological samples, imaging, and genotyping. The database is linked to national health datasets, including primary care, hospital inpatient, death, and cancer registration data.
Ethics oversight	UK Biobank has received ethical approval from the North West Multi-centre Research Ethics Committee (MREC, https://www.ukbiobank.ac.uk/learn-more-about-uk-biobank/about-us/ethics), and informed consent through electronic signature was obtained from study participants. This study utilized the UK Biobank Resource under application number 61856.

Note that full information on the approval of the study protocol must also be provided in the manuscript.

Field-specific reporting

Please select the one below that is the best fit for your research. If you are not sure, read the appropriate sections before making your selection.

[x] Life sciences [ ] Behavioural & social sciences [ ] Ecological, evolutionary & environmental sciences

For a reference copy of the document with all sections, see nature.com/documents/nr-reporting-summary-flat.pdf

Life sciences study design

All studies must disclose on these points even when the disclosure is negative.

Sample size	No statistical methods were used to predetermine sample sizes. All current available sample in the UK Biobank were included. The number of participants in the phenotypic age and frailty analyses was 344,088 and 416,998 people, respectively. 34,588 participants were included in the analysis of brain age. We calculated descriptive statistics as mean (SD) for continuous variables and number (percentage) for categorical variables. Note that the UK Biobank is one of the largest datasets worldwide.
Data exclusions	Participants with missing data on environmental exposures and those without available data on covariates were excluded.
Replication	All available data were used to maximize statistical power of the analysis therefore we did not repeat the analysis.
Randomization	Covariates were selected based on previous related studies, including age, sex, ethnicity, socioeconomic status (SES), smoking status, body mass index (BMI), alcohol intake frequency, regular exercise, healthy diet, history of cancer and cardiovascular disease (CVD) at baseline.

Blinding	Blinding was not applicable to this study as this study is observational.

Reporting for specific materials, systems and methods

We require information from authors about some types of materials, experimental systems and methods used in many studies. Here, indicate whether each material, system or method listed is relevant to your study. If you are not sure if a list item applies to your research, read the appropriate section before selecting a response.

Materials & experimental systems

n/a	Involved in the study
☒	☐ Antibodies
☒	☐ Eukaryotic cell lines
☒	☐ Palaeontology and archaeology
☒	☐ Animals and other organisms
☒	☐ Clinical data
☒	☐ Dual use research of concern
☒	☐ Plants

Methods

n/a	Involved in the study
☒	☐ ChIP-seq
☒	☐ Flow cytometry
☐	☒ MRI-based neuroimaging

Plants

Seed stocks	/
Novel plant genotypes	/
Authentication	/

Magnetic resonance imaging

Experimental design

Design type	Structural MRI
Design specifications	UK Biobank designed the imaging acquisition protocals including 6 modalities, covering structural, diffusion and functional imaging. The collection order is T1-weighted structural image, resting-state functional MRI, task functional MRI, T2-weighted FLAIR structural image, Diffution MRI and susceptibility-weighted imaging. T1-weighted structural image was acquired using straight sagittal orientation for 5 minutes.
Behavioral performance measures	None

Acquisition

Imaging type(s)	T1-weighted structural imaging
Field strength	3T
Sequence & imaging parameters	The EPI-based acquisitions utilize simultaneous multi-slice (multiband) acceleration. Biobank uses pulse sequences and reconstruction code from the Center for Magnetic Resonance Research (CMRR), University of Minnesota https://www.cmr.umn.edu/multiband. The resolution is 1x1x1 mm and field of view is 208x256x256 matrix. Straight sagittal orientation is used. TR and TE are 2000ms and 2.01ms respectively. The flip angle is 8 deg. Detailed sequence and imaging parameters are openly available here: https://biobank.ndph.ox.ac.uk/showcase/showcase/docs/ brain_mri.pdf
Area of acquisition	Whole brain

Diffusion MRI ☐ Used ☒ Not used

Preprocessing

Preprocessing software	T1 images were processed with FreeSurfer; surface templates were used to extract imaging-derived phenotypes referred to as atlas regions' surface volume. Subcortical regions were extracted via FreeSurfer.s aseg tool.
Normalization	see above
Normalization template	fsaverage
Noise and artifact removal	Prior to any analysis, we tentatively removed observations for which > 5% of the features fell above or below 5 SD from the sample mean. The application of this arbitrary high threshold led to the removal of 10 observations. We considered these MRI data to be extreme outliers and likely to be artifactual and/or contaminated by important sources of noise.
Volume censoring	see above

Statistical modeling & inference

Model type and settings	WQS and linear regression models were used to examine the associations of brain indicators with multiplexed environmental factors. the individuals' brain age was estimated using machine learning method, gradient tree boosting as implemented in XGBoost (https://xgboost.readthedocs.io) and optimized using 10-fold cross-validation and a randomized hyperparameter search. The volumes were converted to Z scores.
Effect(s) tested	Mann–Whitney U was used to examine the differences in continuous variables. Wald tests were utilized to derive the two-sided p value for linear regression models.

Specify type of analysis: [x] Whole brain [ ] ROI-based [ ] Both

Statistic type for inference (See Eklund et al. 2016)	voxel-wise association, voxel-wise Bonferroni correction
Correction	Bonferroni

Models & analysis

n/a	Involved in the study
[x]	[ ] Functional and/or effective connectivity
[x]	[ ] Graph analysis
[x]	[ ] Multivariate modeling or predictive analysis
