# Supplementary material for: Heterogeneous associations of multiplexed environmental factors and multidimensional aging metrics
Source: Nat Commun. 2024 Jun 10;15:4921. doi: 10.1038/s41467-024-49283-0 (PMC11164970; doi:10.1038/s41467-024-49283-0)
Supplement: Supplementary file 3 — Peer Review File [file 41467_2024_49283_MOESM3_ESM.pdf]

nature portfolio

Peer Review File

Heterogeneous associations of multiplexed environmental factors and multidimensional aging metrics: A proof of concept study

Open Access This file is licensed under a Creative Commons Attribution 4.0 International License, which permits use, sharing, adaptation, distribution and reproduction in any medium or format, as long as you give appropriate credit to the original author(s) and the source, provide a link to the Creative Commons license, and indicate if changes were made. In the cases where the authors are anonymous, such as is the case for the reports of anonymous peer reviewers, author attribution should be to 'Anonymous Referee' followed by a clear attribution to the source work. The images or other third party material in this file are included in the article's Creative Commons license, unless indicated otherwise in a credit line to the material. If material is not included in the article's Creative Commons license and your intended use is not permitted by statutory regulation or exceeds the permitted use, you will need to obtain permission directly from the copyright holder. To view a copy of this license, visit http://creativecommons.org/licenses/by/4.0/.

Reviewers' Comments:

Reviewer #1:
Remarks to the Author:
This study addressed a very important question regarding the complicated association between multiplexed environmental factors and aging. Using data from one of the largest cohorts (UK Biobank), this study captured the relative contributions of multiplexed environmental factors to aging and revealed the heterogeneity in the same environmental factors to multidimensional aging. A prominent strength of this study is the incorporation of multidimensional aging metrics and environmental measures, which allowed a holistic examination of the complex interaction between environmental factors and the aging process. Despite the significant strengths, this study suffers from several limitations as listed below:
1. This study included a series of analyses to examine the environment-aging associations. A poor logic structure and too detailed description for some unnecessary steps would sometimes confuse the readers about implementing these analyses. I strongly suggest the author clarify the crucial points for brevity.
2. I am a bit confused about the sample size used for each environmental factor or aging measure, especially for brain age estimation. Compared with other brain imaging measures (N>30k), the samples used for brain age were super small (N=3835). The flow chart of the analytic sample from the UK Biobank (Supporting figure 1) did not provide sufficient information about that. The authors should clarify it.
3. A large set of imaging and phenotypic measures were included in this study. I wonder whether UKB provided any specific IDs to explicitly identify these measures. Detailed information about this field identification would benefit better replication and validation for future studies.
4. This study included both multisystemic and domain-specific aging metrics. I would like to know whether these measures, such as brain age, were calculated by the authors or derived from previous studies. If the authors calculate this, details regarding the implementation should be provided. For example, in describing brain age, the authors said "brain age was estimated using machine learning based on a set of regional and global 198 features extracted from T1w sequences…". I think the specific machine learning methods should at least be specified.
5. As indicated by the authors, the environmental factors were assessed between 2007 and 2010. How about the aging markers? Were these measures also assessed at the same time, especially those related to brain imaging? It seems that UKB performed the imaging assessment after many years after the baseline behavior collection. In this context, did the author control for the time duration between baseline data collection and imaging assessment? The cognitive measures were also assessed multiple times. Which specific time point of data did the authors use?
6. In the WQS analysis, the author split the full data into a training and a validation subset. I did not find any results about this analysis. I would like to know did I missed anything.
7. The current study involved a large set of association analyses, how did the authors control for multiple comparisons?
8. In my opinion, circle plots in Figure 4 were not good options to show environment-aging associations. It is difficult to identify how each of the environmental factors relates to aging measures. There is a lack of figure legend to show the range of effect size for associations shown in this figure.

Reviewer #2:
Remarks to the Author:
This paper conducted a proof-of-concept study and successfully showed the heterogeneous associations of multiplexed environmental factors with multidimensional aging metrics. Findings suggest that various environmental factors could accelerate aging from multiple aspects. I have a few comments:

1. As the authors acknowledged, this is a cross-sectional study. However, UKB indeed has data to explore the longitudinal changes of the aging markers measured at different stages. For instance, the clinical traits for PhenoAge were also measured three times during the follow-up. Therefore, I would like to suggest a prospective study concerning baseline exposure and follow-up changes in aging markers.

2. Townsend deprivation index (nSES) is the most important variable that must be adjusted for in the models instead of iSES because it is highly related to the air pollution data of UKB generated by the LUR model. I strongly suggest the authors justify the main results by adjusting nSES. Current supplement results were not adequate.

3. I agree that WQS is a possible choice of analysis model. Nevertheless, since this study indeed conducted an association analysis between two sets of variables (i.e., exposure variables & aging variables), Canonical Correlation Analysis (CCA) could be a better choice.

4. Multiple comparison adjustments and mutual adjustment of exposures with impacts on aging in linear models must be considered in this study.

5. A flowchart showing the inclusion and exclusion of participants, as well as the detailed numbers of participants for each aging marker, is necessary.

6. The number of participants for each aging marker varied dramatically (Supplement Table S1), but it cannot be a reason for skipping other aging markers in SOM. I suggest the authors let readers know the SOM results of other aging markers besides PhenoAge, at least frailty and cognitive performance.

7. As an epidemiological study, authors may provide more discussion on the underlying biological mechanisms.

8. Code for the data analysis must be provided to reproduce the findings.

Reviewer #3:
Remarks to the Author:
The authors conducted a comprehensive investigation using the UKB national cohort study to explore intricate connections between multiplexed environmental factors and diverse aging metrics. Notably, the findings underscored that air pollution exerted the most significant impact in the majority of instances. The identification of five subpopulations with distinct environmental exposure patterns further enhanced the study's depth, revealing consistent associations with multidimensional aging metrics. Given the context of an aging population, the knowledge gained from this research holds substantial value and offers implications for the prevention and management of healthy aging.

The article demonstrates a commendable organizational structure and presentation. Nevertheless, I have some comments for the authors to consider:

1. Methods: The participants were recruited from 2006 to 2010, while most exposure data (e.g., NO_2, PM_{10}, green space, and road traffic noise) predates 2010. There may be temporal misclassifications between exposure and health outcomes. Kindly provide clarification.

2. Methods: While the WQS regression can calculate joint effects of multiplexed exposure, the authors opted for SOMs. Please clarify the advantages of SOMs over WQS.

3. Methods: Have the authors explored other statistical models, such as BKMR and G-computation, widely used in exploring joint and individual effects of multiplexed exposure?

4. Methods, Lines 287-289: Clarify the independent variable in the multiple linear regression models. Were participants categorized into five subgroups, and was this categorical variable introduced into the model? Also, specify the reference group.

5. Methods: Considering the detailed covariates collected by UKB, could the authors perform stratified analyses by sex, age, socioeconomic status, smoking status, etc., to explore potential heterogeneity among different subpopulations?

6. Figure 5A: Explain the meaning of the sector size. The current version might be confusing for readers unfamiliar with SOMs.

7. Discussion: Provide more explanations on heterogeneities where the same environmental factor contributed differently to various aging metrics.

8. Discussion: Acknowledge that the present study did not consider participants' mobility, which should be noted.

9. Line 415: Should Supplementary Table 19 also be included here?

Overall, the paper contributes significantly to the understanding of environmental factors in aging. Addressing these points will enhance the clarity and robustness of the study.

REVIEWER COMMENTS & RESPONSES

Dear Reviewers,

We sincerely appreciate the time and efforts you have dedicated to reviewing our manuscript. We have performed additional analyses and made changes to our manuscript in response to the comments from you. Point-by-point responses were made for each comment. We appreciate the excellent suggestions and comments from you. Because of the valuable comments, we believe that our manuscript has been improved. Our detailed responses to your comments are provided below.

Reviewer #1 (Remarks to the Author):

This study addressed a very important question regarding the complicated association between multiplexed environmental factors and aging. Using data from one of the largest cohorts (UK Biobank), this study captured the relative contributions of multiplexed environmental factors to aging and revealed the heterogeneity in the same environmental factors to multidimensional aging. A prominent strength of this study is the incorporation of multidimensional aging metrics and environmental measures, which allowed a holistic examination of the complex interaction between environmental factors and the aging process. Despite the significant strengths, this study suffers from several limitations as listed below:

1. This study included a series of analyses to examine the environment-aging associations. A poor logic structure and too detailed description for some unnecessary steps would sometimes confuse the readers about implementing these analyses. I strongly suggest the author clarify the crucial points for brevity.

Response: Thanks for your kind suggestion. We adjusted the structure of this article by moving the methods section to the end. We also moved the definitions of multiple covariates and individual socioeconomic status (iSES) into the Supplementary Method and the detailed results of additional analysis while adjusting for iSES to Supplementary Results to further improve the structure. We omitted cumbersome method statements (e.g., the detailed measurements of cognitive performance, Lines 502 to 509) in the revised manuscript. We have also tried our best to revise our expressions and language (e.g., Lines 54 to 56 and 125 to 138). If you feel that there are still some inappropriate expressions, pls let us know and we are very willing to further improve our manuscript.

2. I am a bit confused about the sample size used for each environmental factor or aging measure, especially for brain age estimation. Compared with other brain imaging measures (N>30k), the samples used for brain age were super small (N=3,835). The flow chart of the

analytic sample from the UK Biobank (Supporting Figure 1) did not provide sufficient information about that. The authors should clarify it.

Response: Thanks for your important note. We are sorry that we have not clearly stated the sample info in the previous submission. To address this issue, we have made these revisions, according to your suggestion:

1) We have reported the number of participants with data on each environmental factor and biomarkers used to calculate aging metrics in the revised supplementary file. We have also revised the flow chart (Supplementary Figure 1 as follows). We pointed out the exact number of participants included in analyses of each aging metric and listed the number of participants with missing data on required covariates to calculate each aging metric.

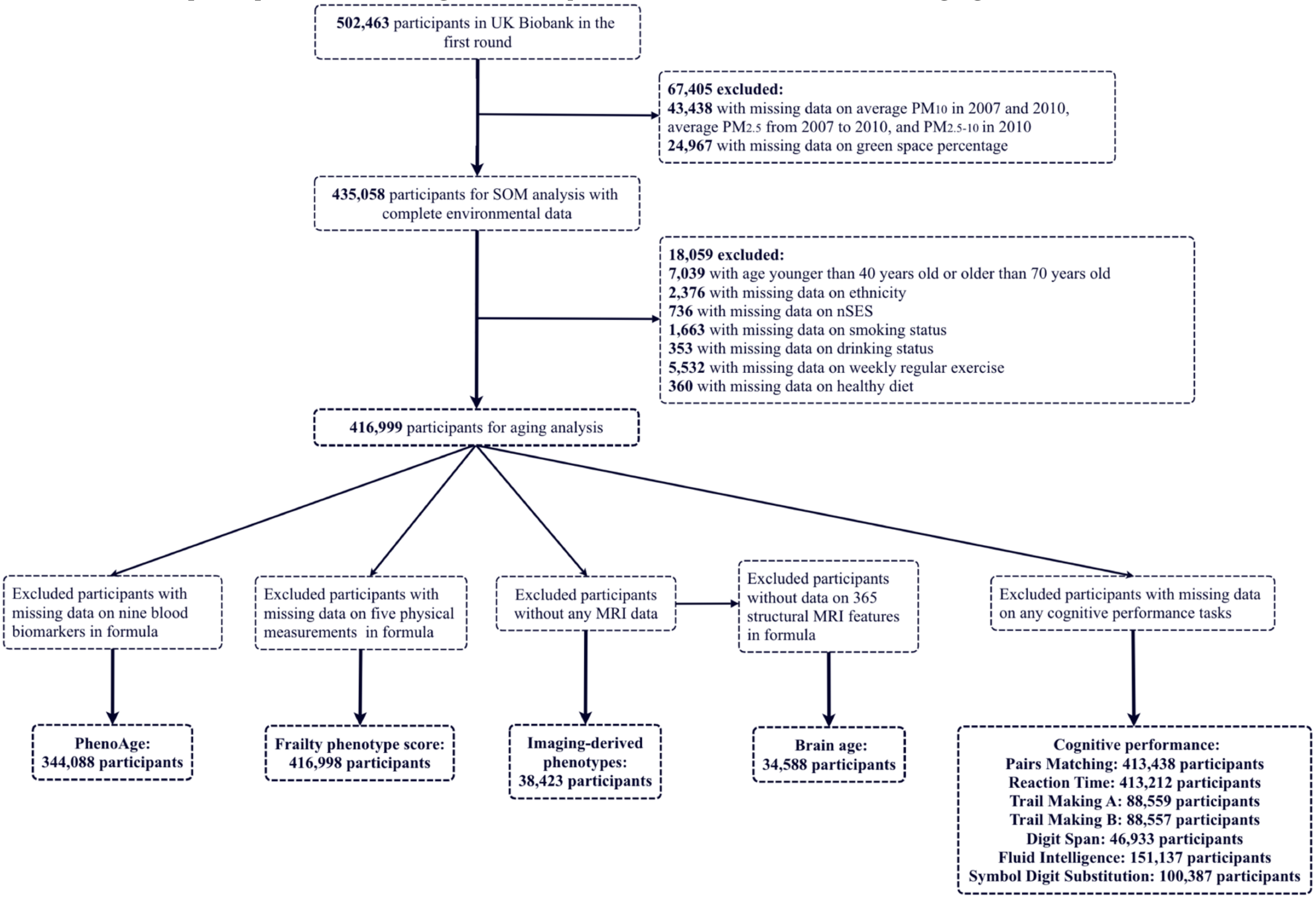

Supplementary Figure 1. Flow chart of analytic sample from the UK Biobank

Note: SOM, self-organizing map; PhenoAge, phenotypic age; MRI, magnetic resonance imaging; The number of participants with data on imaging-derived phenotypes of specific brain regions varied. 38,423 participants in the figure had data on imaging-derived phenotypes of all the included brain regions.

2) We only utilized the predicted brain age from the test dataset in the previous submission. After careful discussion, we added the brain age of the training dataset. Consequently, there were 34,588 participants enrolled in the analyses of brain age in the revised version. Accordingly, we have revised the related analyses, descriptions (e.g., Lines 489-499), tables (e.g., Supporting Tables 1 and 4), and figures (e.g., Figures 2 and 3).

3. A large set of imaging and phenotypic measures were included in this study. I wonder whether UKB provided any specific IDs to explicitly identify these measures. Detailed information about this field identification would benefit better replication and validation for future studies.

Response: Thanks for your significant suggestion. We have reported the specific IDs of the main variables included in our research in the revised Supplementary File. Providing these specific IDs would allow replication and validation for future studies.

4. This study included both multisystemic and domain-specific aging metrics. I would like to know whether these measures, such as brain age, were calculated by the authors or derived from previous studies. If the authors calculate this, details regarding the implementation should be provided. For example, in describing brain age, the authors said "brain age was estimated using machine learning based on a set of regional and global 198 features extracted from T1w sequences…". I think the specific machine learning methods should at least be specified.

Response: Thank you for pointing out that meaningful issue. No matter whether the aging metric was developed by ourselves or not, we have cited the original literature and provided computing methods briefly in the revised manuscript, according to your suggestion. For each aging metric, we respectively made explanations and related revisions as follows:

1) PhenoAge was developed by ourselves in the previous study[1]. PhenoAge was a novel comprehensive aging metric. Detailed descriptions and formulas were given in the revised manuscript (Lines 432 to 454).

2) Frailty phenotype score: a widely used physical frailty measurement proposed by Fried et al.[2] Details were described in the revised manuscript (Lines 457 to 467) and Supplementary Method with detailed descriptions of each physical measurement.

3) Brain age: a widely used index for quantifying individuals' brain health as deviation from a normative brain aging trajectory. Brain age was developed by Vidal-Pineiro's previous study[3]. We added descriptions in the revised manuscript: "In general, based on a set of regional and global features extracted from T1w sequences (including 365 structural magnetic resonance imaging features partitioned into 68 features of cortical thickness, area, and grey–white matter contrast, 66 features of cortical volume, 41 features of subcortical intensity, and 54 features of subcortical volume), the individuals' brain age was estimated using machine learning method, gradient tree boosting as implemented in XGBoost (https://xgboost.readthedocs.io) and optimized using 10-fold cross-validation and a randomized hyperparameter search[3]". (Lines 489 to 499)

4) Imaging-derived phenotypes: Through processing the raw brain MRI, multiple imaging-derived phenotypes (e.g., the whole brain volume and the whole grey volume) were directly provided by UKB in the form of a continuous variable.

5) Cognitive performance: We obtained cognitive performance measures on seven cognitive tasks from the UKB and processed them as previously described[4,5]. We described that in the revised manuscript (Lines 502 to 509) and Supplementary File with detailed descriptions of each cognitive test.

All the info above was provided in the revised manuscript accordingly.

5. As indicated by the authors, the environmental factors were assessed between 2007 and 2010. How about the aging markers? Were these measures also assessed at the same time, especially those related to brain imaging? It seems that UKB performed the imaging assessment after many years after the baseline behavior collection. In this context, did the author control for the time duration between baseline data collection and imaging assessment? The cognitive measures were also assessed multiple times. Which specific time point of data did the authors use?

Response: Thanks for your kind suggestion. To address this issue, we have presented different measurement time points of each aging metric and environmental factor in supplementary table 1 for better understanding, and we bolded the round we included in this study. For example, data on albumin used to calculate PhenoAge was collected in 2006-2010.

Considering the number of participants with available data and closer to the time of measurement of environmental factors, we chose data collected in 2006-2010/2014 for cognitive measurements.

UKB's main phase of baseline assessment is from 2006 to 2010 (about 500,000 participants enrolled) and launched baseline data in 2012 (https://biobank.ndph.ox.ac.uk/showcase/exinfo.cgi?src=baseline_data), which means the baseline data on physical indicators (e.g., blood biomarkers and physical function) were collected within several years between individuals. At present, research based on UKB assumes these data on the same baseline[6-8]. For environmental factors, utilizing averaged values between several years to represent certain environmental exposure levels is a common way in recent research focusing on multiplexed environmental factor[9-11]. Moreover, previous studies have shown that the spatial variation of air pollution and traffic noise exposure levels remain stable over periods of about 10 years in Western countries [12,13]. Moreover, utilizing brain imaging indicators directly is also common in recent exposure-brain research[14-17].

However, thanks for your heartfully reminder, we noticed this potential limitation, and then we have added related content in the revised manuscript "Although the data possessing was consistent with recent research, the time duration between different data collection,

especially that between baseline data collection and imaging assessment, may cause potential confounding effects.” (Lines 351 to 354)

6. In the WQS analysis, the author split the full data into a training and a validation subset. I did not find any results about this analysis. I would like to know did I missed anything.

Response: Thanks for pointing out the potential confusion in our study. Actually, during the model fitting process, the dataset was automatically divided into a 40% training set and a 60% validation set by the WQS package in R (validation = 0.6) (https://cran.r-project.org/web/packages/gWQS/vignettes/gwqs-vignette.html). The training set was utilized for weight estimation, while the validation set was used to test the significance of the WQS index. The final WQS index of this study was averaged from the weights in the 500 bootstrap samples[18]. Accordingly, we have added explanations in the revised manuscript (Lines 547 to 553).

7. The current study involved a large set of association analyses, how did the authors control for multiple comparisons?

Response: Thank you for the kind reminder. We have used Bonferroni correction to control the family-wise error in the present manuscript. All linear regressions in the main analyses and the subgroup analyses were put together for Bonferroni correction, respectively. The False Discovery Rate was controlled at 5% using the Benjamini–Hochberg procedure described elsewhere. The related explanation was added in the present manuscript: “For the volume of specific brain regions and cognitive performance, we used linear regression to preliminarily examine their association with multiplexed environmental factors. The False Discovery Rate was controlled at 5% across all linear regression models in the main analyses (n = 285) using the Benjamini–Hochberg procedure”. (Lines 538 to 542)

8. In my opinion, circle plots in Figure 4 were not good options to show environment-aging associations. It is difficult to identify how each of the environmental factors relates to aging measures. There is a lack of figure legend to show the range of effect size for associations shown in this figure.

Response: Thanks for your insightful suggestion. Accordingly, we have remade Figure 4 into a scatter plot. For the comparability between each brain volume index and each cognitive performance index, we normalized the data before regression analyses. The graph is shown as follows:

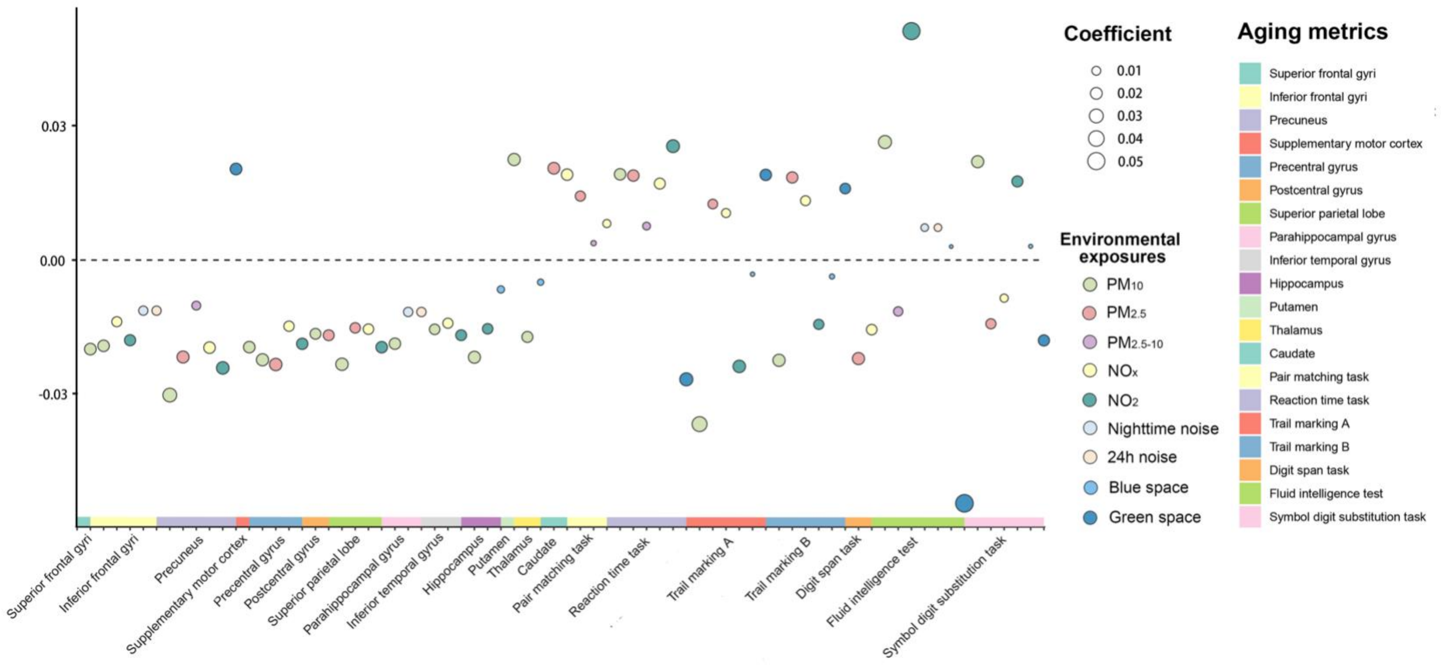

The Y-axis represents the regression coefficient of associations between environmental exposures and aging metrics. The sizes of plots also represent the sizes of coefficients.

Specifically, air pollution (particularly PM_{10}) accounted for the major contributions of variance in these brain factors and cognitive performance. Compared with other regions, the precuneus and postcentral gyrus were more influenced by multiplexed environmental factors. Green space showed considerable contributions to variance in cognitive performance (e.g., fluid intelligence test, digit span task, and trail making).

Reviewer #2 (Remarks to the Author):

This paper conducted a proof-of-concept study and successfully showed the heterogeneous associations of multiplexed environmental factors with multidimensional aging metrics. Findings suggest that various environmental factors could accelerate aging from multiple aspects. I have a few comments:

1. As the authors acknowledged, this is a cross-sectional study. However, UKB indeed has data to explore the longitudinal changes of the aging markers measured at different stages. For instance, the clinical traits for PhenoAge were also measured three times during the follow-up. Therefore, I would like to suggest a prospective study concerning baseline exposure and follow-up changes in aging markers.

Response: Thanks for your kind suggestion. As you mentioned, aging metrics could be calculated in UKB at different stages and we have presented different measurement time points of each aging metric in the Supplementary File for better understanding, and we bolded the round we included in this study. For example, data on albumin used to calculate PhenoAge was collected in 2006-2010.

Actually, we are eager to perform available longitudinal analyses. In this study, WQS and SOM were utilized to reduce the dimensionality and highly correlated (Figure 1), trying to disentangle the complex individual and joint contributions of the multiplexed environmental factors to aging. However, as we pointed out in the discussion, "effective methods to analyze the longitudinal contribution of baseline multiplexed environmental factors to subsequent health outcomes are urgently needed". Recently, there have been few effective methods to deal with the dimensionality and multicollinearity of multiplexed environmental factors in longitudinal analyses. If we use Generalized Estimating Equations, Linear Mixed Effects Model, or other traditional methods in longitudinal analyses, focusing on a single environmental factor once at a time, the results might be limited and biased, which makes them difficult to be interpreted and validated [as we explained in the discussion (Lines to 316 to 320)]. Consequently, through this study, we also further call for the necessity and urgency of developing effective statistical models that are valid in longitudinal analyses focusing on multiplexed environmental factors.

Moreover, although aging metrics were measured at different time points, the sample sizes of different aging metrics were almost significantly narrowed nearly 25 times in the second round. For example, the number of participants with available data on albumin, which was used to calculate PhenoAge, was 344,088 in the baseline and only 16,705 in the second-round survey in 2012-2013. It is clear that the results of longitudinal analyses in such a relatively small sample size may not be credible and robust.

As we have called for in the discussion (Lines 310 to 320), with better datasets and statistical methods, we believe that your insightful idea could be realized.

2. Townsend deprivation index (nSES) is the most important variable that must be adjusted for in the models instead of iSES because it is highly related to the air pollution data of UKB generated by the LUR model. I strongly suggest the authors justify the main results by adjusting nSES. Current supplement results were not adequate.

Response: Thank you for the important note. Accordingly, we reported the main results by adjusting for nSES in the revised manuscript (Lines 93 to 207). We have also adjusted related figures (e.g., Figures 3 to 5) and moved the previous iSES results into the Supplementary Results.

Generally, the results when adjusting for nSES were similar to the results when adjusting for iSES. Specifically, PhenoAge had a significant positive association with multiplexed environmental factors ($\beta = 0.043$; 95% CI: 0.014, 0.072, Supplementary Table 4), and PM_{10} was predominant, with a weight of 40.8%, as a major contributor among the multiplexed environmental factors to the variance in PhenoAge. Meanwhile, the weight of $PM_{2.5-10}$ (40.4%) indicated its second-largest contribution to the variance in PhenoAge. The relative contributions were similar in brain age (PM_{10} contributed 37.2% and $PM_{2.5}$ contributed 32.5%).

According to the SOM results, the air pollution subpopulation consistently had a worse aging status; for example, they had the highest frailty phenotype score ($\beta = 0.045$; 95% CI: 0.037, 0.053), and the worst brain age ($\beta = 0.133$; 95% CI: 0.053, 0.213). The noise subpopulation represented the second worst aging status in gray matter volume ($\beta = -0.045$; 95% CI: -0.069, -0.021) and the whole brain volume ($\beta = -0.052$; 95% CI: -0.085, -0.019), which might imply possible interactive negative effect between air pollution and noise on brain aging.

3. I agree that WQS is a possible choice of analysis model. Nevertheless, since this study indeed conducted an association analysis between two sets of variables (i.e., exposure variables & aging variables), Canonical Correlation Analysis (CCA) could be a better choice.

Response: Thanks for your advice. As you mentioned, we indeed tried to disentangle the association between two sets of variables (i.e., exposure variables & aging variables). However, we suppose that CCA has several limitations when it was applied to this study:

1) Multicollinearity Issues: CCA is affected by high correlations among variables in the analysis. This multicollinearity leads to instability and reduced interpretability of the results. As we reported, air pollution was strongly positively correlated with each other ($\rho s > 0.60$, Figure 2). That situation might reduce the efficiency and accuracy of CCA. Comparatively, WQS could construct a weighted index of multiplexed environmental factors to reduce dimensionality and remediate the collinearity problem of conventional regression models[19].

2) Interpretation Challenges: The results of CCA can be difficult to be interpreted, especially when involving a large number of variables. It can produce complex canonical variables and correlations that are hard to understand. CCA identifies common variations

in two sets of high-dimensional variables. Considering the heterogeneous aging across multiple organ systems,[7] we did not treat multidimensional aging metrics as a set, but separately put them into analyses, aiming to find potential different effects from external environmental factors on various aging dimensions. WQS could provide both the total effect of multiplexed environmental factors on a specific aging metric and the relative contribution of each environmental factor.

3) Relatively small sample size: the dataset used for CCA is not allowed to have missing values. However, the sample size for participants with complete data in two sets of variables (i.e., exposure variables & aging variables) was only 1,197, which was relatively small compared to sample sizes for WQS. These deleted data in CCA can distort potential associations, affecting the accuracy and reliability of the analysis.

In short, when considering the above issues, WQS would address our research questions better relative to CCA. if you still suppose that we should perform CCA, we are very willing to add the corresponding analysis in our study.

4. Multiple comparison adjustments and mutual adjustment of exposures with impacts on aging in linear models must be considered in this study.

Response: Thank you for the kind reminder. We have used Bonferroni correction to control the family-wise error in the revised manuscript. All linear regressions in the main analyses and the subgroup analyses were put together for Bonferroni correction, respectively. The False Discovery Rate was controlled at 5% using the Benjamini–Hochberg procedure described elsewhere. The related explanation was added in the revised manuscript (Lines 538 to 542): "The False Discovery Rate was controlled at 5% across all linear regression models in the main analyses (n = 285) using the Benjamini–Hochberg procedure".

5. A flow chart showing the inclusion and exclusion of participants, as well as the detailed numbers of participants for each aging marker, is necessary.

Response: Thanks for your advice. We have revised the flow chart (**Supplementary** Figure 1 as follows). We pointed out the exact number of participants included in analyses of each aging metric and listed the number of participants with missing data on required covariates to calculate each aging metric.

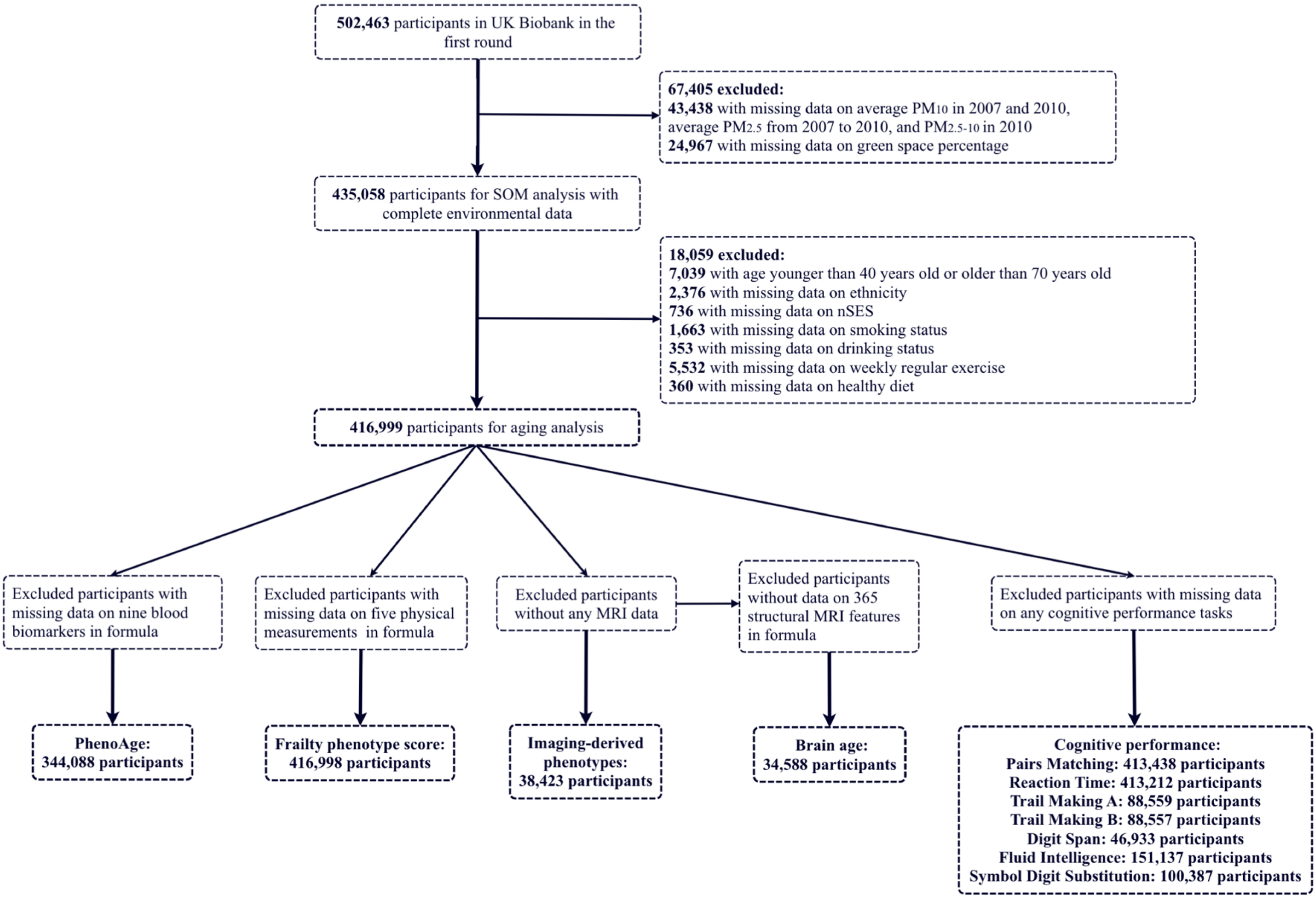

Supplementary Figure 1. Flow chart of analytic sample from the UK Biobank

Note: SOM, self-organizing map; PhenoAge, phenotypic age; MRI, magnetic resonance imaging; The number of participants with data on imaging-derived phenotypes of specific brain regions varied. 38,423 participants in the figure had data on imaging-derived phenotypes of all the included brain regions.

6. The number of participants for each aging marker varied dramatically (Supplement Table S1), but it cannot be a reason for skipping other aging markers in SOM. I suggest the authors let readers know the SOM results of other aging markers besides PhenoAge, at least frailty and cognitive performance.

Response: Thanks for your important note. Aiming to capture general environmental exposure patterns in a large dimension, we performed SOM in participants with data on multiplexed environmental factors before excluding any missing data on multiple aging metrics (e.g., PhenoAge and frailty phenotype score) and any covariates (e.g., BMI, and smoking status).

Accordingly, we have deleted the mistakenly written sentence: “In this study, considering PhenoAge as a multisystemic aging metric and its relatively large sample size, we used the participants included in the analyses of PhenoAge as a representative for further

subdivision” in the revised manuscript. We added that “Aiming to capture general environmental exposure patterns in a large dimension, we performed SOM in participants with complete data on environmental factors” (Lines 563 to 565).

7. As an epidemiological study, authors may provide more discussion on the underlying biological mechanisms.

Response: Thank you for the insightful note. Accordingly, we have added more discussions on the underlying biological mechanisms in the revised manuscript: “…Such heterogeneity further stressed the complexity of the aging process and the different biological mechanisms of how environmental factors may affect the aging process. First, recent studies have reported differentiated aging clocks and biomarkers involving various organs (e.g., liver and kidney) and systems (e.g., immune systems and metabolic system)[7,20,21], which imply that there might be systemic aging drivers/clocks overlaid with organ/tissue-specific counterparts[20], exhibiting complicated interactions. Second, taking road traffic noise as an example, we found that it showed more considerable contributions to the brain aging process (e.g., brain age acceleration and gray volumes decrease). When noise travels through the auditory pathway to the brain, it triggers the paraventricular Nucleus (PVN) of the hypothalamus to release corticotrophin-releasing hormone (CRH). This process leads to the activation of Proinflammatory cytokines and oxidative stress, which in turn stimulate the synthesis, secretion, and neurotoxicity of neurotransmitters. Additionally, many studies have demonstrated that the brain's susceptibility to noise can be attributed to the impact of stress on the higher cortical and limbic structures[22,23]. Besides, several genes (e.g., SOX2[24,25], iNOS[26], and NXN[27,28]) were significantly associated with exposure to specific air pollutants (e.g., $PM_{2.5}$ and PM_{10}), which may further influence various biological pathways (e.g., insulin resistance and TNF signaling pathway)[29]. Further, asynchronous inter- and intra-organ gene expression during the aging process[21,30] could be differentially affected by specific pathways. Future studies are poised to more systematically unravel the heterogeneous contributions of specific exposures to different aging pathways and molecules.” (Lines 267 to 288)

8. Code for the data analysis must be provided to reproduce the findings.
Response: Thank you for the suggestion. To address this concern, we have provided the code file on https://github.com/SiriusPu/Heterogeneous-associations-of-multiplexed-environmental-factors-and-multidimensional-aging-metrics. We also added related information in the revised manuscript, “All analyses were performed in R version 4.2.1 (http://www. R-project.org). The code used for WQS and SOM analyses is an adaptation of the R package ‘gWQS’ and ‘kohonen’ (https://github.com/blewy/Self_Organizing_maps_R), respectively, and has been made available through the GitHub repository: https://github.com/SiriusPu/Heterogeneous-associations-of-multiplexed-environmental-factors-and-multidimensional-aging-metrics. The code to calculate the brain age is available at https://github.com/LCBC-

UiO/VidalPineiro_ BrainAge." (Lines 489 to 499)

Reviewer #3 (Remarks to the Author):

The authors conducted a comprehensive investigation using the UKB national cohort study to explore intricate connections between multiplexed environmental factors and diverse aging metrics. Notably, the findings underscored that air pollution exerted the most significant impact in the majority of instances. The identification of five subpopulations with distinct environmental exposure patterns further enhanced the study's depth, revealing consistent associations with multidimensional aging metrics. Given the context of an aging population, the knowledge gained from this research holds substantial value and offers implications for the prevention and management of healthy aging.

The article demonstrates a commendable organizational structure and presentation. Nevertheless, I have some comments for the authors to consider:

1. Methods: The participants were recruited from 2006 to 2010, while most exposure data (e.g., NO_2, PM_{10}, green space, and road traffic noise) predates 2010. There may be temporal misclassifications between exposure and health outcomes. Kindly provide clarification.

Response: Thanks for your kind comment. Please see our response to Reviewer #1 Q5.

2. Methods: While the WQS regression can calculate joint effects of multiplexed exposure, the authors opted for SOMs. Please clarify the advantages of SOMs over WQS.

Response: Thanks for pointing out that important issue. There were two main reasons for applying SOM in this study:

1) WQS can identify individual effects while controlling for other mixture components, as well as estimate an overall mixture effect on the outcome. However, WQS assumes that all components are constrained to have the same direction of association (positive or inverse) with the outcome. Thus, the overall mixture effect may not be informative, for components of the mixture act in different directions, e.g., if green space has a protective effect on the outcome, while air pollution has a harmful effect.

Served as an unsupervised method, SOM creates a low dimensional projection of class profiles (i.e. clusters) by grouping observations with similar exposure profiles, without influencing the effect of environmental factors on aging. In this case, it may be useful to explore the joint effects of an exposure mixture using a clustering method such as SOM with subsequent linear regression models.

2) In this study, SOM was used to identify subpopulations with the same environmental exposure characteristics, which WQS cannot realize,[59,60] representing the homogenous and heterogeneous environmental exposure patterns among the population. Thus, we could

further match subpopulations to the geographic map of the UK and discuss more real-world insights in the revised manuscript (Lines 242 to 260).

3. Methods: Have the authors explored other statistical models, such as BKMR and G-computation, widely used in exploring joint and individual effects of multiplexed exposure?

Response: Thank you for your insightful and valuable suggestion. In our study, we first examined the effect direction of each environmental factor on aging metrics based on results of linear regression and accordingly, we specifically adjusted for the exposure indices for green and blue spaces to ensure that their effect directions are consistent with other pollutants, while we have operated WQS under the assumption that all exposures have a consistent direction of effect on the outcome. Under this assumption, there is no fundamental difference between the WQS model and G-computation models. Meanwhile, we have operated linear regression models between each environmental factor and each aging metric. The linear regression results were largely consistent with WQS analysis. Moreover, although we found that air pollution exerted the most significant impact in the majority of instances, the heterogeneity within the associations between multiplexed environmental factors and multidimensional aging metrics, which we have reported, was nonnegligible. We sincerely hope that this study can reveal the heterogeneous associations of multiplexed environmental factors with multidimensional aging metrics and serve as a proof of concept when analyzing multifactors and multiple outcomes, but we do not want too many statistical methods to lead to the accumulation of results, reducing readability and interpretation.

Moreover, the limitation of BKMR arises from its computational intensity, primarily due to reliance on Markov Chain Monte Carlo (MCMC) simulations for estimation. While BKMR can perform parallel computation across multiple chains, but does not support efficient parallel processing within individual chains. This computational bottleneck becomes particularly problematic for large datasets, such as ours with over 300,000 participants, where the required computational resources and time can be prohibitive. Current applications of BKMR in environmental exposure research typically involve sample sizes up to approximately 10,000[31,32], highlighting the challenge of scaling this method to larger cohorts.

If limit our sample size to around 10,000 (about 30 times smaller), then our population representativeness will be poor, and the results may be biased. Consequently, there is a pressing need for the development of more computationally efficient statistical methodologies capable of handling the increasing data dimensions in large sample sizes as we discussed in the revised manuscript (Lines 316 to 318).

4. Methods, Lines 287-289: Clarify the independent variable in the multiple linear regression models. Were participants categorized into five subgroups, and was this categorical variable introduced into the model? Also, specify the reference group.

Response: Thank you for your kind advice. Accordingly, we have revised the description in the revised manuscript: “Subpopulation’s multidimensional aging metrics were

compared (vs. the green space subpopulation) using multiple linear regression models, adjusting for all covariates" (Lines 566 to 569).

5. Methods: Considering the detailed covariates collected by UKB, could the authors perform stratified analyses by sex, age, socioeconomic status, smoking status, etc., to explore potential heterogeneity among different subpopulations?

Response: Thank you for the opportunity to further clarify this important point. We have performed stratified analyses by sex, age, smoking status, and alcohol intake frequency. The detailed results are reported in the Supplementary Tables 15 to 22.

Accordingly, we added related descriptions in the revised manuscript as follows:

1) Method

"...Considering the potential heterogeneity among different subpopulations, we performed stratified analyses by sex, age, smoking status, and alcohol drink frequency..."

2) Results (Lines 191 to 207)

"According to the stratified analyses (Supplementary Tables 15-22), overall, air pollution remained the most important contributor to multidimensional aging metrics. Males seemed to be a vulnerable subpopulation in which each aging metric was significantly associated with multiplexed environmental factors. In males, PM_{10} made the dominant contribution to PhenoAge (45.5%), white matter volume (54.5%), and brain volume (52.7%). Meanwhile, heavy drinkers (drinking daily or almost daily) were more likely to be influenced by multiplexed environmental factors, especially for their brain volumes. For example, multiplexed environmental factors were significantly associated with the whole brain volume among heavy drinkers (β = -3505.70; 95% CI: -5730.89, -1280.51) and moderate drinkers (drinking one to four times per week) (β = -2010.94; 95% CI: -3342.82, -678.98).

Compared to the green space subpopulation, the air pollution subpopulation remained to have the worst aging status in most aging metrics, especially for frailty phenotype score and the rural-urban subpopulation also showed a worse aging status in stratified analyses. For example, among participants with age ≥ 60 years, both the air pollution subpopulation (β = 0.047; 95% CI: 0.035, 0.059) and the rural-urban subpopulation (β = 0.042; 95% CI: 0.032, 0.052) had a higher frailty phenotype score."

6. Figure 5A: Explain the meaning of the sector size. The current version might be confusing for readers unfamiliar with SOMs.

Response: Thanks for your warm suggestion. Accordingly, we have added descriptions in the revised footnotes of Figure 5: "A larger sector size represents the larger amount of a specific environmental exposure". (Lines 130 to 131)

7. Discussion: Provide more explanations on heterogeneities where the same environmental factor contributed differently to various aging metrics.

Response: Thanks for mentioning this point. Actually, this was the interesting part of this study. As we stated in the revised manuscript, "... As a multifaceted process with variation among individuals and organs[7], aging is unlikely to be interpreted by a single-dimensional aging metric..." (Lines 262 to 264), could be one explanation. Also, pls see our response to Reviewer #2 Q7, we added more discussions on the underlying biological mechanisms in the revised manuscript, e.g., to explain how aging is complicated. For instance, we know that various aging metrics used in this study focus on multisystemic and domain-specific dimensions, "our study revealed heterogeneity in the relative contributions of the same environmental factors to multidimensional aging. Such heterogeneity further stressed the complexity of the aging process and the different biological mechanisms of how environmental factors may affect the aging process. First, recent studies have reported differentiated aging clocks and biomarkers involving various organs (e.g., liver and kidney) and systems (e.g., immune systems and metabolic system) which imply that there might be systemic aging drivers/clocks overlaid with organ/tissue-specific counterparts, exhibiting complicated interactions..." (Lines 265 to 273). These additions would further strengthen the discussion part.

8. Discussion: Acknowledge that the present study did not consider participants' mobility, which should be noted.

Response: Thank you for your valuable comment. However, after checking the UK Biobank covariates list, we found that UKB did not state whether they would update participants' home location (Data-Field 22702, version 2017). Thus, we added the note of this limitation in the revised manuscript: "Sixth, UKB did not provide information to capture participant's mobility, which may bias the home location-based measurement of environmental exposure to some extent". (Lines 354 to 356)

9. Line 415: Should Supplementary Table 19 also be included here?

Response: Corrected accordingly.

10. Overall, the paper contributes significantly to the understanding of environmental factors in aging. Addressing these points will enhance the clarity and robustness of the study.

Response: Thanks so much for your positive comment.

Reference

1 Biobank, U. *Category 10080: blood assays—biological samples*, <https://biobank.ndph.ox.ac.uk/showcase/label.cgi?id=100080> (

2 Fried, L. P. *et al.* Frailty in older adults: evidence for a phenotype. *J Gerontol A Biol Sci Med Sci* **56**, M146-156 (2001). https://doi.org:10.1093/gerona/56.3.m146

3 Vidal-Pineiro, D. *et al.* Individual variations in 'brain age' relate to early-life factors more than to longitudinal brain change. *Elife* **10** (2021). https://doi.org:10.7554/eLife.69995

4 Kendall, K. M. *et al.* Cognitive performance and functional outcomes of carriers of pathogenic copy number variants: analysis of the UK Biobank. *Br J Psychiatry* **214**, 297-304 (2019). https://doi.org:10.1192/bjp.2018.301

5 Kendall, K. M. *et al.* Cognitive Performance Among Carriers of Pathogenic Copy Number Variants: Analysis of 152,000 UK Biobank Subjects. *Biol Psychiatry* **82**, 103-110 (2017). https://doi.org:10.1016/j.biopsych.2016.08.014

6 Kanki, M. *et al.* Poor sleep and shift work associate with increased blood pressure and inflammation in UK Biobank participants. *Nature Communications* **14**, 7096 (2023). https://doi.org:10.1038/s41467-023-42758-6

7 Tian, Y. E. *et al.* Heterogeneous aging across multiple organ systems and prediction of chronic disease and mortality. *Nat Med* **29**, 1221-1231 (2023). https://doi.org:10.1038/s41591-023-02296-6

8 Gao, X. *et al.* Accelerated biological aging and risk of depression and anxiety: evidence from 424,299 UK Biobank participants. *Nature Communications* **14**, 2277 (2023). https://doi.org:10.1038/s41467-023-38013-7

9 Zhang, Y. *et al.* Identifying modifiable factors and their joint effect on dementia risk in the UK Biobank. *Nat Hum Behav* **7**, 1185-1195 (2023). https://doi.org:10.1038/s41562-023-01585-x

10 Klompmaker, J. O. *et al.* Associations of Combined Exposures to Surrounding Green, Air Pollution, and Road Traffic Noise with Cardiometabolic Diseases. *Environ Health Perspect* **127**, 87003 (2019). https://doi.org:10.1289/ehp3857

11 Li, T. *et al.* Residential greenness, air pollution, and incident ischemic heart disease: A prospective cohort study in China. *Sci Total Environ* **838**, 155881 (2022). https://doi.org:10.1016/j.scitotenv.2022.155881

12 Fecht, D. *et al.* Spatial and temporal associations of road traffic noise and air pollution in London: Implications for epidemiological studies. *Environment International* **88**, 235-242 (2016). https://doi.org:https://doi.org/10.1016/j.envint.2015.12.001

13 Marloes, E. *et al.* Stability of measured and modelled spatial contrasts in NO₂ over time. *Occupational and Environmental Medicine* **68**, 765 (2011). https://doi.org:10.1136/oem.2010.061135

14 Gray, J. C. *et al.* Associations of cigarette smoking with gray and white matter in the UK Biobank. *Neuropsychopharmacology* **45**, 1215-1222 (2020). https://doi.org:10.1038/s41386-020-0630-2

15 Daviet, R. *et al.* Associations between alcohol consumption and gray and white matter volumes in the UK Biobank. *Nature Communications* **13**, 1175 (2022). https://doi.org:10.1038/s41467-022-28735-5

16 Li, Y. *et al.* The brain structure and genetic mechanisms underlying the nonlinear association

between sleep duration, cognition and mental health. *Nature Aging* **2**, 425-437 (2022). https://doi.org:10.1038/s43587-022-00210-2
17 Zhao, Y. *et al.* The brain structure, immunometabolic and genetic mechanisms underlying the association between lifestyle and depression. *Nature Mental Health* **1**, 736-750 (2023). https://doi.org:10.1038/s44220-023-00120-1
18 Zhang, S., Tang, H. & Zhou, M. Sex-specific associations between nine metal mixtures in urine and urine flow rate in US adults: NHANES 2009-2018. *Front Public Health* **11**, 1241971 (2023). https://doi.org:10.3389/fpubh.2023.1241971
19 Czarnota, J., Gennings, C. & Wheeler, D. C. Assessment of weighted quantile sum regression for modeling chemical mixtures and cancer risk. *Cancer Inform* **14**, 159-171 (2015). https://doi.org:10.4137/cin.S17295
20 Nie, C. *et al.* Distinct biological ages of organs and systems identified from a multi-omics study. *Cell Rep* **38**, 110459 (2022). https://doi.org:10.1016/j.celrep.2022.110459
21 Schaum, N. *et al.* Ageing hallmarks exhibit organ-specific temporal signatures. *Nature* **583**, 596-602 (2020). https://doi.org:10.1038/s41586-020-2499-y
22 Arnsten, A. F. & Goldman-Rakic, P. S. Noise stress impairs prefrontal cortical cognitive function in monkeys: evidence for a hyperdopaminergic mechanism. *Arch Gen Psychiatry* **55**, 362-368 (1998). https://doi.org:10.1001/archpsyc.55.4.362
23 McEwen, B. S., Weiss, J. M. & Schwartz, L. S. Selective retention of corticosterone by limbic structures in rat brain. *Nature* **220**, 911-912 (1968). https://doi.org:10.1038/220911a0
24 Tantoh, D. M. *et al.* SOX2 promoter hypermethylation in non-smoking Taiwanese adults residing in air pollution areas. *Clin Epigenetics* **11**, 46 (2019). https://doi.org:10.1186/s13148-019-0647-8
25 Su, C. L. *et al.* Blood-Based SOX2-Promoter Methylation in Relation to Exercise and PM(2.5) Exposure among Taiwanese Adults. *Cancers (Basel)* **12** (2020). https://doi.org:10.3390/cancers12020504
26 Tarantini, L. *et al.* Effects of particulate matter on genomic DNA methylation content and iNOS promoter methylation. *Environ Health Perspect* **117**, 217-222 (2009). https://doi.org:10.1289/ehp.11898
27 Panni, T. *et al.* Genome-Wide Analysis of DNA Methylation and Fine Particulate Matter Air Pollution in Three Study Populations: KORA F3, KORA F4, and the Normative Aging Study. *Environ Health Perspect* **124**, 983-990 (2016). https://doi.org:10.1289/ehp.1509966
28 Eze, I. C. *et al.* Genome-Wide DNA Methylation in Peripheral Blood and Long-Term Exposure to Source-Specific Transportation Noise and Air Pollution: The SAPALDIA Study. *Environ Health Perspect* **128**, 67003 (2020). https://doi.org:10.1289/ehp6174
29 Wu, Y. *et al.* Air pollution and DNA methylation in adults: A systematic review and meta-analysis of observational studies. *Environ Pollut* **284**, 117152 (2021). https://doi.org:10.1016/j.envpol.2021.117152
30 Ahadi, S. *et al.* Personal aging markers and ageotypes revealed by deep longitudinal profiling. *Nat Med* **26**, 83-90 (2020). https://doi.org:10.1038/s41591-019-0719-5
31 Christensen, G. M. *et al.* The complex relationship of air pollution and neighborhood socioeconomic status and their association with cognitive decline. *Environ Int* **167**, 107416 (2022). https://doi.org:10.1016/j.envint.2022.107416
32 Li, G. *et al.* Multiple environmental exposures and obesity in eastern China: An individual

exposure evaluation model. *Chemosphere* **298**, 134316 (2022). https://doi.org:10.1016/j.chemosphere.2022.134316

Reviewers' Comments:

Reviewer #1:
Remarks to the Author:
The authors have addressed all my concerns. I have no further comments.

Reviewer #2:
Remarks to the Author:
The authors addressed most of my comments well. Two more comments:

1) The authors may know that environmental exposures are not only air pollution, noise, and g/b space. For instance, night light, indoor air pollution, and temperature are also critical to aging. Authors must give the rationale for studying the selected exposure in the introduction.

2) UKB has the data on relocation (Field ID 699). Please do consider a sensitivity in people living in the current location for at least 3 or 5 years. In other words, the last limitation is not applicable to this study

Reviewer #3:
Remarks to the Author:
I am pleased to see that the authors have addressed the concerns I previously raised. I have only two minor suggestions:

1. Figure 4: Please clarify whether only associations with a FDR less than 0.05 were displayed.

2. Lines 354-358: It seems that the sixth and final limitations express the same idea. Kindly delete one accordingly.

REVIEWER COMMENTS & RESPONSES

Dear Reviewers,

We extend our deepest gratitude for your insightful and constructive comments on our manuscript. In response to your invaluable comments, we have conducted further analyses and made revisions to our manuscript. We believe these changes have further improved our manuscript. Our detailed responses to your comments are provided below.

Reviewer #1 (Remarks to the Author):

The authors have addressed all my concerns. I have no further comments.

Response: Thank you very much for your previous insightful comments.

Reviewer #2 (Remarks to the Author):

The authors addressed most of my comments well. Two more comments:

1. The authors may know that environmental exposures are not only air pollution, noise, and g/b space. For instance, night light, indoor air pollution, and temperature are also critical to aging. Authors must give the rationale for studying the selected exposure in the introduction.

Response: Thanks for your important note. We fully agree that there are many types of environmental exposures, such as night light, indoor air pollution, and temperature, which influence aging. As you may know, we have incorporated indicators from each dimension of environmental factors provided by the UK Biobank (i.e., air pollution, green and blue spaces, and noise). Unfortunately, the UK Biobank does not provide data on other environmental factors like night light and indoor air pollution. Thus, as you suggested, we have made a corresponding revision in the introduction: "This study aimed to 1) examine the complex association of multiplexed environmental factors available in the UK Biobank (i.e., air pollution, green and blue spaces, and noise) with multidimensional aging metrics to determine their relative contributions" (Line 79).

Additionally, we have added a discussion on this limitation: "Second, the data on more environmental exposures such as night light, indoor air pollution, and temperature, which may be also critical to aging, were not available in the UK Biobank. Future studies are needed to depict more comprehensive individual environmental factors." (Lines 363 to 366).

2. UKB has the data on relocation (Field ID 699). Please do consider a sensitivity in people living in the current location for at least 3 or 5 years. In other words, the last limitation is not applicable to this study.

Response: Thanks for your significant suggestion. Accordingly, we performed additional analyses after eliminating individuals living in the current location for less than five years. The results largely

remained the same (Supplementary Tables 14 to 15). Particular matters and nitrogen oxides continued to show the dominant contribution to multidimensional aging metrics in WQS models. The air pollution subpopulation had the worst aging status.

We have revised the present manuscript, “Similarly, after eliminating participants living in the current location for less than five years, the results still largely remained the same (Supplementary Tables 14 - 15)” (Lines 205 to 207) and “Given the participant’s mobility, which may bias the home location-based measurement of environmental exposure to some extent, we repeated the main analysis after eliminating participants living in the current location for less than five years ” (Lines 609 to 611)

Reviewer #3 (Remarks to the Author):

I am pleased to see that the authors have addressed the concerns I previously raised. I have only two minor suggestions:

1. Figure 4: Please clarify whether only associations with a FDR less than 0.05 were displayed.

Response: Thanks for your kind suggestion. We have revised the present manuscript accordingly, “Only associations with an FDR less than 0.05 were displayed” (Lines 135 to 136).

2. Lines 354-358: It seems that the sixth and final limitations express the same idea. Kindly delete one accordingly.

Response: Thanks for pointing out the potential confusion in our manuscript. Please see our response to Reviewer #2 Q2. We have already performed further analyses to address the issue on participants’ mobility and deleted the redundant limitations.
